# Characteristics of Medical Deserts and Approaches to Mitigate Their Health Workforce Issues: A Scoping Review of Empirical Studies in Western Countries

**DOI:** 10.34172/ijhpm.2023.7454

**Published:** 2023-08-15

**Authors:** Linda E. Flinterman, Ana I. González-González, Laura Seils, Julia Bes, Marta Ballester, Joaquim Bañeres, Sorin Dan, Alicja Domagala, Katarzyna Dubas-Jakóbczyk, Robert Likic, Marieke Kroezen, Ronald Batenburg

**Affiliations:** ^1^Health Workforce and Organization Studies, Netherlands Institute for Health Services Research (NIVEL), Utrecht, The Netherlands; ^2^Avedis Donabedian Research Institute – UAB, Madrid, Spain; ^3^Innovation and Entrepreneurship InnoLab, University of Vaasa, Vaasa, Finland; ^4^Department of Health Policy and Management, Institute of Public Health, Jagiellonian University, Krakow, Poland; ^5^Department of Health Economics and Social Security, Institute of Public Health, Jagiellonian University, Krakow, Poland; ^6^School of Medicine, University of Zagreb, Zagreb, Croatia; ^7^Trimbos Institute, Netherlands Institute of Mental Health and Addiction, Utrecht, The Netherlands; ^8^Department of Sociology, Radboud University, Nijmegen, The Netherlands

**Keywords:** Medical Desert, Health Workforce, Definition, Characteristics, Contributing Factors, Approaches

## Abstract

**Background:** Medical deserts are considered a problematic issue for many Western countries which try to employ multitude of policies and initiatives to achieve a better distribution of their health workforce (HWF). The aim of this study was to systematically map research and provide an overview of definitions, characteristics, contributing factors and approaches to mitigate medical deserts within the European Union (EU)-funded project "ROUTE-HWF" (a Roadmap OUT of mEdical deserts into supportive Health WorkForce initiatives and policies).

**Methods:** We performed a scoping review to identify knowledge clusters/research gaps in the field of medical deserts focusing on HWF issues. Six databases were searched till June 2021. Studies reporting primary research from Western countries on definitions, characteristics, contributing factors, and approaches were included. Two independent reviewers assessed studies for eligibility, extracted data and clustered studies according to the four defined outcomes.

**Results:** Two-hundred and forty studies were included (n=116, 48% Australia/New Zealand; n=105, 44% North America; n=20, 8% Europe). All used observational designs except for five quasi-experimental studies. Studies provided definitions (n=171, 71%), characteristics (n=95, 40%), contributing factors (n=112, 47%), and approaches to mitigate medical deserts (n=87, 36%). Most medical deserts were defined by the density of the population in an area. Contributing factors to HWF issues in medical deserts consisted in work-related (n=55, 23%) and lifestyle-related factors (n=33, 14%) of the HWF as well as sociodemographic characteristics (n=79, 33%). Approaches to mitigate them focused on training adapted to the scope of rural practice (n=67, 28%), HWF distribution (n=3, 1%), support/infrastructure (n=8, 3%) and innovative models of care (n=7, 3%).

**Conclusion:** Our study provides the first scoping review that presents and categorizes definitions, characteristics, contributing factors, and approaches to mitigate HWF issues in medical deserts. We identified gaps such as the scarcity of longitudinal studies to investigate the impact of factors contributing to medical deserts, and interventional studies to evaluate the effectiveness of approaches to mitigate HWF issues.

## Background

 Medical deserts are known by many names. While the World Health Organization (WHO) defined “underserved areas” as “geographical areas where populations have limited access to qualified healthcare providers and quality healthcare services,”^1^ such definition varies by country.^2^ Also, the definition of “medical deserts”^3^ itself is not uniform, as countries differ in their geographical characteristics (eg, islands or mountain areas), what is considered as a “rural and remote area,”^4^ and in terms of the type of health workforce (HWF) that is undersupplied (eg, medical specialists, dentists, etc), respectively. Therefore, there is a lack of understanding on medical deserts in the absence of a clear definition and categorization – which causes confusion in both research and policy discourse leading to misleading comparisons.^5^

 Medical deserts are increasingly considered a problematic issue for many countries which try to employ a multitude of policies, actions and initiatives to achieve a better distribution of the HWF.^6^ WHO’s Regional Office for Europe underlined the severity of the problem in its recent European Programme of Work 2020-2025, and considered medical deserts as the main HWF priority for Europe.^7^

 A maldistribution of the HWF can have severe negative effects. A systematic review confirmed this fact by finding strong evidence for an association between health outcomes and patient travel time: the further away patients lived from the healthcare facility they needed to attend, the worse were their health outcomes (eg, survival rates, length of stay in hospital, and non-attendance at follow-up).^8^

 Although many countries acknowledge the severity of medical deserts and take action, they do so without a strong rationale underlying the choice of specific policies and other measures.^6,7^ As an example, in the OECD (Organisation for Economic Co-operation and Development) Health Systems Characteristics Survey of 2012 and 2016, countries reported which policies they had in place to address physician supply problems. Half of the countries indicated to use financial incentives to correct perceived geographical maldistribution, while it is known from the literature that financial incentives alone are unlikely to attract HWF to underserved areas and are more effective if combined with other types of measures.^9^

 Furthermore, when choosing a certain policy response or action, other contextual factors need to be taken into account. In the case of financial incentives, there are other reasons that should be considered that make physicians choose (not) to work in certain regions or possible legal barriers in place related to the choice of practice location.

 The purpose of this scoping review was to systematically map the research done in the area of HWF issues in medical deserts, to provide an overview of the different definitions, characteristics of medical deserts as well as the contributing factors and approaches to mitigate their HWF issues. Based on the information gathered from this review we will identify knowledge clusters and gaps for further research, which also will allow defining recommendations for all potential end users such as policy-makers and different stakeholders involved in HWF issues in medical deserts.

 This work was conducted as part of the “ROUTE-HWF” (a Roadmap OUT of mEdical deserts into supportive Health WorkForce initiatives and policies) project, a European Union (EU)-funded project that aims to reduce disparities in population’s health within the EU by ensuring timely access to high-quality healthcare in all regions of the EU.

## Methods

 We registered the protocol of the scoping review prospectively in Open Science Framework on June 25, 2021 with doi:10.17605/OSF.IO/UEBXY and adhered to the Preferred Reporting Items for Systematic reviews and Meta-Analyses extension for Scoping Reviews (PRISMA-ScR) checklist^10^ for reporting (Supplementary file 1).

 We used Arksey and O’Malley^11^ five-stage framework for scoping reviews: defining the research question, identifying relevant studies, study selection, data charting and collation and summarizing the results.

###  Defining the Research Question

 The following research questions were formulated: (*i*) What are medical deserts, and what are their main characteristics? (*ii*) What are the factors that contribute to medical deserts and their HWF issues? and (*iii*) What are the approaches to mitigate them?

###  Identifying Relevant Studies

 To identify relevant published studies, we searched the following bibliographic databases from inception to June 2021: Embase, Medline, CINAHL, Web of Science Core Collection, Google Scholar, and the Cochrane Library. The search strategies were drafted by the author team and further refined by an experienced biomedical information specialist and through discussion within the members of the research team. We followed PRESS Peer Review of Electronic Search Strategies recommendations.^12^ The electronic search strategy for MEDLINE database is provided in Table 1.

**Table 1:** Search Strategy in MEDLINE (Ovid)

**Step. No.**	**Search Strategy in Medline**
1	Health Personnel/ OR (nurse-patient-ratio* OR ((health* OR dental* OR care* OR medical* OR hospital* OR nursing) ADJ3 (personnel* OR workforce* OR labor-force* OR labour-force* OR manpower* OR work-force* OR resource*)) OR ((nurs* OR physician) ADJ3 (shortage*))).ab,ti,kf.
2	Rural Health/ OR Rural Health Services/ OR Rural Population/ OR (island* OR villager* OR ((rural* OR countryside* OR village*) ADJ3 (health* OR care* OR setting* OR area* OR population* OR communit* OR dweller* OR people* OR resident* OR societ* OR worker* OR nurs*)) OR medical-desert* OR ((underserv* OR remote* OR isolated OR mountain* OR far*) ADJ3 (area* OR neighborhood* OR neighbourhood* OR district* OR province*))).ab,ti,kf.
3	(taxonomy OR taxonomic* OR indicator* OR definition* OR defining* OR classificat* OR index* OR indice* OR scalogram* OR Gini).ab,ti,kf.
4	1 and 2 and 3
5	(exp animal/) NOT (human/)
6	4 not 5
7	(news OR congres* OR abstract* OR book* OR chapter* OR dissertation abstract*).pt.
8	6 not 7

 The final search strategy as used for the electronic bibliographic databases can be found in Supplementary file 2.

 In the review we included qualitative, quantitative, and mixed methods primary research studies addressing medical deserts with a focus on the definition, characteristics, contributing factors and approaches to mitigate the HWF issues in medical deserts.

 Peer-reviewed journal papers were included if they were written in English or a language that one of the authors was proficient in, were situated in Europe, the United States, Canada, Australia or New Zealand. Excluded were case reports, editorials, and articles without details about methods and/or results (Table 2).

**Table 2:** Inclusion and Exclusion Criteria

**Inclusion Criteria**	**Exclusion Criteria**
Publication type: Original research ie, quantitative (observational and interventional), qualitative, and mixed methods studies.	Books, editorials, correspondences, case reports, expert opinions, review articles, duplicative reports, study protocols, conference proceedings with unpublished results, and ongoing studies.
Population: HWF medical deserts (eg, due to shortage of physicians/nurses).	Studies outside of this population.
Outcomes: Definition and characteristics, contributing factors and approaches to mitigate/eliminate medical deserts.	Studies outside of these outcomes.
Restrictions: Western countries (ie, EU, the United States, Australia, and New Zealand).	Low- and middle-income countries. Asia, Africa, and South America.
Restrictions: Languages of publication restricted to Croatian, Dutch, English, Finnish, French, German, Polish, Spanish, Romanian, and Russian.	Studies outside of these languages.

Abbreviations: EU, European Union; HWF, health workforce.

 Furthermore, we searched for potential eligible studies that were not captured by our electronic database searches by checking the reference lists of included studies, relevant reviews, and by carrying out a cited reference search (forward citation tracking of the most relevant papers). Studies were included according to the same criteria as those found in the search of electronic databases.

###  Study Selection 

 The final search results of the electronic databases were exported into Endnote^©^, and duplicates were removed by the biomedical information specialist. Document information was uploaded in Rayyan^©^ after the removal of the duplicates. Three reviewers (AIGG, LEF, and JB) independently screened the title and abstracts. Disagreement among the three reviewers were resolved by consensus and discussion. To increase consistency, a calibration exercise of 50 studies was performed with the aim of achieving 80% of agreement between the three reviewers.^13^ The inclusion and exclusion criteria were reviewed during the calibration period. Two reviewers (AIGG and LEF) independently screened the full texts of the selected abstracts. Also, the full-texts disagreements on study selection were resolved by consensus and discussion. Furthermore, two reviewers (AIGG and LS) assessed the potential eligible studies gathered by other type of searches (eg, reference lists of already included studies) and included the ones that met the inclusion criteria.

###  Data Charting and Collation 

 A data charting form was jointly developed by two reviewers (AIGG and LEF) to determine which subjects and variables to extract. The two reviewers each charted half of the selected data. During the charting, the data form was updated in an iterative process between the two reviewers.

 We extracted data on article characteristics (eg, country of origin), type of HWF addressed (eg, general practitioner [GP]), type of medical desert (eg, island) and ‘outcome’ (ie, definition, characteristics, contributing factors, and approaches to mitigate HWF issues in medical deserts). The studies were grouped by the type of outcomes analyzed (ie, definition, characteristics, contributing factors of medical deserts and approaches to mitigate their HWF issues) and summarized by type of HWF and study design for each group, along with broad findings.

## Results

 The primary search produced over 2000 records. After removal of duplicates, 979 records were left for further assessment based on title and abstract resulting in 307 abstracts for retrieval of full texts. For 20 articles from the primary search, no full text could be retrieved. In total, 165 articles were excluded on the basis of the full-text, 105 reported about a population that was out of scope (eg, wrong country of origin), 59 did not meet the inclusion criteria, eight were not about medical deserts, seven were not about HWF and three were written in an excluded language. Supplementary file 3 presents all excluded studies and reasons for exclusion.

**Figure 1:**
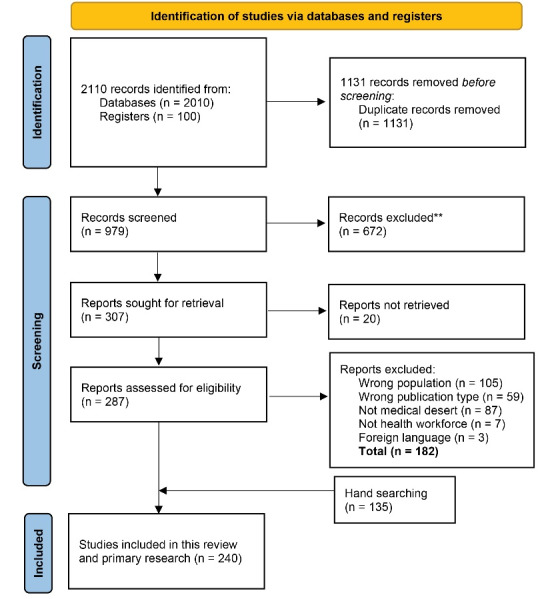
Flowchart of Review Search Strategy.

 One hundred and five studies were included in the scoping review after the electronic databases search and selection based on all inclusion and exclusion criteria (Figure 1). As 80% agreement between reviewers was achieved in the first calibration exercise, inclusion and exclusion criteria remained unchanged. Additionally, 135 studies were included after hand searching the reference lists of included studies and relevant reviews, and in addition by carrying out a cited reference search.

###  Key Characteristics of Included Studies

Table 3 and Supplementary file 4 show the key characteristics of the 240 articles that were finally included. Most were conducted in Australia and New Zealand (48%) and North America (44%). Eight percent of the articles were situated in Europe. Most studies were observational quantitative (80%) and focused on a variety of HWF groups, with a majority focusing on medical students and physicians (mostly GPs).

**Table 3:** Descriptive Summary of Included Studies (n = 240)

**Variable**	**Total, No. (%)**
**Study characteristics**	
Geographical location^*^	
North America	105 (44)
Europe	20 (8)
Australia and New Zealand	116 (48)
Design	
Observational, quantitative	193 (80)
Observational, qualitative	28 (12)
Observational, mixed methods	14 (6)
Quasi-experimental	5 (2)
Data collection method^*^	
Existing databases	96 (40)
Survey/questionnaire	126 (52)
Interviews	30 (13)
Focus group	3 (1)
**Participants’ characteristics**	
Type of HWF	
Medical students/other students	80 (33)
Physicians	85 (35)
Nurses	14 (6)
Allied HWF	23 (10)
Combination of HWF	27 (11)
Institutes/practices	11 (5)

Abbreviation: HWF, health workforce. *Studies may be included in more than one category.

**Figure 2:**
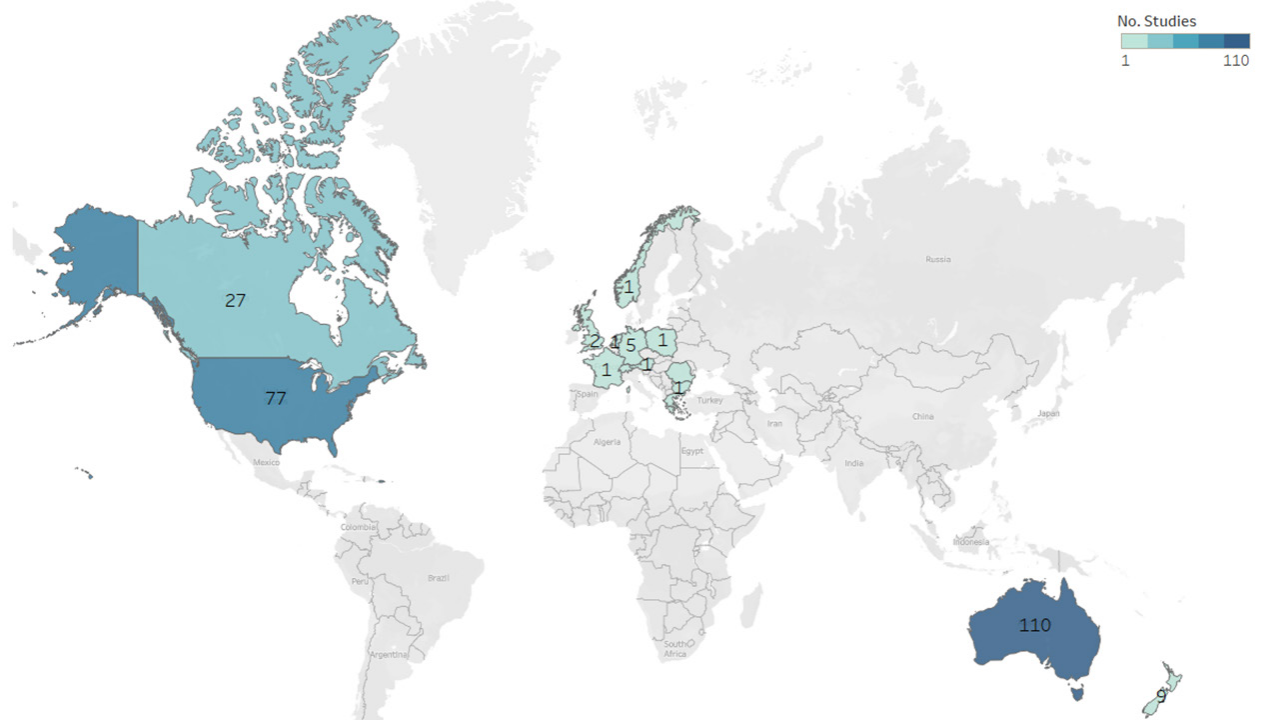
Number of Publications Per Country.

Figure 2 shows detailed information about the number of studies found per country.

 Of the included 240 articles, 71% (n = 171) referred, used or described a definition of medical desert, 40% (n = 95) described its characteristics, 47% (n = 112) described contributing or associated factors for medical deserts and 36% (n = 87) approaches to mitigate the HWF issues in medical deserts. These four themes will be further explored below.

###  Definition and Characteristics of Medical Deserts

 Most studies considered medical deserts as rural areas, underserved areas or used a measure of distance/time to a facility or a combination of the three.

 Of these, 69 studies did not define the term rural area.^14-83^ Fifty-eight reported a definition of rural area and used a single criterion to define it:

- the ratio between the population and the HWF,^84-93^
- the size of the population in an area,^43,94-129^
- the distance to the HWF,^130-136^
- the distance to the nearest town,^137^ or
- the number of hospital beds in the region.^138,139^

These criteria were mostly part of several more ‘formal’ definitions that were used by 78 studies as shown in Supplementary file 5. Twenty studies used a combination of factors or criteria to define rural areas.^36,140-158^ All definitions, except the Rural Ranking scale, were defined from the perspective of the population. The Rural Ranking scale is a criterion that defines medical deserts from the perspective of GPs (see Supplementary file 5).

 If we focus on the definitions from the perspective of the population, the following elements or criteria were identified:

- population size of the area,
- percentage of poverty in the area,
- percentage of population aged 65 and over,
- infant mortality rate in the area,
- mobility of the population in the area,
- health needs of the population in the area,
- number of HWF in the area,
- economic resources in the area,
- education and occupation options in the area,
- presence of a hospital or other health services in the area,
- population to provider ratio, and
- distance/time to facilities, distance/adjacent to metropolitan area.

These elements can be divided into four categories: (*i*) Size of the population, (*ii*) characteristics of the population, (*iii*) number of services in the area, and (*iv*) distance to services. Although all these elements seem relevant to define if an area is a potential medical desert, different definitions remain of how an ‘area’ is defined or demarcated as such. Therefore, it is not always possible to apply the definition elements presented above in countries if a different area definition is applied. For example, some studies defined areas as a community, county, province, mountain or island, while one study defined an area as a square kilometer.^120^ To define if an area is a medical desert, dividing a country in ‘blocks’ of a prespecified size might be an objective approach to compare countries on their medical desert areas and relevant criteria.

 For studies that based their definition of a medical desert on the perspective of GPs (ie, the Rural Ranking scale), the definition elements are shown in Supplementary file 5.^159^

###  Contributing Factors to Medical Deserts

 One hundred and twelve studies addressed contributing factors that may (have) enhanced or diminish medical deserts and their HWF issues. The factors extracted from the studies are presented below in four categories. Most factors were considered as both, positively as well as negatively influencing the workplace decision of HWF, depending on the individual preferences. Therefore, factors are described as influencing factors in general. In case that a factor was solely considered as positively or negatively influencing medical deserts, it is described as such within the results.

####  1. Work-Related Factors

 Fifty-five studies identified work-related factors that could contribute positively or negatively to medical deserts and their HWF issues:

- Low level of *job satisfaction*^24,27,31,50,59,102,160,161^ and *burnout* rates^27,31,53,102^ were found to be associated with working location, in favor of working in rural versus urban placements.
- Higher *workload* (eg, patient list, working hours, patient-related hours, on-call arrangements)^24,37,120^ in rural areas was reported by seventeen studies^24,27,31,35,37,41,52,59,70,118,120,137,162-166^ and considered a worrisome issue, and therefore contributing negatively to the willingness of working in rural areas.
- *Working conditions* such as working atmosphere,^34,40,46,50,84,161,164,166^ having a single hand or group practice^70,120,137,153^ or full-time versus part time job,^34,74,167^ can have a negative but as well a positive influence on the choice of working in a rural location, depending on personal preferences of the HWF.
- Furthermore, characteristics related to *the informal nature of rural practice* in general,^22,50,53,99,118,161,168^ travel hours,^50,74,83^ level of autonomy,^41,74,99,162^ and flexibility in practice structure^169^ were identified as factors influencing the decision to work in rural or urban areas, depending as well on personal preferences of the HWF.
- The *work variety* of rural practices, along with other factors such as *closer doctor-patient relationship*, good team collaboration, multidisciplinary and student experiences, positively influenced the attitude of the HWF towards working in rural areas. This relationship was found by 11 studies.^46,59,64,68,74,84,127,162,163,170,171^
- Five studies^40,59,74,169,172^ identified the lack of *personal recognition* and 22 *financial* issues such as lack of financial recognition, financial security, financial incentives and loan forgiveness^18,23,24,31,34,40,41,46,52,59,66,84,99,100,110,112,118,124,127,149,169,173^ when working in rural settings as a negative factor influencing the career choices of the HWF against working in rural.
- Lack of *career prospects*^23,44,53,74,99,118,174^ and *educational and professional development* opportunities,^74,83,84,110,118,124,163,164^ lack of professional support^14,27,31,44,74,81,163,164,168,169^ and *management support*^53,83,110,164^ and *professional isolation*^27,81,83,137,162^ as well as lack of access to *healthcare resources* (eg, equipment, personnel)^14,40,74,124,157^ were found as factors negatively associated with working in a rural setting.
- Furthermore, the lack of *availability of jobs,*^34,161^ the *length of employment* in the position (higher risk of turnover during the first six months),^99,167^ the lack of *intellectual challenge* (eg, scientific curiosity, complex care, research, procedural specialty),^50^ the willingness to get *professional specialization* education^141,175^ as well as *personal traits*^41,173^ also were found as factors that negatively influenced the choice of working in rural locations.

####  2. Lifestyle-Related Factors

 Thirty-three studies investigated lifestyle-related factors that may influence the recruitment and retention of HWF in medical deserts:

- Ten studies^31,41,53,68,83,112,127,161,162,168^ identified *rural lifestyle* in general as positively associated with the willingness to work in rural areas.
- *Work-life balance* was a positively influential factor described in three studies.^107,166,174^
- *Family issues* such as finding employment for the spouse or good children education were as well considered as very relevant factors in 17 studies^27,31,50,53,66,68,100,107,118,161–164,168,169,175,176^ diminishing the willingness to work in rural areas.
- *Feelings of isolation*,^27,70,162,166,170,177^ lack of access to other *desirable services* such as internet^170^ or *leisure activities*^46,83^ and *anonymity*^23,157,172^ were other relevant lifestyle-related factors described that influenced negatively the willingness to work in rural settings.
- Furthermore, *high costs of living* and *travelling* were considered as a significant incentive not to work and live in urban areas but in rural settings instead.^64,83,108^

####  3. Migration

 One study^148^ from Romania identified *migration* of the HWF to other countries as a contributing factor to medical deserts. This single outcome is probably specific for Romania, known as a typical ‘source country’ in cross-border HWF mobility like some other Eastern European countries.

####  4. Socio-demographics or Other HWF Characteristics

 Seventy-nine studies showed socio-demographic or other characteristics of the HWF that also may contribute to their career choices and subsequently influence HWF issues in medical deserts:

- *Age* was a factor described in nine studies^18,72,99,147,153,166,167,178,179^ influencing HWF turnover in rural areas; in some studies it was found that retirement due to aging of the HWF was not compensated by the inflow of health workers of younger age, because of their preference to work in urban placements.
- Both *male*^126,153^ and *female*^104,147,149,180,181^ (from 2014 onwards) health workers appear to be more willing to work in rural practice depending on the year of publication of the studies. Gender apparently has mixed effects on this career choice and therefore on HWF issues in medical deserts.
- Forty-five studies described^18,22,29,30,46,52,54,64,69,71,72,75,95,103,104,106,113,118,121,122,124,126,127,150,161,173,176,177,181-195^ *rural background* as a positive factor associated with working in a rural setting.^30,182,183,192-194,196^
- Forty-seven studies described *rural training* as a factor positively associated with working in rural areas.^18^, ^22,29,33,34,43,52,54,56,57,64,68,69,75,95,103,104,106,111,113,121,122,127,136,147,153,156,161,168,170,175,176,182,187-190,192,193,195-201^ One study though, showing controversial results.^95^
- Furthermore, having a lower *socio-economic status*^139,180,181^ or *educational level,*^157^ having a high medical school *admission score,*^150^ belonging to a *minority*group,^72,147,149,157,191,196^ having general *interest in rural practice*^16,103,176,185,189,191,202^ or specific professional *interest in primary care,*^173,176,180,181,189,190,200,203^ and getting *financial support* (ie, scholarships or funding)^30,64,184,190,191,200^ were all positively associated with working in a rural setting. Besides, *rural familiarity*^23,68,118,126^ and certain *character traits* (eg, altruism, self-confidence, curiosity, loyalty)^27,46,68,107,127,150,168^ were also found to positively influence the choice of working in rural practice.
- Finally, one study found that having a *non-English speaking background,* when working in English-speaking countries, was negatively associated with taking up rural practice.^139^

**Figure 3:**
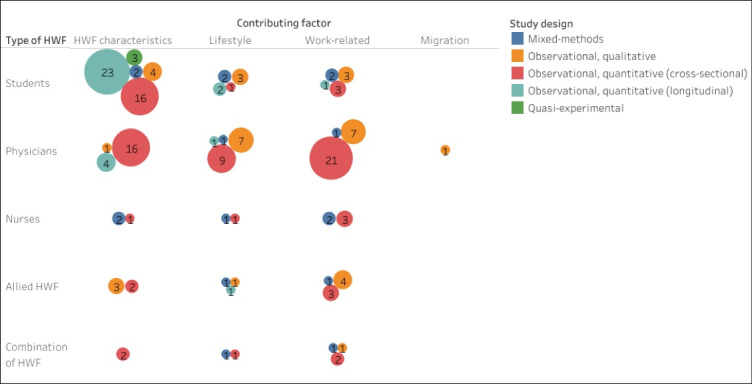
Types of Contributing Factors by Type of Health Work Force and Study Design. Abbreviation: HWF, health workforce.

The above mentioned four categories of factors (columns) involved in HWF issues in medical deserts (in most cases being defined as rural areas), are summarized in Figure 3. Here the size of the bubbles indicates the number of studies reporting contributing factors, broken down by type of HWF (rows), and study design (by color). Characteristics of the HWF were found by the largest number of studies as contributing factors, next to work-related and lifestyle factors. Migration as a contributing factor was found by only one study. In addition, our extraction shows that the studies vary by the type of HWF that was subject of the study, as well as the type of study design.

###  Approaches to Mitigate Medical Deserts

 We found eighty-three studies that described approaches to mitigate HWF issues in medical deserts. Comparable to our previous analysis, we present the approaches found in five categories.

####  1. Undergraduate Training Focused on Increased Rural Exposure

 The following approaches were found in the studies that can be classified under this category:

- Seven studies^42,55,93,197,201,204,205^ assessed approaches that are executed by *university-based rural clinical schools* which emphasize rural recruitment. Results from these studies showed that such approach had a positive effect regarding the number of graduates that stay near or in the same rural areas where they attended undergraduate training.
- Twenty-two studies^19,20,29,38,43,49,56,63,67,76,125,136,140,142,156,199,205-211^ assessed the effect of *rural clinical rotations* (eg, rural internships, rural immersion programs) during undergraduate training on the intentions to work in rural settings or actual recruitment and retention rates of the rural HWF. These studies can be broken down in two subcategories:
o Twelve studies^19,20,29,38,49,67,75,76,140,206,208,209^ evaluated the effect of such programs on the intention of students to practice in rural areas. Eight studies^19,20,29,38,67,140,206,209^ showed that an extended rural placement influenced medical and health science graduates’ intentions towards a rural career. The longer the clinical rotation, the more effective this was to increase the eventual choice of workplace location and future interest in a rural career.^19,209^ Williamson et al^76^ reported that the positive effects of a seven-week rural undergraduate placement on the attitudes towards rural health workplaces persisted in postgraduate years; but also found that the undergraduate training itself is unlikely to result in a significant effect. Furthermore, Orpin and Gabriel^49^ identified that the ‘rural exposure’ had actually influenced two-thirds of health science students away from a rural career. Two studies^75,208^ evaluated specific rural clinical-rotation programs: the so-called John Flynn Placement Program and the fifth-year rural health curriculum at Dunedin School of Medicine. Both appeared to have a positive influence on students’ intentions to enter and work in rural areas.
o Twenty studies^16,43,56,63,78,121,125,136,156,193,199,202,205,207,210-215^ assessed the recruitment and retention rates of graduates in rural areas after undergoing rural clinical rotations. Nineteen studies^16,43,56,63,78,121,136,156,193,199,202,205,207,210-215^ showed an increase in the number of graduates choosing rural placements associated with undergraduate rural clinical rotations. However, Butler and Sheppard^125^ reported that the undergraduate program was only adequate to prepare physiotherapy students for their professional roles but did not necessarily encourage students to accept rural positions.
- Four studies^96,115,214,216^ evaluated the effectiveness of *rural student recruitment* programs to increase the low number of rural students enrolled into medical faculties. They concluded that a program that enrolls students interested in rural healthcare areas, and provides training in rural communities, were successful to stimulate graduates to practice in rural areas.
- Ten studies assessed the effect of school programs *supporting early-entry rural and generalist pathways*^57,82,122,190,193,217-221^ on rural recruitment and retention. Wood^122^ showed that nursing students who attended a nursing program focusing on ‘rural nursing’ were twice as likely to practice in rural areas. Two studies^193,217^ presented that early career practice locations and movements of medical graduates from different rural clinical training programs positively influenced the likelihood to choose rural career paths. Six studies^57,190,218-221^ evaluated the Physician Shortage Area Program as an educational approach focusing on recruitment and retention of rural GPs demonstrating its success. Longenecker et al^222^ showed that medical school characteristics and activities may result in more graduates choosing rural general practice. Finally, Bennett et al^82^ described a structured and comprehensive educational clinical placement experience on undergraduate nurses. The authors showed that this enhanced the level of confidence of these nurses in the area of primary care.

####  2. Postgraduate Training and Continuing Medical Education Adapted to the Scope of Rural Practice

 For this category the following approaches were found in the studies:

- Postgraduate training as a *family or GP* has been associated with an increase in the likelihood of working in urban underserved and rural areas, in contrast with other specialists working in primary care.^116,223^ Another study showed that training in community health centers not only meets the HWF needs in rural areas, but also enhances the recruitment of GPs in underserved settings.^45^ Furthermore, exposing family practice residents to rural family practice training has shown to increase the number of GPs working in these rural areas.^17,61,115,142,214^
- Six studies^58,114,178,224-226^ assessed the need of *continuing education* strategies with the aim of developing procedural and non-procedural skills specific to rural practice. Hajat et al^224^ and Rourke et al^61^ identified job-specific continuing education as the most important training needs of rural local public health agencies and GPs, respectively. Two studies^32,60^ evaluated the development of an interdisciplinary palliative care education program. Both showed that the program increased the capacity to deliver palliative care as reported by rural and remote communities as well as the job satisfaction of the healthcare workers.
- *Online courses*were identified as the preferred means for receiving continuing education by nurses in rural schools.^58^ In Newman et al^47^ survey findings showed that videoconferencing was an overall success with general positive feedback of nurses working in rural areas. Ray et al^227^ evaluated the educational impact of videoconferencing to increase the confidence of healthcare workers to deliver quality palliative care in rural and remote areas. Results showed that the confidence level indeed increased significantly for all the types of HWFs.
- In three studies,*rural mentoring* was considered central to recruitment and retention of allied HWFs in rural areas.^65,114,169^
- Furthermore, *scholarships* to follow management of education programs in rural settings showed to have significantly increased rural nurses’ intention to stay in their current rural positions.^77^

####  3. Professional Support and Infrastructure

 This category of approaches was found in eight studies^39,61,73,99,141,225,228,229^ describing or assessing approaches to support rural HWF and provide them with improved infrastructure.

- Jones et al^225^ examined the effectiveness of a set of recruitment and retention *incentives*from the perspective of rural GPs. The GPs were asked to rate the importance of such approaches in terms of their impact. The two strategies that were rated most important were (1) better remuneration and (2) better after hours and on-call arrangements. Better locum availability and funding to improve practice infrastructure were rated as medium importance. Better education and professional support activity were rated as the least important. In Rourke et al,^61^ a different outcome of a similar study was presented. GPs working in rural areas rated funding for learner-driven continuing medical education as one of the most important solutions, along with reducing the number of on-call duty nights. Pathman et al^141^ compared the retention rates of a rural national scholarship program with other rural programs, showing that the effect of the scholarship program on retention rate of physicians was poor.
- In Kuhn et al,^229^ almost three quarters of local politicians agreed that one of the strategies that might improve primary care is the availability of allied health professional services. Lin and Goodale^39^ also showed that allied health professional services increased satisfaction among the HWF in rural areas.
- Humphreys et al^228^ defined *six sentinel indicators* as the best way to support recruitment and retention of GPs in rural areas (ie, total hours, public hospital, on-call, time-off, partner employment and schooling). Their study was based on a data collected in four population size groups and comparing five levels of rural areas.
- Hanson et al reported that having relatively perceived *autonomy* within their professional work settings^99^ has been a satisfactory approach for retaining nurses in rural areas.
- White et al^73^ developed and implemented a *stress management* and reduction program among healthcare workers in rural areas. Participants that used such intervention reported between 25% to 72% reduced stress levels.

####  4. Planning and Monitoring the HWF Distribution

 Three studies^86,158,226^ focused on strategies to better plan and monitor the HWF maldistribution in rural and underserved areas. The approach proposed by Bowman^86^ and McGrail et al^158^ is *to align general practice training distribution* to meet the needs of rural and underserved communities. Russell et al^226^ identified *benchmarks* to analyze the length of stay of primary care HWF in rural and remote areas, by using survival analysis of longitudinal data on healthcare workers to inform rural HWF planning and retention strategies.

####  5. Innovative Models of Care

 This final category consists of nine studies^25,28,36,62,87,123,138,229,230^ that described and/or evaluated innovative models of care as a solution to mitigate HWF issues in medical deserts. These approaches can be distinguished as follows:

- Four studies^25,28,62,230^ evaluated approaches that substituted in-person consultations of specialized HWF by using *telemedicine* in underserved rural areas. Such approaches comprised a ward-based geriatric consultation service delivered via a mobile videoconferencing system which showed to be highly accepted by patients and cost-effective.^25^ Also, a pediatric critical care telemedicine consultation was found to improve patient care^28^ and a program which placed telemonitors in rural satellite clinics to increase access to a pediatric obesity clinic which improved weight status compared with conventional treatment.^230^ A similar study was on a tele-oncology model of care which allowed cancer patients to receive specialist consultations and chemotherapy treatments closer to home.^62^
- Wood et al^123^ evaluated in a quasi-experimental study the implementation of a *satellite specialized HIV clinic* program which showed improved patient-related outcomes and increased access to best practice HIV care.
- Two studies^36,87^ assessed *interprofessional student-run clinics* providing care to vulnerable and underserved populations. Bradley et al^87^ demonstrated, through a three-year evaluation, substantial improvement of health-related outcomes as well as reduction of use of health resources such as number of emergency department visits and hospital admissions. Lawrence et al^36^ reported in a quasi-experimental study high levels of patients satisfaction.
- Ceronsky et al^138^ described the framework of a *rural palliative care* initiative consisting of individualized action plans tailored to the community’s needs and resources and verified its feasibility. They formulated five recommendations and conditions to support rural palliative care development: (1) external resources and support, (2) networking, (3) defining community-based metrics, (4) reimbursement for palliative care services, and (5) alignment of the palliative care program with other efforts to redesign care delivery.
- Kuhn et al^229^ analyzed different innovative models of care which can improve local primary care by discussing these with respondents from a local government point of view. Half of the respondents supported the implementation of patient buses as model (where patients come to the physician’s office), while less than one-third voted for mobile physician’s offices (where physicians or allied health workers go near the patient’ home). Telemedicine, which allows both the patient and the HWF to stay at home or office, respectively, appeared to be a model that was seen less suitable by the local politicians.

**Figure 4:**
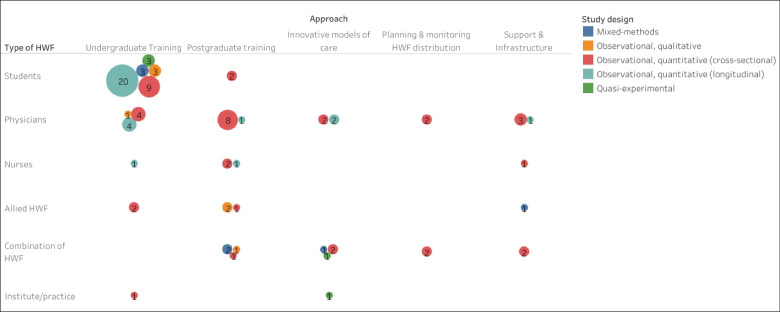
Types of Approaches by Type of Health Work Force and Study Design. Abbreviation: HWF, health workforce.

The above list and categories of approaches to mitigate HWF issues in medical deserts (in most cases being defined as rural areas), is summarized in Figure 4. Here the size of the circles shows the number of studies, broken down by type of approach (columns), type of HWF (rows), and study design (by color). The figure makes clear that most studies described approaches with regard to undergraduate and postgraduate training, either directed to medical or nurse students, or physicians working in rural areas.

## Discussion

 This paper provides a systematic overview of primary research published in scientific journals on the definitions and characteristics of medical deserts, and the contributing factors and approaches to mitigate the HWF issues in medical deserts with a focus on Western countries.

###  Summary of Results and Comparison With Literature

 This scoping review included a significant body of 240 empirical studies relevant to the subject of medical deserts in the context of their HWF issues. Most of the peer-reviewed articles found were from Australia, New Zealand and North America (92%). On the one hand this reflects the bias of Anglo-Saxon publications often seen in literature reviews.^9,231^ On the other, it also reflects the size and maturity of the challenge related to medical deserts in such large countries and geographical settings. We also found a number of studies on medical deserts in European countries such as Germany and Greece, among others. Most of the studies found used an observational study design (98%), a few used an observational longitudinal design (22%) and just a small percentage used a quasi-experimental design (2%), as it has as well been reported in other reviews.^9,232,233^

 Most medical deserts were defined in the studies by population-based characteristics, ie, population density which is mostly referred to as rural areas.^43,94-106,108,110-116,118-129,228,234^ However, a relevant number of studies referred to rural areas without a proper definition and furthermore without a consistent definition of what an area was considered defaulting the comparison among them or the generalizability of the results.

 The contributing factors that enhance or sustain HWF issues in medical deserts seem to mainly depend on the background and previous job characteristics of the HWF, followed by work-related and lifestyle related factors but to a lesser extent. Without claiming this as the ultimate study, we refer to Godwin et al^235^ as an example. Their systematic review with a focus on dental practitioners working in rural areas showed that the most mentioned motivational factor for recruitment and retention was the effect of prior ‘rural exposure’ for dental practitioners. The study showed that having a rural background (39%) and having received rural training were the most frequently (42%) and positively associated factors with working in a rural setting. These findings have been corroborated in a review of reviews performed by Asghari et al^231^ about the most influential factors for recruitment and retention of GPs.

 Finally, the studies included in our review show that approaches and strategies to mitigate HWF issues in medical deserts mostly focused on training; and thereby the early recruitment and exposure of students and HWF to rural areas. Next improving the scope of rural practice was also frequently found as an effective approach as also shown in one recently published review of the literature.^236^ Verma et al^233^ confirmed in their systematic review that although the evidence base for recruitment strategies was weak, they found evidence to support undergraduate and postgraduate placements in medical deserts. Buykx et al^237^ found that as multiple factors influence recruitment and retention of the HWF, a flexible and multifaceted response is needed. According to Dolea et al^232^ there is frequently a lack of coherence between the proposed strategy for recruitment and retention of the HWF and the factors that matter most to health workers in their choice of practice location. Therefore, a situation analysis should be mandatory before selecting the most appropriate approach or approaches to encourage the HWF to choose and stay in a medical desert.

###  Strengths and Limitations

 To the best of our knowledge, our study provides the first scoping review of empirical studies on (1) the definitions and characteristics of medical deserts, and (2) the contributing factors and approaches to mitigate HWF issues in medical deserts in Western countries. Our study has also contributed to a categorization of studies on medical deserts, enabling further analyses of the relationship between different types of medical deserts, types of HWF groups and issues, and the related contributing factors and potential solutions. Our review also provides a base and an agenda for further research in this field. We found that observational studies were the most common type of design of the studies included, which shows the scarcity of longitudinal studies that actually investigate the impact of factors contributing to HWF issues in medical deserts. Also, we identified the absence of interventional studies to evaluate the effectiveness of approaches to mitigate medical deserts. Therefore, longitudinal studies as well as controlled experimental studies should be increasingly encouraged and funded. We also identified that the majority of the studies focused on medical students or physicians (mostly GPs) and more efforts should be made to determine the factors and evaluate programs targeted at other types of health workers.

 A limitation of this study is that we did not include ‘grey literature’ that might have been published in non-scientific journals, national, regional or sector-specific sources. We explored this type literature but it has been found not to identify additional studies in a way that justifies the effort involved in this type of search.^238^ We also excluded studies from lower-middle income countries and therefore, results can only be generalized to high income countries from Australia and Zealand, North America and (to a lesser extent) Europe. Additionally, we did not use specific related terms in our search to identify studies performed in European countries and thus to increase the sensitivity of the search to the detriment of specificity, which may have caused a loss of studies focused on Europe. Contributing factors to medical deserts and approaches to mitigate them may not be comparable across continents, as may not be across countries, and therefore not generalizable.

## Conclusions

 This scoping review has collected, classified, extracted and synthesized the available empirical studies related to medical deserts and their HWF issues, published until June 2021 in Western countries. Whilst most studies originate from Australia, New Zealand and North America, studies from European countries were also included. Next to descriptive results we identified several gaps in the set of 240 studies included. One is the omission of longitudinal studies that measure the actual impact of factors contributing to HWF issues in medical deserts. Related is the lack of interventional studies that evaluate the effectiveness of approaches to mitigate HWF issues in medical deserts. We therefore advocate that more and next studies should invest in larger scaled and more rigor research, to fulfill the need for more evidence and research-based policy in medical deserts. This review shows that this research can be well based on the current studies, as new studies are now required to enable best practice outcomes for HWF policies in medical deserts.

## Acknowledgements

 The authors wish to thank Elise Krabbendam from the Erasmus MC Medical Library for developing and updating the search strategies.

## Ethical issues

 Not applicable.

## Competing interests

 Authors declare that they have no competing interests.

## Disclaimer

 The views expressed in this paper represent the opinions of the authors and not an official position of the institutions of their affiliations.

## Funding

 This work was co-funded by the European Union’s Health Programme (2014-2020) under grant agreement no. 101018379 - ROUTE-HWF. The publication is co-funded by the Polish Ministry of Education and Science within the project “PMW” in the years 2021-2024; agreement no. 5176/HP3/2021/2.

## Supplementary files


Supplementary file 1. PRISMA-ScR Checklist.

Supplementary file 2. Search Strategies by Electronic Databases.

Supplementary file 3. Excluded Studies and Reasons for Exclusion.

Supplementary file 4. Key Characteristics of the Included Studies.

Supplementary file 5. Definitions of Medical Deserts.
