# Supplementary material for: Characteristics of Medical Deserts and Approaches to Mitigate Their Health Workforce Issues: A Scoping Review of Empirical Studies in Western Countries
Source: Int J Health Policy Manag. 2023 Aug 15;12:7454. doi: 10.34172/ijhpm.2023.7454 (PMC10590222; doi:10.34172/ijhpm.2023.7454)
Supplement: Supplementary file 2 — Search Strategies by Electronic Databases. [file ijhpm-12-7454-s002.pdf]

Article title: Characteristics of Medical Deserts and Approaches to Mitigate Their Health Workforce Issues: A Scoping Review of Empirical Studies in Western Countries

Journal name: International Journal of Health Policy and Management (IJHPM)

Authors' information: Linda E. Flinterman[1]*¶, Ana I. González-González[2]¶, Laura Seils[2], Julia Bes[1], Marta Ballester[2], Joaquim Bañeres[2], Sorin Dan[3], Alicja Domagala[4], Katarzyna Dubas-Jakóbczyk[5], Robert Likic[6], Marieke Kroezen[7], Ronald Batenburg[1,8]

[1]Health Workforce and Organization Studies, Netherlands Institute for Health Services Research (NIVEL), Utrecht, The Netherlands.

[2]Avedis Donabedian Research Institute – UAB, Madrid, Spain.

[3]Innovation and Entrepreneurship InnoLab, University of Vaasa, Vaasa, Finland.

[4]Department of Health Policy and Management, Institute of Public Health, Jagiellonian University, Krakow, Poland.

[5]Department of Health Economics and Social Security, Institute of Public Health, Jagiellonian University, Krakow, Poland.

[6]School of Medicine, University of Zagreb, Zagreb, Croatia.

[7]Trimbos Institute, Netherlands Institute of Mental Health and Addiction, Utrecht, The Netherlands.

[8]Department of Sociology, Radboud University, Nijmegen, The Netherlands.

¶ Both authors contributed equally to this paper.

(*Corresponding author: Email: l.flinterman@nivel.nl)

Citation: Flinterman LE, González-González AI, Seils L, et al. Characteristics of medical deserts and approaches to mitigate their health workforce issues: a scoping review of empirical studies in Western countries. Int J Health Policy Manag. 2023;12:7454. doi:10.34172/ijhpm.2023.7454

Supplementary file 2. Search Strategies by Electronic Databases

- 06.05.2021

Medline [medall] via Ovid

1	Health Personnel/ OR (nurse-patient-ratio* OR ((health* OR dental* OR care* OR medical* OR hospital* OR nursing) ADJ3 (personnel* OR workforce* OR labor-force* OR labour-force* OR manpower* OR work-force* OR resource*)) OR ((nurs* OR physician) ADJ3 (shortage*))).ab,ti,kf.	
2	Rural Health/ OR Rural Health Services/ OR Rural Population/ OR (island* OR villager* OR ((rural* OR countryside* OR village*) ADJ3 (health* OR care* OR setting* OR area* OR population* OR communit* OR dweller* OR people* OR resident* OR societ* OR worker* OR nurs*)) OR medical-desert* OR ((underserv* OR remote* OR isolated OR mountain* OR far*) ADJ3 (area* OR neighborhood* OR neighbourhood* OR district* OR province*))).ab,ti,kf.	
3	(taxonomy OR taxonomic* OR indicator* OR definition* OR defining* OR classificat* OR index* OR indice* OR scalogram* OR Gini).ab,ti,kf.	
4	1 and 2 and 3	
5	(exp animal/) NOT (human/)	
6	4 not 5	
7	(news OR congres* OR abstract* OR book* OR chapter* OR dissertation abstract*).pt.	
8	6 not 7	665

Embase

('health care personnel management'/exp OR (nurse-patient-ratio* OR ((health* OR dental* OR care* OR medical* OR hospital* OR nursing) NEAR/3 (personnel* OR workforce* OR labor-force* OR labour-force* OR manpower* OR work-force* OR resource*)) OR ((nurs* OR physician) NEAR/3 (shortage*))):ab,ti,kw) **AND** ('rural health'/de OR 'rural health care'/exp OR 'rural population'/de OR (island* OR villager* OR ((rural* OR countryside* OR village*) NEAR/3 (health* OR care* OR setting* OR area* OR population* OR communit* OR dweller* OR people* OR resident* OR societ* OR worker* OR nurs*)) OR medical-desert* OR ((underserv* OR remote* OR isolated OR mountain* OR far*) NEAR/3 (area* OR neighborhood* OR neighbourhood* OR district* OR province*))):ab,ti,kw) **AND** ('taxonomy'/de OR (taxonomy OR taxonomic* OR indicator* OR definition* OR defining* OR classificat* OR index* OR indice* OR scalogram* OR Gini):ab,ti,kw) NOT ((animal/exp OR animal*:de OR nonhuman/de) NOT ('human'/exp)) NOT ([Conference Abstract]/lim)

Web of Science

TS=(((nurse-patient-ratio* OR ((health* OR dental* OR care* OR medical* OR hospital* OR nursing) NEAR/2 (personnel* OR workforce* OR labor-force* OR labour-force* OR manpower* OR work-force* OR resource*)) OR ((nurs* OR physician) NEAR/2 (shortage*)))) AND ((island* OR villager* OR ((rural* OR countryside* OR village*) NEAR/2 (health* OR care* OR setting* OR area* OR population* OR communit* OR dweller* OR people* OR resident* OR societ* OR worker* OR nurs*)) OR medical-desert* OR ((underserv* OR remote* OR isolated OR mountain* OR far*) NEAR/2 (area* OR neighborhood* OR neighbourhood* OR district* OR province*)))) AND ((taxonomy OR taxonomic* OR indicator* OR definition* OR defining* OR classificat* OR index* OR indice* OR scalogram* OR Gini)) NOT ((animal* OR rat OR rats OR mouse OR mice OR murine OR dog OR dogs OR canine OR cat OR cats OR feline OR rabbit OR cow OR cows OR bovine OR rodent* OR sheep OR ovine OR pig OR swine OR porcine OR veterinar* OR chick* OR zebrafish* OR baboon* OR nonhuman* OR primate* OR cattle* OR goose OR geese OR duck OR macaque* OR avian* OR bird* OR fish*) NOT (human* OR patient* OR women OR woman OR men OR man))) AND DT=(Article OR Review OR Letter OR Early Access)

Cinahl – 269 refs

(MH Personnel Management OR TI((nurse-patient-ratio* OR ((health* OR dental* OR care* OR medical* OR hospital* OR nursing) N2 (personnel* OR workforce* OR labor-force* OR labour-force* OR manpower* OR work-force* OR resource*)) OR ((nurs* OR physician) N2 (shortage*)))) OR AB((nurse-patient-ratio* OR ((health* OR dental* OR care* OR medical* OR hospital* OR nursing) N2 (personnel* OR workforce* OR labor-force* OR labour-force* OR manpower* OR work-force* OR resource*)) OR ((nurs* OR physician) N2 (shortage*))))) AND (MH Rural Health OR MH Rural Health Personnel OR MH Rural Health Services OR MH Rural Health Centers OR MH Hospitals, Rural+ OR MH Rural Population+ OR TI((island* OR villager* OR ((rural* OR countryside* OR village*) N2 (health* OR care* OR setting* OR area* OR population* OR communit* OR dweller* OR people* OR resident* OR societ* OR worker* OR nurs*)) OR medical-desert* OR ((underserv* OR remote* OR isolated OR mountain* OR far*) N2 (area* OR neighborhood* OR neighbourhood* OR district* OR province*)))) OR AB((island* OR villager* OR ((rural* OR countryside* OR village*) N2 (health* OR care* OR setting* OR area* OR population* OR communit* OR dweller* OR people* OR resident* OR societ* OR worker* OR nurs*)) OR medical-desert* OR ((underserv* OR remote* OR isolated OR mountain* OR far*) N2 (area* OR neighborhood* OR neighbourhood* OR district* OR province*))))) AND (TI((taxonomy OR taxonomic* OR indicator* OR definition* OR defining* OR classificat* OR index* OR indice* OR scalogram* OR Gini)) OR AB((taxonomy OR taxonomic* OR indicator* OR definition* OR defining* OR classificat* OR index* OR indice* OR scalogram* OR Gini)))

Cochrane – 63 refs

(((nurse NEXT/1 patient-ratio*) OR ((health* OR dental* OR care* OR medical* OR hospital* OR nursing) NEAR/3 (personnel* OR workforce* OR (labor NEXT/1 force*) OR (labour NEXT/1 force*) OR manpower* OR (work NEXT/1 force*) OR resource*)) OR ((nurs* OR physician) NEAR/3 (shortage*))):ab,ti,kw) **AND** ((island* OR villager* OR ((rural* OR countryside* OR village*) NEAR/3 (health* OR care* OR setting* OR area* OR population* OR communit* OR dweller* OR people* OR resident* OR societ* OR worker* OR nurs*)) OR (medical NEXT/1 desert*) OR ((underserv* OR remote* OR isolated OR mountain* OR far*) NEAR/3 (area* OR neighborhood* OR neighbourhood* OR district* OR province*))):ab,ti,kw) **AND** ((taxonomy OR taxonomic* OR indicator* OR definition* OR defining* OR classificat* OR index* OR indice* OR scalogram* OR Gini):ab,ti,kw)

Google Scholar – 100 refs
"health|care|medical personnel|workforce|"labor*force"|"labour*force"|manpower|"work*force"|resources" "rural health|care|area"|"medical desert" taxonomy|taxonomies
