# Supplementary material for: Characteristics of Medical Deserts and Approaches to Mitigate Their Health Workforce Issues: A Scoping Review of Empirical Studies in Western Countries
Source: Int J Health Policy Manag. 2023 Aug 15;12:7454. doi: 10.34172/ijhpm.2023.7454 (PMC10590222; doi:10.34172/ijhpm.2023.7454)
Supplement: Supplementary file 3 — Excluded Studies and Reasons for Exclusion. [file ijhpm-12-7454-s003.pdf]

Article title: Characteristics of Medical Deserts and Approaches to Mitigate Their Health Workforce Issues: A Scoping Review of Empirical Studies in Western Countries

Journal name: International Journal of Health Policy and Management (IJHPM)

Authors' information: Linda E. Flinterman[1]*¶, Ana I. González-González[2]¶, Laura Seils[2], Julia Bes[1], Marta Ballester[2], Joaquim Bañeres[2], Sorin Dan[3], Alicja Domagala[4], Katarzyna Dubas-Jakóbczyk[5], Robert Likic[6], Marieke Kroezen[7], Ronald Batenburg[1,8]

[1]Health Workforce and Organization Studies, Netherlands Institute for Health Services Research (NIVEL), Utrecht, The Netherlands.

[2]Avedis Donabedian Research Institute – UAB, Madrid, Spain.

[3]Innovation and Entrepreneurship InnoLab, University of Vaasa, Vaasa, Finland.

[4]Department of Health Policy and Management, Institute of Public Health, Jagiellonian University, Krakow, Poland.

[5]Department of Health Economics and Social Security, Institute of Public Health, Jagiellonian University, Krakow, Poland.

[6]School of Medicine, University of Zagreb, Zagreb, Croatia.

[7]Trimbos Institute, Netherlands Institute of Mental Health and Addiction, Utrecht, The Netherlands.

[8]Department of Sociology, Radboud University, Nijmegen, The Netherlands.

¶ Both authors contributed equally to this paper.

(*Corresponding author: Email: l.flinterman@nivel.nl)

Citation: Flinterman LE, González-González AI, Seils L, et al. Characteristics of medical deserts and approaches to mitigate their health workforce issues: a scoping review of empirical studies in Western countries. Int J Health Policy Manag. 2023;12:7454. doi:10.34172/ijhpm.2023.7454

Supplementary file 3. Excluded Studies and Reasons for Exclusion

Number	1st author	year	title	Reason for exclusion
1	Al Saffer, Q.	2021	The capacity of primary health care facilities in Saudi Arabia: infrastructure, services, drug availability, and human resources	wrong population
2	Alameddine, M.	2016	Upscaling the recruitment and retention of human resources for health at primary healthcare centres in Lebanon: a qualitative study	wrong population
3	Alvi, R.	2011	Experience of developing rural surgical care in a remote mountainous region of Pakistan: Challenges and opportunities	wrong population
4	Ament, L. A.	1991	The integration of a QA/RM program in a rural healthcare obstetrical service	wrong publication type
5	Averill, J.B.	2006	Getting started: initiating critical ethnography and community-based action research in a program of rural health studies	wrong publication type

Number	1st author	year	title	Reason for exclusion
6	Ayaz, A.	2014	Brain drain human resources and human capital growth in Pakistan: a political and economical perspective	wrong population
7	Badanta-Romero, B.	2021	Cuidados enfermeros para el abordaje de la salud comunitaria en poblaciÃ³n indÃgena de la Amazonia peruana	foreign language
8	Bahadori, M.	2012	Classification of health structural indicators using scalogram model in Golestan province, northern Iran	wrong population
9	Bamberg, R.	1994	The development of a state-level health manpower database using an employer-based survey: A pilot project	Not medical desert
10	Bärnighausen	2009	Financial incentives for return of service in underserved areas: A systematic review	wrong publication type
11	Barzola-Cordero, V.	2011	Implementation of medical specialists' brigades in the areas of Universal Health coverage: The Peruvian Ministry of Health experience, 2009-2010	wrong population
12	Batura, N.	2016	Is the Job Satisfaction Survey a good tool to measure job satisfaction amongst health workers in Nepal? Results of a validation analysis	wrong population
13	Benskin, L. L. L.	2012	A Concept Development of the Village Health Worker	wrong publication type
14	Berke, E. M.	2009	Practical and policy implications of using different rural-urban classification systems: A case study of inpatient service utilization among veteran administration users	wrong population
15	Bertrand , W. E.	1979	Attitudinal classification of health manpower in Colombia: A research note on urban/rural differences in occupational prestige	wrong population
16	Bhan, N.	2020	Access to women physicians and uptake of reproductive, maternal and child health services in India	wrong population
17	Brahmapurkar, K. P.	2018	The need to focus on medical education in rural districts of India	wrong population
18	Breindel, C. L.	1981	Marketing strategies in rural areas	wrong publication type
19	Brown, M. C.	1994	Using Gini-style indices to evaluate the spatial patterns of health practitioners: Theoretical considerations and an application based on Alberta data	wrong publication type
20	Brown, P.	2016	Place matters	wrong publication type
21	Bryant M.	1995	Measuring time utilization in rural health centres	wrong population
22	Burrows, G.	2019	What are the support needs of nurses providing emergency care in rural settings as reported in the literature? A scoping review	wrong publication type
23	Cancedda, C.	2014	Enhancing formal educational and in-service training programs in rural Rwanda: A partnership among the public sector, a nongovernmental organization, and Academia	wrong population
24	Carson, D. B.	2016	Recruitment and retention of professional labour: the health workforce at settlement level	wrong publication type
25	Casey, M. M	1997	Rural health network development: Public policy issues and state initiatives	wrong publication type
26	Celletti, F.	2010	Can the deployment of community health workers for the delivery of HIV services represent an effective and sustainable response to health workforce shortages? Results of a multicountry study	wrong population

Number	1st author	year	title	Reason for exclusion
27	Cheek, C.	2017	Building a local medical workforce in Tasmania: where are international fee-paying medical graduates likely to work?	wrong population
28	Cheek, C.	2019	Building a medical workforce in Tasmania: A profile of medical student intake	wrong population
29	Chen, F.	2015	Analysis of the equity of public health resources allocation in Chongqing from the perspective of migration	wrong population
30	Chowdhury, M.	1998	The role of traditional birth attendants in a safe delivery programme in Bangladesh	wrong population
31	Colagiuri, R.	2006	Building capacity to reduce diabetes complications in the Pacific: The Vanuatu experience so far	wrong population
32	Collins, C.	2016	Challenges of Recruitment and Retention in Rural Areas	wrong publication type
33	Cossman, J.	2017	The differential effects of rural health care access on race-specific mortality	not HWF
34	Curry, L. A.	2012	Community perspectives on roles and responsibilities for strengthening primary health care in rural Ethiopia	wrong population
35	Dayrit, M. M.	2011	Addressing the Human Resources for Health crisis in countries: How far have we gone? What can we expect to achieve by 2015?	wrong population
36	DeFriese, G. H.	1981	An agenda for health services research in primary care	wrong publication type
37	Deng, F.	2017	Expanding public health in China: an empirical analysis of healthcare inputs and outputs	wrong population
38	Dhingra, B.	2011	National rural health mission	wrong population
39	Dhungel, B.	1988	Planning for rural health services in Nepal	wrong population
40	Dodson, Z. M.	2017	How to allocate limited healthcare resources: Lessons from the introduction of antiretroviral therapy in rural Mozambique	wrong population
41	Dolea, C.	2010	Evaluated strategies to increase attraction and retention of health workers in remote and rural areas	wrong publication type
42	Dong, E.	2020	Differences in regional distribution and inequality in health-resource allocation at hospital and primary health centre levels: A longitudinal study in Shanghai, China	wrong population
43	Drennan, V. M.	2019	Global nurse shortages - The facts, the impact and action for change	wrong population
44	Duarte-Gómez, M. B.	2015	Social determinants of infant mortality in socioeconomic deprived rural areas in Mexico	wrong population
45	Eley, D. S.	2007	Will Australian rural clinical schools be an effective workforce strategy? Early indications of their positive effect on intern choice and rural career interest	wrong publication type
46	El-Jardali, F.	2013	A national study on nurses' retention in healthcare facilities in underserved areas in Lebanon	wrong population
47	Erchick, D. J.	2020	Feasibility of training community health workers to conduct periodontal examinations: a validation study in rural Nepal	wrong population
48	Erdenee, O.	2017	Distribution of health care resources in Mongolia using the Gini coefficient	wrong population

Number	1st author	year	title	Reason for exclusion
49	Erickson, J. S.	2011	Rural medical education in the WWAMI region: instituting a rural longitudinal medical school curriculum in association with a rural longitudinal integrated community clerkship experience	wrong publication type
50	Fang, P.	2010	Regional inequality in health and its determinants: Evidence from China	wrong population
51	Fang, P.	2014	Factors that influence the turnover intention of Chinese village doctors based on the investigation results of Xiangyang City in Hubei Province	wrong population
52	Farmer, J.	2015	A scoping review of the association between rural medical education and rural practice location	wrong publication type
53	Farmer, J. C.	2001	Rural deprivation: Reflecting reality	wrong publication type
54	Federal register	2002	Medicare program	wrong publication type
55	Francis, J. R.	2020	Disparity in distribution of inpatient hospital services in Australia	wrong publication type
56	Freeman, R.	2013	Dental therapists/hygienists working in remote-rural primary care: A structured review of effectiveness, efficiency, sustainability, acceptability and affordability	wrong publication type
57	Fujiwara, K.	2016	Trends for the Geographic Distribution of Radiological Resources in Hokkaido, Japan: Data Analysis Using Gini Coefficient and Herfindahl-Hirschman Index	wrong population
58	Gajewski, J.	2019	The contribution of non-physician clinicians to the provision of surgery in rural Zambia-a randomised controlled trial	wrong population
59	Ghosh, S.	1979	Primary health care for developing countries	wrong publication type
60	Gibson, O. R.	2013	A systematic review of evidence on the association between hospitalisation for chronic disease related ambulatory care sensitive conditions and primary health care resourcing	not HWF
61	Giovanella, L.	2017	Comprehensive primary care and segmented health systems in South America	wrong population
62	Giraldo Molina, C. I.	2000	Nursing care needs: criteria to define nursing personnel requirements	wrong publication type
63	Godwin, D.	2014	Dental practitioner rural work movements: a systematic review	wrong publication type
64	Greer, T. M.	2011	Addressing Disparities in Rural Health	wrong publication type
65	Greiner, D.S.	2008	Rural health nursing research review: global perspectives	wrong publication type
66	Grobler, L.	2015	Interventions for increasing the proportion of health professionals practising in rural and other underserved areas	wrong publication type
67	Gross, J. M.	2010	The impact of an emergency hiring plan on the shortage and distribution of nurses in Kenya: The importance of information systems	wrong population
68	Hagopian, A.	2012	Applying WHO's 'workforce indicators of staffing need' (WISN) method to calculate the health worker requirements for India's maternal and child health service guarantees in Orissa State	wrong population
69	Han, Y.	2012	Accessibility of primary health care workforce in rural China	wrong population

Number	1st author	year	title	Reason for exclusion
70	Hatam, N.	2015	Distribution of health resource allocation in the Fars province using the scalogram analysis technique in 2011	wrong population
71	Hays, R.	2017	Interpreting rural career intention in medical workforce research	wrong publication type
72	Hays, R. B.	2007	Rural medical education in Europe: the relevance of the Australian experience	wrong publication type
73	Hays, R. B.	1998	Methodological issues in medical workforce analysis: implications for regional Australia	wrong publication type
74	Hermawan, A.	2019	Health Workforce Distribution (Physicians, Nurses Midwives) Analysis in Indonesia 2013 by Gini Index	foreign language
75	Higgins-Steele, A.	2015	Peer-driven quality improvement among health workers and traditional birth attendants in Sierra Leone: linkages between providers' organizational skills and relationships	wrong population
76	Hsu, Y. E.	2016	Measuring inequality in physician distributions using spatially adjusted Gini coefficients	wrong population
77	Humphreys, J.	1999	Rural health status: what do statistics show that we don't already know?	wrong publication type
78	Hunter, D.	2009	Boundaries of the health workforce: definition and classification of health workers	wrong publication type
79	Irel , M	2016	Expanding the primary health care workforce through contracting with nongovernmental entities: the cases of Bahia and Rio de Janeiro	wrong population
80	Ishikawa, T.	2017	Forecasting the regional distribution and sufficiency of physicians in Japan with a coupled system dynamics-geographic information system model	wrong population
81	Judd, F. K. (	2001	Improving access for rural Australians to treatment for anxiety and depression: the University of Melbourne Depression and Anxiety Research and Treatment Group â€¦	wrong publication type
82	Kadam, S.	2016	A study of organizational versus individual needs related to recruitment, deployment and promotion of doctors working in the government health system in Odisha state, India	wrong population
83	Kashima, S.	2014	Non-physician communities in Japan: are they still disadvantaged?	wrong population
84	Kebriaei, A.	2009	Job satisfaction among community health workers in Zahedan District, Islamic Republic of Iran	wrong population
85	Kelman, I.	2020	Linking Disaster Risk Reduction and Healthcare in Locations with Limited Accessibility: Challenges and Opportunities of Participatory Research	not HWF
86	Kerr, L	2021	Rural emergency departments: a systematic review to develop a resource typology relevant to developed countries	wrong publication type
87	Knippenberg, R.	1997	Implementation of the Bamako initiative: Strategies in Benin and Guinea	wrong population
88	Kolehmainen-Aitken, R. L.	1990	'Indicators of staffing need': Assessing health staffing and equity in Papua New Guinea	wrong population
89	Leight, S.B.	2003	To describe the application of the vulnerable populations conceptual model to rural health	wrong publication type
90	Leonfanti, F. L.	1988	Neuquen, Argentina: Provincial health policies and their results	wrong population
91	Lin, S. W.	2015	New indices for home nursing care resource disparities in rural and urban areas, based on geocoding and geographic distance barriers: a cross-sectional study	wrong population

Number	1st author	year	title	Reason for exclusion
92	Liu, T.	2020	Regional Differences and Influencing Factors of Allocation Efficiency of Rural Public Health Resources in China	wrong population
93	Liu, W.	2019	Health expenditure efficiency in rural China using the super-SBM model and the Malmquist productivity index	wrong population
94	Lohmann, J.	2019	Psychological wellbeing in a resource-limited work environment: examining levels and determinants among health workers in rural Malawi	wrong population
95	Lourenco, A. E. P.	2012	The meaning of 'rural' in rural health: A review and case study from Brazil	wrong population
96	Lysdahl, K. B.	2007	Geographical variation in radiological services: a nationwide survey	not HWF
97	Martiniuk, A. L.	2020	Capability ... what's in a word? Rural Doctors Network of New South Wales Australia is shifting to focus on the capability of rural health professionals	wrong publication type
98	Matsumoto, M.	2013	Do rural and remote areas really have limited accessibility to health care? Geographic analysis of dialysis patients in Hiroshima, Japan	wrong population
99	Mbemba, G.	2013	Interventions for supporting nurse retention in rural and remote areas: an umbrella review	wrong publication type
100	McGrail, M. R.	2009	Geographical classifications to guide rural health policy in Australia	wrong publication type
101	McGrail, M. R.	2017	Index of access: a new innovative and dynamic tool for rural health service and workforce planning	wrong publication type
102	Mills, J.	2010	The status of rural nursing in Australia: 12 years on	wrong publication type
103	Millsteed, J.	2000	Issues affecting Australia's rural occupational therapy workforce	wrong publication type
104	Morelli, V.	2017	An Introduction to Primary Care in Underserved Populations: Definitions, Scope, and Challenges	wrong publication type
105	Morelli, V.	2019	Primary Care in Underserved Populations Definitions, Scope, Challenges and Future Considerations	wrong publication type
106	Moyo, S.	2018	Application of machine learning models in predicting length of stay among healthcare workers in underserved communities in South Africa	wrong population
107	Muhammad, K.	2014	Assessment of health workforce providing maternal neonatal child health services at primary health care level of Sanghar, Sindh	wrong population
108	Mulcahy, A. J.	2010	The impact of recent physiotherapy graduates in the workforce: a study of Curtin University entry-level physiotherapists 2000â€“2004	Not medical desert
109	Murphy, G.T.	2012	Research to action: an evaluation	Not medical desert
110	Nancarrow, S. A.	2005	Dynamic professional boundaries in the healthcare workforce	wrong publication type
111	Nelofer, A.	2013	Incentives scheme can be both beneficial & counterproductive: a systematic review of efficacy and values of health workers' incentives in health sector	wrong population
112	Nicholson, L. A.	2008	Rural mental health	wrong publication type
113	Nishiura, H.	2004	Health inequalities in Thailand: geographic distribution of medical supplies in the provinces	wrong population

Number	1st author	year	title	Reason for exclusion
114	Nomura, K.	2009	The shortage of pediatrician workforce in rural areas of Japan	wrong population
115	Nyamtema, A. S.	2011	Tanzanian lessons in using non-physician clinicians to scale up comprehensive emergency obstetric care in remote and rural areas	wrong population
116	Ohta, R.	2020	Rural homecare nurses challenges in providing seamless patient care in rural Japan	wrong population
117	Okoroafor, S. C.	2021	Estimating frontline health workforce for primary healthcare service delivery in Bauchi State, Nigeria	wrong population
118	Olsen, O. E.	2005	Human resources for emergency obstetric care in northern Tanzania: distribution of quantity or quality?	wrong population
119	Oosterbroek, T.A.	2017	Rural nursing preceptorship: an integrative review	wrong publication type
120	Palumbo, M.V.	2009	Retaining an aging nurse workforce: perceptions of human resource practice	Not medical desert
121	Pathman, D. E.	2005	Changes in age-adjusted mortality rates and disparities for rural physician shortage areas staffed by the National Health Service Corps: 1984-1998	not HWF
122	Petrovcic, R.	2016	Defining rural, remote and isolated practices: the example of Slovenia	wrong publication type
123	Phillips, R. L.	2007	The Canadian contribution to the US physician workforce	Not medical desert
124	Pierce, D.	2016	Mental health academics in rural and remote Australia	wrong publication type
125	Pitblado, R.	2012	Geographical distribution of rural health human resources	wrong publication type
126	Preston, B.	2009	The Australian nurse and midwifery workforce: issues, developments and the future	wrong publication type
127	Rafiq, A.	2005	Telemedicine for access to quality care on medical practice and continuing medical education in a global arena	wrong publication type
128	Rao, K. D.	2012	So many, yet few: Human resources for health in India	wrong population
129	Rao, K. D.	2016	Composition and distribution of the health workforce in India: estimates based on data from the National Sample Survey	wrong population
130	Rao, M.	2011	Human resources for health in India	wrong population
131	Roth, K.	2017	Legal structures of emergency medical services. The research project EMSiG "Preclinical Emergency Medical Services in Germany"	wrong publication type
132	Rubin, G.	1983	Primary health care workers: the rural health aide program in El Salvador	wrong population
133	Rutebemberwa, E.	2014	Financial interventions and movement restrictions for managing the movement of health workers between public and private organizations in low- and middle-income countries	wrong population
134	Salgado-de Snyder, N.	2003	A model for integrating mental healthcare resources in the rural population of Mexico	wrong population
135	Sato, M.	2017	Measuring three aspects of motivation among health workers at primary level health facilities in rural Tanzania	wrong population
136	Sawaengdee, K.	2017	Factors associated with the choice of public health service among nursing students in Thailand	wrong population

Number	1st author	year	title	Reason for exclusion
137	Scheil-Adlung, X.	2015	Health sector employment: a tracer indicator for universal health coverage in national social protection floors	wrong population
138	Schubert, N.	2018	International approaches to rural generalist medicine: a scoping review	wrong publication type
139	Shah, S. M.	2016	Motivation and Retention of Physicians in Primary Healthcare Facilities: A Qualitative Study From Abbottabad, Pakistan	wrong population
140	Shaikh, B. T.	2013	Political and economic unfairness in health system of Pakistan: a hope with the recent reforms	wrong population
141	Shen, C.	2020	Measuring spatial accessibility and within-province disparities in accessibility to county hospitals in Shaanxi Province of Western China based on web mapping navigation data	wrong population
142	Shivam, S.	2014	Nursing personnel planning for rural hospitals in Burdwan District, West Bengal, India, using workload indicators of staffing needs	wrong population
143	Singh, A.	2019	Shortage and inequalities in the distribution of specialists across community health centres in Uttar Pradesh, 2002-2012	wrong population
144	Sirili, N. K.	2019	"Doctors ready to be posted are jobless on the street" the deployment process and shortage of doctors in Tanzania	wrong population
145	Skapetis, T.	2018	Patient satisfaction and an international graduate workforce programme	Not medical desert
146	Skillman, S.M.	2006	Characteristics of registered nurses in rural versus urban areas: implications for strategies to alleviate nursing shortages in the United States	wrong publication type
147	Smith, J.D.	2008	Defining remote medical practice	wrong publication type
148	Smith, M. W.	1978	The distribution of medical care in central California: A social and economic analysis	not HWF
149	Smith, R.	2014	Will There Be a Good General Surgeon When You Need One? (Part II) Solutions and Taking Back General Surgery	Not medical desert
150	Snyder, J. E.	2017	Defining Rurality in Medicare Administrative Data	wrong publication type
151	Song, P.	2016	Inequality of Paediatric Workforce Distribution in China	wrong population
152	Sousa, A.	2012	Monitoring inequalities in the health workforce: The case study of brazil 1991-2005	wrong population
153	Strasser, R.	2016	Rural health care access and policy in developing countries	wrong population
154	Tabari-Khomeiran, R.	2019	Inequity in the distribution of rural family physicians in Iran: a cross sectional study	wrong population
155	Tangcharoensathien, V.	2013	Health workforce contributions to health system development: A platform for universal health coverage	wrong population
156	Tanihara, S.	2011	Urbanization and physician maldistribution: a longitudinal study in Japan	wrong population
157	Tao, Y.	2014	Methods for measuring horizontal equity in health resource allocation: a comparative study	wrong population
158	Taylor, B.	2018	Who are community health workers and what do they do? Development of an empirically derived reporting taxonomy	wrong population

Number	1st author	year	title	Reason for exclusion
159	Taylor, C. E.	1992	Surveillance for equity in primary health care: policy implications from international experience	wrong population
160	Taylor, J. M.	1978	Keeping up with...planning rural health care: some examples from Maine	wrong publication type
161	Tseng, M. H.	2021	Accessibility assessment of community care resources using maximum-equity optimization of supply capacity allocation	wrong population
162	Vasan, A.	2017	Support and performance improvement for primary health care workers in low- and middle-income countries: a scoping review of intervention design and methods	not HWF
163	Vindigni, S. M. N.	2014	Kenya's emergency-hire nursing programme: a pilot evaluation of health service delivery in two districts	wrong population
164	Vujicic, M.	2012	An analysis of GAVI, the Global Fund and World Bank support for human resources for health in developing countries	wrong population
165	Wang, S.	2018	Trends in health resource disparities in primary health care institutions in Liaoning Province in Northeast China	wrong population
166	Wang, X.	2018	Spatial accessibility of primary health care in China: A case study in Sichuan Province	wrong population
167	Wang, Y.	2020	The disequilibrium in the distribution of the primary health workforce among eight economic regions and between rural and urban areas in China	wrong population
168	Wei, L.	2017	Tracking rural health service quality and evaluation mechanism: a bibliometric analysis	foreign language
169	Williams, E. N.	2014	Building capacity in the rural physiotherapy workforce: a paediatric training partnership	wrong publication type
170	Wiseman, V.	2017	Measuring inequalities in the distribution of the Fiji Health Workforce	wrong population
171	Wong, H. T.	2016	Spatial illustration of health-care workforce accessibility index in China: How far has our 2009 health-care reform brought us?	wrong population
172	Wu, H. C.	2018	Evaluating disparities in elderly community care resources: Using a geographic accessibility and inequality index	wrong population
173	Xu, K.	2014	Trend in distribution of primary health care professionals in Jiangsu province of eastern China	wrong population
174	Yang, L.	2019	What about the health workforce distribution in rural China? An assessment based on eight-year data	wrong population
175	Yang, L.	2019	Is the health workforce distribution in Beijing, China perfectly equitable?	wrong population
176	Zeng, W.	2018	The health resource allocation and equity of pediatric care in Sichuan province: a cross-sectional study	wrong population
177	Zhang, D.	2020	Assessment of changes in rural and urban primary care workforce in the united states from 2009 to 2017	wrong publication type
178	Zhou, K.	2015	Inequality trends of health workforce in different stages of medical system reform (1985-2011) in China	wrong population

Number	1st author	year	title	Reason for exclusion
179	Zhu, A.	2019	Analysis of strategies to attract and retain rural health workers in Cambodia, China, and Vietnam and context influencing their outcomes	wrong population
180	Zhu, B.	2018	Detecting the priority areas for health workforce allocation with LISA functions: an empirical analysis for China	wrong population
181	Zubieta, L.	2010	Factors of primary care demand: a case study	wrong publication type

1 Al Saffer Q, Al-Ghaith T, Alshehri A, Al-Mohammed R, Al Homidi S, Hamze MM, Herbst CH, Alazemi N. The capacity of primary health care facilities in Saudi Arabia: infrastructure, services, drug availability, and human resources. BMC Health Serv Res. 2021;21(1):365.

2 Alameddine M, Khodr H, Mourad Y, Yassoub R, Ramia JA. Upscaling the recruitment and retention of human resources for health at primary healthcare centres in Lebanon: a qualitative study. Health Soc Care Community. 2016;24(3):353-362.

3 Alvi R. Experience of developing rural surgical care in a remote mountainous region of Pakistan: Challenges and opportunities. Annals of Tropical Medicine and public health. 2011;4(1):57.

4 Ament LA. The integration of a QA/RM program in a rural healthcare obstetrical service. J Qual Assur. 1991;16(6):20-22.

5 Averill JB. Getting started: initiating critical ethnography and community-based action research in a program of rural health studies. Int J Qual Methods. 2006;5(2):17-27.

6 Ayaz A, Bahadur S. Brain drain human resources and human capital growth in Pakistan: a political and economical perspective. Pak J Public Health. 2014;4(1)

7 Badanta-Romero B, Moreno-Moreno B, Soto-Diaz V, Barrientos-Trigo S. Cuidados enfermeros para el abordaje de la salud comunitaria en población indígena de la Amazonia peruana. Enfermería Clínica 2021;31(1):57-63.

8 Bahadori M. Shams L, Sadeghifar J, Hamouzadeh P, Nejati M. Classification of health structural indicators using scalogram model in Golestan province, northern Iran. Iran J Public Health. 2012;41(5):58-65.

9 Bamberg R, Malvey D, Wainright C, Fottler MD, Joiner CL. The development of a state-level health manpower database using an employer-based survey: A pilot project. J Rural Health. 1994;10(3):199-207.

10 Bärnighausen T, Bloom DE. Financial incentives for return of service in underserved areas: A systematic review. BMC Health Serv Res. 2009;9:86.

11 Barzola-Cordero V, Llamosas E, Echegaray C, Cuzco M, Peralta F. Implementation of medical specialists' brigades in the areas of Universal Health coverage: The Peruvian Ministry of Health experience, 2009-2010. Rev Peru Med Exp Salud Publica. 2011;28(2):352-361.

12 Batura N, Skordis-Worrall J, Thapa R, Basnyat R, Morrison J. Is the Job Satisfaction Survey a good tool to measure job satisfaction amongst health workers in Nepal? Results of a validation analysis. BMC Health Serv Res. 2016;16:308.

13 Benskin, LLL. A Concept Development of the Village Health Worker. Nurs Forum. 2012;47(3):173-182.

14 Berke EM, West AN, Wallace AE, Weeks WB. Practical and policy implications of using different rural-urban classification systems: A case study of inpatient service utilization among veteran administration users. J Rural Health. 2009;25(3):259-266.

15 Bertrand WE, Micklin M. Attitudinal classification of health manpower in Colombia: A research note on urban/rural differences in occupational prestige. Soc Sci Med. 1979;13A(2):235-240.

16 Bhan, N, McDougal L, Singh A, Atmavilas Y, Raj A. Access to women physicians and uptake of reproductive, maternal and child health services in India. EClinicalMedicine. 2020;5:20:100309.

17 Brahmapurkar KP, Zodpey SP, Sabde YD, Brahmapurkar VK. The need to focus on medical education in rural districts of India. Natl Med J India. 2018;31(3):164-168.

18 Breindel CL, Goldberg DM. Marketing strategies in rural areas. J Ambul Care Mange. 1981;4(4):15-29.

19 Brown MC. Using Gini-style indices to evaluate the spatial patterns of health practitioners: Theoretical considerations and an application based on Alberta data. Soc Sci Med. 1994;38(9):1243-1256.

20 Brown P. Place matters. Aust J Rural Healht. 2016;24(1):67.

21 Bryant M, Essomba RO. Measuring time utilization in rural health centres. Health Policy Plan 1995;10(4):415-422.

22 Burrows G, Calleja P, Cooke M. What are the support needs of nurses providing emergency care in rural settings as reported in the literature? A scoping review. Rural Remote Health. 2019;19(2):4805.

23 Cancedda C, Farmer PE, Kyamanywa P, Riviello R, Rhatigan J, Wagner CM, Ngabo F, Anatole M, Drobac PC< Mpunga T, Nutt CT, Baptiste Kakoma J, Mukherjee J, Cortas C, Condo J, Ntaganda F, Bukhman G, Binagwaho A. Enhancing formal educational and in-service training programs in rural Rwanda: A partnership among the public sector, a nongovernmental organization, and Academia. Acad Med. 2014;89(9):1117-1124.

24 Carson DB, Wenghover E, Timony PE, School A, Charters B, White D, Vuin A, Garrett J. Recruitment and retention of professional labour: thehealth workforce at settlement level. In book: Settlements at the Edge: remote human settlements in developed nations Publisher: Edward Elgar Publishing Editors: A Taylor, Dean Carson, P Ensign, R Rasmussen, T Huskey, G Eilmsteiner-Saxinger

25 Casey MM, Wellever A, Moscovice I. Rural health network development: Public policy issues and state initiatives. J Health Polit Policy Law. 1997;22(1):23-47.

26 Celletti F, Wright A, Palen J, Frehywot S, Markus A, Greenberg A, Teixeira de Aguiar RA, Campos F, Buch E, Samb B. Can the deployment of community health workers for the delivery of HIV services represent an effective and sustainable response to health workforce shortages? Results of a multicountry study. AIDS 2010;24(1):S45-57.

27 Cheek C, Hays R, Allen P, Walker G, Shires L. Building a local medical workforce in Tasmania: where are international fee-paying medical graduates likely to work? Rural Remote Health 2017;17(3):4292.

28 Cheek C, Hays R, AllenP, Walker G, Shires L. Building a medical workforce in Tasmania: A profile of medical student intake. Aust J Rural Health. 2019;27(1):28-33.

29 Chen F, Zhang PL, Zheng WH, Zhong XN, Xu X, Tan HW. Analysis of the equity of public health resources allocation in Chongqing from the perspective of migration. Journal of Shanghai Jiaotong University. 2015;35(8):1207-1212.

30 Chowdhury M. The role of traditional birth attendants in a safe delivery programme in Bangladesh. Trop Doct. 1998;28(2):104-106.

31 Colagiuri R. Building capacity to reduce diabetes complications in the Pacific: The Vanuatu experience so far. Practical Diabetes 2006 https://doi.org/10.1002/pdi.1006

32 Collins C. Challenges of Recruitment and Retention in Rural Areas. N C Med J. 2016;77(2):99-101.

33 Cossman J, James W, Wolf JK. The differential effects of rural health care access on race-specific mortality. SSM Popul Health 2017;3:618-623.

34 Curry LA, Alpern R, Webster TR, Byam P, Zerihun A, Tarekeshwar N, Cherlin EJ, Bradley EH. Community perspectives on roles and responsibilities for strengthening primary health care in rural Ethiopia. Glob Public Health 2012;7(9):961-973.

35 Dayrit MM, Dolea C, Dreesch N. Addressing the Human Resources for Health crisis in countries: How far have we gone? What can we expect to achieve by 2015? Rev Peru Med Exp Salud Publica. 2011;28(2):327-336.

36 DeFriese GH. An agenda for health services research in primary care. Health Care Manage Rev. 1981;6(3):49-54.

37 Deng F, Lv JH, Wang HL, Gao JM, Zhou ZL. Expanding public health in China: an empirical analysis of healthcare inputs and outputs. Public Health. 2017;142:73-84.

38 Dhingra B, Dutta AK. National rural health mission. Indian J Pediatr. 2011;78(12):1520-1526.

39 Dhungel, B. Planning for rural health services in Nepal. 1988

40 Dodson ZM, Agadjanian V, Driessen J. How to allocate limited healthcare resources: Lessons from the introduction of antiretroviral therapy in rural Mozambique. Appl Geogr. 2017;78:45-54.

41 Dolea C, Stormont L, Braichet JM. Evaluated strategies to increase attraction and retention of health workers in remote and rural areas. Bull World Health Organ. 2010;88(5):379-385.

42 Dong E, Liu S, Chen M, Wang H, Chen L, Xu T, Wang T, Zhang L. Differences in regional distribution and inequality in health-resource allocation at hospital and primary health centre levels: A longitudinal study in Shanghai, China. BMJ Open 2020;10(17):e035635.

43 Drennan VM, Ross, F. Global nurse shortages - The facts, the impact and action for change. Br Med Bull. 2019;130(1):25-37.

44 Duarte-Gómez MB, Nunez-Urquiza RM, Restrepo-Restrepo JA, Richardson-Lopez-Collada. VL. Social determinants of infant mortality in socioeconomic deprived rural areas in Mexico. Bol Med Hosp Infant Mex. 2015;72(3):181-189.

45 Eley DS, Baker PG. Will Australian rural clinical schools be an effective workforce strategy? Early indications of their positive effect on intern choice and rural career interest. Med J Aust. 2007;187(3):166-167.

46 El-Jardali F, Alameddine M, Jamal D, Dimassi D, Dumit NY, McEwen MK, Jaafar M, Murray SF. A national study on nurses' retention in healthcare facilities in underserved areas in Lebanon. Hum Resour Health. 2013;11:49.

47 Erchick DJ, Agrawal NK, Khatry SK, Katz J, LeClerq SC, Rai B, Reynolds MA, Mullany LC. Feasibility of training community health workers to conduct periodontal examinations: a validation study in rural Nepal. BMC Health Serv Res. 2020;20(1):412.

48 Erdenee O, Paramita SA, Yamazaki C, Koyama H. Distribution of health care resources in Mongolia using the Gini coefficient. Hum Resour Health. 2017:15(1):56.

49 Erickson JS. Rural medical education in the WWAMI region: instituting a rural longitudinal medical school curriculum in association with a rural longitudinal integrated community clerkship experience. Int j Child Health Hum Dev. 2011;4(1):91-100.

50 Fang P, Dong S, Xiao J, Liu C, Feng X, Wang Y. Regional inequality in health and its determinants: Evidence from China. Health Policy. 2010;94(1):14-25.

51 Fang P, Liu X, Huang L, Zhang X, Fang Z. Factors that influence the turnover intention of Chinese village doctors based on the investigation results of Xiangyang City in Hubei Province. Int J Equity Health. 2014;13:84.

52 Farmer J, Kenny A, McKinstry C. Huysmans RD. A scoping review of the association between rural medical education and rural practice location. Hum Resour Health. 2015;13:27.

53 Farmer JC, Baird A, Iversen L. Rural deprivation: Reflecting reality. Br J Gen Pract. 2001;51(467):486-491.

54 Federal register 2002 Medicare program; Announcemetn of the Calendar Year 2002 Conversion Factor for the hospital outpatient prospective payment system and a pro rata reduction on transitional pass-through payments. 2002 Centers for medicare & Medicaid services. Federal Register :: Medicare Program; Announcement of the Calendar Year 2002 Conversion Factor for the Hospital Outpatient Prospective Payment System and a Pro Rata Reduction on Transitional Pass-Through Payments

55 Francis JR, Verma S, Bonney D. Disparity in distribution of inpatient hospital services in Australia. Aust N Z J Public Health. 2020;44(4):326.

56 Freeman R, Lush C, MacGillveray S, Themessl-Huber M, Richards D. Dental therapists/hygienists working in remote-rural primary care: A structured review of effectiveness, efficiency, sustainability, acceptability and affordability. Int Dent J. 2013;63(2):103-112.

57 Fujiwara K, Yagahara A, Tanikawa T, Tani Y, Ohba Hiateru, Ogasawara K. Trends for the Geographic Distribution of Radiological Resources in Hokkaido, Japan: Data Analysis Using Gini Coefficient and Herfindahl-Hirschman Index. Nihon Hoshasen Gijutsu Gakkai Zasshi. 2016;72(10):970-977

58 Gajewski J, Cheelo M, Bijlmakers L, Kachimba J, Pittalis C, Brugha R. The contribution of non-physician clinicians to the provision of surgery in rural Zambia-a randomised controlled trial. Hum Resour Health. 2019;17(1):60.

59 Ghosh S. Primary health care for developing countries. 1979

60 Gibson OR, Segal L, McDermott RA. A systematic review of evidence on the association between hospitalisation for chronic disease related ambulatory care sensitive conditions and primary health care resourcing. BMC Health Serv Res. 2013;13:336.

61 Giovanella L, Fidelis de Almeida P. Comprehensive primary care and segmented health systems in South America. Cad Saude Publica. 2017;33(2):e00118816.

62 Giraldo Molina CI. Nursing care needs: criteria to define nursing personnel requirements. Invest Educ Enferrm. 2000;18(1):49-68.

63 Godwin D, Hoang H, Crocombe LA, Bell E. Dental practitioner rural work movements: a systematic review. Rural Remote Health. 2014;14(3):2825.

64 Greer TM, Talley RC. Addressing Disparities in Rural Health. 2011. 10.1007/978-1-4614-0302-9_5

65 Greiner DS, Glick DF, Kulbok PA, McKim Mitchel E. Rural health nursing research review: global perspectives. Annu Rev Nurs Res. 2008;26:261-294.

66 Grobler L, Marais BJ, Mabunda S. Interventions for increasing the proportion of health professionals practising in rural and other underserved areas. Cochrane Database Syst Rev. 2015(6):CD005314.

67 Gross JM, Riley PL, Kiriinya R. Rakuom C, Willy R, Kamenju A, Oywer E, Wambua D, Waudo A, Rogers MF. The impact of an emergency hiring plan on the shortage and distribution of nurses in Kenya: The importance of information systems. Bull World Health Organ. 2010;88(11):824-830.

68 Hagopian A, Mohanty MK, Das A, House PJ. Applying WHO's 'workforce indicators of staffing need' (WISN) method to calculate the health worker requirements for India's maternal and child health service guarantees in Orissa State. Health Policy Plan. 2012;27(1):11-18.

69 Han Y, Wei J, Song X, Sarah BJ, Wen C, Zheng X. Accessibility of primary health care workforce in rural China. Asia Pac J Public Health. 2012;24(5):833-847.

70 Hatam N, Kafashi S, Kavosi Z. Distribution of health resource allocation in the Fars province using the scalogram analysis technique in 2011. Iran J Med Sci. 2015;40(4):356-361.

71 Hays R. Interpreting rural career intention in medical workforce research. Educ Prim Care. 2017;28(1):7-9.

72 Hays RB. Rural medical education in Europe: the relevance of the Australian experience. Rural Remote Health. 2007;7(1):683.

73 Hays RB, Veitch PC, Franklin L. Crossland L. Methodological issues in medical workforce analysis: implications for regional Australia. 1998;6(1):32-35.

74 Hermawan A. Health Workforce Distribution (Physicians, Nurses Midwives) Analysis in Indonesia 2013 by Gini Index. Buletin Penelitian Sistem Kesehatam. 2019;22.

75 Higgins-Steele A, Waller K, Fotso JC, Vesel L. Peer-driven quality improvement among health workers and traditional birth attendants in Sierra Leone: linkages between providers' organizational skills and relationships. BMC Health Serv Res. 2015;15:S4.

76 Hsu YE, Lin W, Tien JJ, Tzeng LY. Measuring inequality in physician distributions using spatially adjusted Gini coefficients. Int J Qual Health Care. 2016;28(6):657-664.

77 Humphreys J. Rural health status: what do statistics show that we don't already know? Aust J Rural Health. 1999;7(1):60-63.

78 Hunter D, Kunjumen T, Gupta N, Quain E, Soucat A. Boundaries of the health workforce: definition and classification of health workers. Medicine 2009.

79 Ireland M, Cavalini L, Girardi S, Araujo EC, Lindelow M. Expanding the primary health care workforce through contracting with nongovernmental entities: the cases of Bahia and Rio de Janeiro. Hum Resour Health. 2016;14:6.

80 Ishikawa T, Fujiwara K, Ohba H, Suzuki T, Ogasawara K. Forecasting the regional distribution and sufficiency of physicians in Japan with a coupled system dynamics-geographic information system model. Hum Resour Health. 2017;15(1):64.

81 Judd FK, Jackson H, Davis J, Cockram A, Komiti A, Allen N, Murray G, Kyrios M, Hodgins G. Improving access for rural Australians to treatment for anxiety and depression: the University of Melbourne Depression and Anxiety Research and Treatment Group initiative. Aust J Rural Health 2001;9(2):91-96.

82 Kadam S., Nallala S, Zodpqy S, Pati S, Akthar Hussain M, Singh Chauhan A, Das S, Martineau T. A study of organizational versus individual needs related to recruitment, deployment and promotion of doctors working in the government health system in Odisha state, India. Hum Resour Health 2016;14:7.

83 Kashima S, Inoue K, Matsumoto M, Takeuchi K. Non-physician communities in Japan: are they still disadvantaged? Rural Remote Health. 2014;14(3):2907.

84 Kebriaei A, Moteghedi MS. Job satisfaction among community health workers in Zahedan District, Islamic Republic of Iran. East Mediterr Health J. 2009;15(5):1156-1163.

85 Kelman I, Harris M. Linking Disaster Risk Reduction and Healthcare in Locations with Limited Accessibility: Challenges and Opportunities of Participatory Research. Int J Environ Res Public Health. 2020;18(1):248.

86 Kerr L, Kealy B, Lim D, Walters L. Rural emergency departments: a systematic review to develop a resource typology relevant to developed countries. Aust J Rural Health. 2021;29(1):7-20.

87 Knippenberg R, Alihonou E, Soucat A, Oyegbite K, Calivis M, Hopwood I, Niimi R, Diallo MP, Conde M, Ofosu-Amaah S. Implementation of the Bamako initiative: Strategies in Benin and Guinea. Int J Healht Plann Manage. 1997;12:S29-47.

88 Kolehmainen-Aitken RL, Shipp P. 'Indicators of staffing need': Assessing health staffing and equity in Papua New Guinea. Health Policy and Planning 1990;5(2):167-176.

89 Leight SB.To describe the application of the vulnerable populations conceptual model to rural health. Public Health Nurs. 2003;20(6):440-448.

90 Leonfanti FL, Chiesa ME. euquen, Argentina: Provincial health policies and their results. The Journal of rural health (USA). 1990;4(1):59-69.

91 Lin SW, Yen C, Chiu T, Chi W, Liou T. New indices for home nursing care resource disparities in rural and urban areas, based on geocoding and geographic distance barriers: a cross-sectional study. Int J Health Georg. 2015;14:28.

92 Liu T, Li J, Chen J, Yang S. Regional Differences and Influencing Factors of Allocation Efficiency of Rural Public Health Resources in China. Healthcare (Basel). 2020;8(3):270.

93 Liu W, Xia Y, Hou J. Health expenditure efficiency in rural China using the super-SBM model and the Malmquist productivity index. Int J Equity Health. 2019;18(1):111.

94 Lohmann J, Shulenbayev O, Wilhelm D, Muula AS, De Allegri M. Psychological wellbeing in a resource-limited work environment: examining levels and determinants among health workers in rural Malawi. Hum Resour Health. 2019;17(1):85.

95 Lourenco AEP. The meaning of 'rural' in rural health: A review and case study from Brazil. Glob Public Health. 2012;7(1):1-13.

96 Lysdahl KB, Borretzen I. Geographical variation in radiological services: a nationwide survey. BMC Health Serv Res. 2007;7:21.

97 Martiniuk AL, Colbran R, Ramsden R, Edwards M, Barrett E, O'Callaghan E, Bullock R, Lowe EFL, Karlson D, Curnow J, Gotch B, Kramer J, Bagnulo S, Rothnie IP, Hardaker L, Turner N, Wotherspoon A, Russell C. Capability ... what's in a word? Rural Doctors Network of New South Wales Australia is shifting to focus on the capability of rural health professionals. Rural Remote Health. 2020;20(3):5633.

98 Matsumoto M, Kashima S, Ogawa T, Takeuchi K. Do rural and remote areas really have limited accessibility to health care? Geographic analysis of dialysis patients in Hiroshima, Japan. Rural Remote Health. 2013;13(3):2507.

99 Mbemba G, Gagnon M, Pare G, Cote J. Interventions for supporting nurse retention in rural and remote areas: an umbrella review. Hum Resour Health. 2013;11:44.

100 McGrail MR, Hunphreys JS. Geographical classifications to guide rural health policy in Australia. Aust New Zealand Health Policy. 2009;6:28.

101 McGrail MR, Russell DJ, Humphreys JS. Index of access: a new innovative and dynamic tool for rural health service and workforce planning. Aust Health Rev. 2017;41(5):492-498.

102 Mills J, Birks M, Hegney D. The status of rural nursing in Australia: 12 years on. Collegian. 2010;17(1):30-37.

103 Millsteed J. Issues affecting Australia's rural occupational therapy workforce. Aust J Rural Health. 2000;8(2):73-76.

104 Morelli V. An Introduction to Primary Care in Underserved Populations: Definitions, Scope, and Challenges. Prim Care 2017;44(1):1-9.

105 Morelli V. Primary Care in Underserved Populations Definitions, Scope, Challenges and Future Considerations. Physician Assistant Clinics. 2019;4(1):1-9.

106 Moyo S, Doan TN, Yn JA, Tshuma N. Application of machine learning models in predicting length of stay among healthcare workers in underserved communities in South Africa. Hum Resour Health. 2018;16(1):68.

107 Kashif M, Kumar R, Sikander S, Sultan J, Ghaffar N. Assessment of health workforce providing maternal neonatal child health services at primary health care level of Sanghar, Sindh. Pak J Public Health. 2014;4(1):20-23.

108 Mulcahy AJ, Jones S, Strauss G, Cooper I. The impact of recent physiotherapy graduates in the workforce: a study of Curtin University entry-level physiotherapists 2000-2004. Aust Health Rev. 2010;34(2):252-259.

109 Murphy GT, Alder R, MacKenzie A, Cook A, Maddalena V. Research to action: an evaluation. Nurs Leadersh (Tor Ont). 2012:21-32.

110 Nancarrow SA, Borthwick AM. Dynamic professional boundaries in the healthcare workforce. Sociol Health Illn. 2005;27(7):897-919.

111 Nelofer A, Kumar R. Incentives scheme can be both beneficial & counterproductive: a systematic review of efficacy and values of health workers' incentives in health sector. Pak J Public Health. 2013;3(3):35-40.

112 Nicholson LA. Rural mental health. Advances in Psychiatric Treatment. 2008;14(4):302-311.

113 Nishiura H, Barua S, Lawpoolsri S, Kittitrakul C, Leman MM, Maha MS, Muangnoicharoen S. Health inequalities in Thailand: geographic distribution of medical supplies in the provinces. Southeast Asian J Trop Med Public Health. 2004;35(3):735-740.

114 Nomura K, Inoue S, Yano E. The shortage of pediatrician workforce in rural areas of Japan. Tohoku J Exp Med. 2009;217(4):299-305.

115 Nyamtema, AS, Pemba SK, Mbaruku G, Rutasha FD, Roosmalen J van. Tanzanian lessons in using non-physician clinicians to scale up comprehensive emergency obstetric care in remote and rural areas. Hum Resour Health. 2011:9;28.

116 Ohta, R, Ryu Y, Katsube T, Sano C. Rural homecare nurses challenges in providing seamless patient care in rural Japan. Int J Environ Res Public Health. 2020;17(24):9330.

117 Okoroafor SC, Ingom M, Mohammed B, Salihu D, Ahmat A, Osubor M, Nyoni J, Dayyabu H, Alemu W. Estimating frontline health workforce for primary healthcare service delivery in Bauchi State, Nigeria. J Public Health (Oxf) 2021;43(1):i4-i11.

118 Olsen OE, Ndeki S, Norheim OF. Human resources for emergency obstetric care in northern Tanzania: distribution of quantity or quality? Hum Resour Health. 2005;3:5.

119 Oosterbroek TA, Yonge O, Myrick F. Rural nursing preceptorship: an integrative review. Journal of Rural Nursing and Health Care 2017:17(1).

120 Palumbo MV, McIntosh B, Rambur B, Naud S. Retaining an aging nurse workforce: perceptions of human resource practice. Nurs Econ. 2009;27(4):221-227.

121 Pathman DE, Fryer GE, Green LA, Philips RL. Changes in age-adjusted mortality rates and disparities for rural physician shortage areas staffed by the National Health Service Corps: 1984-1998. J Rural Health 2005;21(3):214-220.

122 Petrovcic R. Defining rural, remote and isolated practices: the example of Slovenia. Family Medicine and Primary Care Review. 2016;18(3):391-393.

123 Phillips RL, Petterson S, Fryer Jr GE, Rosser W. The Canadian contribution to the US physician workforce. CMAJ 2007;176(8):1083-1087.

124 Pierce D, Little F, Bennett-Levy J, Isaacs AN, Bridgman H, Lutkin SJ, Carey TA, Schlicht KG, McCabe-Gusta ZP, Martin E, Martinez LA. Mental health academics in rural and remote Australia. Rural Remote Health 2016;16(3):3793.

125 Pitblado, J.R. (2012). Geographical distribution of rural health human resources. Chapter 5, pp 83-100, in J.C. Kulig and A.M. Williams, eds. Health in Rural Canada. Vancouver, British Columbia: UBC Press.

126 Preston B. The Australian nurse and midwifery workforce: issues, developments and the future. Collegian 2009;16(1):25-34.

127 Rafiq A, Merrell RC. Telemedicine for access to quality care on medical practice and continuing medical education in a global arena. J Contin Educ Health Prof. 2005;25(1):34-42.

128 Rao KD, Bhatnagar A, Berman P. So many, yet few: Human resources for health in India. Hum Resour Health 2012;10:19.

129 Rao KD, Shahrawat R, Bhatnagar A. Composition and distribution of the health workforce in India: estimates based on data from the National Sample Survey. WHO South East Asia J Public Health. 2016;5(2):133-140.

130 Rao M, Rao KD, Kumar AKS, Chatterjee M, Sandararaman T. Human resources for health in India. Lancet 2011;377(9765):587-598.

131 Roth K, Baier N, Henschke C, Felgner S. Legal structures of emergency medical services. The research project EMSiG "Preclinical Emergency Medical Services in Germany". Notfall & Rettungsmedizin 2016;20(3).

132 Rubin G, Chen C, Herrera Y de, Aparicio V de, Massey J, Morris L. Primary health care workers: the rural health aide program in El Salvador. Bull Pan Am Health Organ. 1983;17(1):42-50.

133 Rutebemberwa E, Kinengyere AA, Ssengooba F, Pariyo GW, Kiwanuka SN. Financial interventions and movement restrictions for managing the movement of health workers between public and private organizations in low- and middle-income countries. Cochrane Database Syst Rev. 2014(2):CD009845.

134 Salgado-de Snyder N, Jesus Diaz-Perez m de, Gonzalez-Vazquez T. A model for integrating mental healthcare resources in the rural population of Mexico. Salud Publica Mex. 2003;45(1):19-26.

135 Sato M, Maufi D, Mwingira UJ, Leshabari MT, Ohnisi M, Honda S. Measuring three aspects of motivation among health workers at primary level health facilities in rural Tanzania. Plos One 2017;12(5):e0176973.

136 Sawaengdee K, Pudpong N, Wisaijohn T, Suphanchaimat R, Putthasri W, Lagarde M, Blaauw D. Factors associated with the choice of public health service among nursing students in Thailand. BMC Nurs 2017;16:8.

137 Scheil-Adlung X, Behrendt T, Wong L. Health sector employment: a tracer indicator for universal health coverage in national social protection floors. Hum Resour Health 2015;13:66.

138 Schubert N, Evans R, Battye K, Sen Gupta T, Larkins S, McIver L. International approaches to rural generalist medicine: a scoping review. Hum Resour Health 2018;16(1):62.

139 Shah SM, Zaidi S, Ahmed J, Rehman SU. Motivation and Retention of Physicians in Primary Healthcare Facilities: A Qualitative Study From Abbottabad, Pakistan. Int J Health Policy Manag. 2016;5(8):467-475.

140 Shaikh BT, Ejaz I, Achakzai DK, Shafiq Y. Political and economic unfairness in health system of Pakistan: a hope with the recent reforms. J Ayub Med Coll Abbottabad. 2013;25(1-2):198-203.

141 Shen C, Zhou Z, Lai S, Lu L, Dong W, Su M, Zhang J, Wang X, Deng Q, Chen Y, Chen X. Measuring spatial accessibility and within-province disparities in accessibility to county hospitals in Shaanxi Province of Western China based on web mapping navigation data. Int J Equity Health. 2020;19(1):99.

142 Shivam S, Nath Roy R, Dasgupta S, Das Bhattacharyya K, Nath Misra R, Roy S, Indranil S. Nursing personnel planning for rural hospitals in Burdwan District, West Bengal, India, using workload indicators of staffing needs. J Health Popul Nutr. 2014;32(4):658-664.

143 Singh A. Shortage and inequalities in the distribution of specialists across community health centres in Uttar Pradesh, 2002-2012. BMC Health Serv Res. 2019;19(1):331.

144 Sirili NK, Frumence G, Kiwara A, Mwanga M, Goicolea I, Hurtig A. "Doctors ready to be posted are jobless on the street" the deployment process and shortage of doctors in Tanzania. Hum Resour Health. 2019;17(1):11.

145 Skapetis T, Ajwani S, Bhole S. Patient satisfaction and an international graduate workforce programme. Int J Health Gov. 2013;23(3):243-251.

146 Skillman SM, Palazzo L, Keepnews D, Hart LG. Characteristics of registered nurses in rural versus urban areas: implications for strategies to alleviate nursing shortages in the United States. J Rural Health 2006;22(2):151-157.

147 Smith JD, Margolis SA, Ayton J, Ross V, Chalmers E, Giddings P, Baker L, Kelly M, Love C. Defining remote medical practice. Med J Aust 2008;188(3):159-161.

148 Smith MW. The distribution of medical care in central California: A social and economic analysis. Volume 1. University of California Berkely 1977.

149 Smith R, Stain SC, McFadden DW, Finlayson SRG, Jones DB, Public Policy & Advocacy Committee of the SSAT; Reid-Lombardo KM. Will There Be a Good General Surgeon When You Need One? (Part II) Solutions and Taking Back General Surgery. J Gastrointest Surg 2014;18(7):1334-1342.

150 Snyder JE, Jensen M, Nguyen NX, Filice CE, Joynt KE. Defining Rurality in Medicare Administrative Data. Med Care 2017;55(12):e164-e169.

151 Song P, Ren Z, Chang X, Liu X, An L. Inequality of Paediatric Workforce Distribution in China. Int J Environ Res Public Health. 2016;13(7):703.

152 Sousa A, Dal Poz MR, Leite Carvalho C. Monitoring inequalities in the health workforce: The case study of Brazil 1991-2005. Plos One 2012;7(3):e33399.

153 Strasser R, Kam SM, Regalado SM. Rural health care access and policy in developing countries. Annu Rev Public Health. 2016;37:395-412.

154 Tabari-Khomeiran R, Kiger A, Parsa-Yekta Z, Ahmadi F. Inequity in the distribution of rural family physicians in Iran: a cross sectional study. 2019;38(5):211-218.

155 Tangcharoensathien V, Limwattananon S, Suphanchaimat R, Patcharanarumol W, Sawaengdee K, Putthasri W. Health workforce contributions to health system development: A platform for universal health coverage. Bull World Health Organ 2013;91(11):874-880.

156 Tanihara S. Kobayashi Y, Une H, Kawachi I. Urbanization and physician maldistribution: a longitudinal study in Japan. BMC Health Serv Res. 2011;11:260.

157 Tao Y, Henry K, Zou Q, Zhong X. Methods for measuring horizontal equity in health resource allocation: a comparative study. Health Econ Rev. 2014;4(1):10.

158 Taylor B, Mathers J, Parry J. Who are community health workers and what do they do? Development of an empirically derived reporting taxonomy. J Public Health (Oxf) 2018;40(1):199-209.

159 Taylor CE. Surveillance for equity in primary health care: policy implications from international experience. Int J Epidemiol. 1992;21(6):1043-1049.

160 Taylor JM. Keeping up with...planning rural health care: some examples from Maine. J Ambul Care Manage 1978;1(1):89-97.

161 Tseng MH, Wu HC. Accessibility assessment of community care resources using maximum-equity optimization of supply capacity allocation. Int J Environ Res Public Health. 2021 18(3):1153.

162 Vasan A, Mabey DC, Chaudri S, Brown Epstein H, Lawn SD. Support and performance improvement for primary health care workers in low- and middle-income countries: a scoping review of intervention design and methods. Health Policy Plan 2017;32(3):437-452.

163 Vindigni SMN, Riley PL, Kimani F, Willy R, Warutere P, Sabatier JF, Kiriinya R, Friedman M, Osumba M, Waudo AN, Rakuom C, Rogers M. Kenya's emergency-hire nursing programme: a pilot evaluation of health service delivery in two districts. Hum Resour Health. 2014;12:16.

164 Vujicic M, Weber SE, Nikolic IA, Atun R, Kumar R. An analysis of GAVI, the Global Fund and World Bank support for human resources for health in developing countries. Health Policy Plan 201227(8):649-657.

165 Wang S, Xu J, Jiang X, Li C, Li H, Song S, Huang E, Meng Q. Trends in health resource disparities in primary health care institutions in Liaoning Province in Northeast China. Int J Equity Health 2018;17(1):178.

166 Wang X, Yang H, Duan Z, Pan J. Spatial accessibility of primary health care in China: A case study in Sichuan Province. Soc Sci Med 2018;209:14-24.

167 Wang Y, Li Y, Qin S, Kong Y, Yu X, Guo K, Meng J. The disequilibrium in the distribution of the primary health workforce among eight economic regions and between rural and urban areas in China. Int J Equity Health 2020;19(1):28.

168 Wei L, Li X, Shang W, Xing X, Yin M, Ling J, Mao K, Zhu Y, Yang K. Tracking rural health service quality and evaluation mechanism: a bibliometric analysis. Chinese Journal of Evidence-Based Medicine. 2017;17(2):221-227.

169 Williams EN, McMeeken JM. Building capacity in the rural physiotherapy workforce: a paediatric training partnership. Rural Remote Health 2014;14:2465.

170 Wiseman V, Lagarde M, Batura N, Lin S, Irava W. Roberts G. Measuring inequalities in the distribution of the Fiji Health Workforce. Int J Equity Health. 2017;16(1):115.

171 Wong HT, Guo YQ, Chiu MYL, Chen S, Zhao Y. Spatial illustration of health-care workforce accessibility index in China: How far has our 2009 health-care reform brought us? Aust J Rural Health 2016;24(1):54-60.

172 Wu HC, Tseng MH Evaluating disparities in elderly community care resources: Using a geographic accessibility and inequality index. Int J Environ Res Public Healht. 2018;15(7):1353.

173 Xu K, Zhang K, Wang D, Zhou L. Trend in distribution of primary health care professionals in Jiangsu province of eastern China. Int J Equity Health. 2014;13:117.

174 Yang L, Wang H, Xue L. What about the health workforce distribution in rural China? An assessment based on eight-year data. Rural Remote Health 2019;19(3):4978.

175 Yang L, Yin Y, Wang H. Is the health workforce distribution in Beijing, China perfectly equitable? Ethiopian Journal of Health Development 2019;33(1):22-27.

176 Zeng W, Tao W, Yan L, Wen J. The health resource allocation and equity of pediatric care in Sichuan province: a cross-sectional study. Chinese Journal of Evidence-based Medicine. 2018;3:267-270.

177 Zhang D, Son H, Shen Y, Chen Z, Rajbhandari-Thapa J, Li Y, Eom H, Bu D, Mu L, Li G, Pagan JA. Assessment of changes in rural and urban primary care workforce in the united states from 2009 to 2017. JAMA Netw Open. 2020;3(10):e2022914.

178 Zhou K, Zhang X, Ding Y, Wang D, Lu Z, Yu M. Inequality trends of health workforce in different stages of medical system reform (1985-2011) in China. Hum Resour Health. 2015;13:94.

179 Zhu A, Tang S, Hoai Thu NT, Supheap L, Liu X. Analysis of strategies to attract and retain rural health workers in Cambodia, China, and Vietnam and context influencing their outcomes. Hum Resour Health. 2019 17(1):2.

180 Zhu B, Fu Y, Liu J, He R, Zhang N, Mao Y. Detecting the priority areas for health workforce allocation with LISA functions: an empirical analysis for China. BMC Health Serv Res. 2018;18(1):957.

181 Zubieta L, Bequet SAF. Factors of primary care demand: a case study. Rural Remote Health. 2010;10(4):1520.
