# Supplementary material for: Characteristics of Medical Deserts and Approaches to Mitigate Their Health Workforce Issues: A Scoping Review of Empirical Studies in Western Countries
Source: Int J Health Policy Manag. 2023 Aug 15;12:7454. doi: 10.34172/ijhpm.2023.7454 (PMC10590222; doi:10.34172/ijhpm.2023.7454)
Supplement: Supplementary file 4 — Key Characteristics of the Included Studies. [file ijhpm-12-7454-s004.pdf]

Article title: Characteristics of Medical Deserts and Approaches to Mitigate Their Health Workforce Issues: A Scoping Review of Empirical Studies in Western Countries

Journal name: International Journal of Health Policy and Management (IJHPM)

Authors' information: Linda E. Flinterman[1]*¶, Ana I. González-González[2]¶, Laura Seils[2], Julia Bes[1], Marta Ballester[2], Joaquim Bañeres[2], Sorin Dan[3], Alicja Domagala[4], Katarzyna Dubas-Jakóbczyk[5], Robert Likic[6], Marieke Kroezen[7], Ronald Batenburg[1,8]

[1]Health Workforce and Organization Studies, Netherlands Institute for Health Services Research (NIVEL), Utrecht, The Netherlands.

[2]Avedis Donabedian Research Institute – UAB, Madrid, Spain.

[3]Innovation and Entrepreneurship InnoLab, University of Vaasa, Vaasa, Finland.

[4]Department of Health Policy and Management, Institute of Public Health, Jagiellonian University, Krakow, Poland.

[5]Department of Health Economics and Social Security, Institute of Public Health, Jagiellonian University, Krakow, Poland.

[6]School of Medicine, University of Zagreb, Zagreb, Croatia.

[7]Trimbos Institute, Netherlands Institute of Mental Health and Addiction, Utrecht, The Netherlands.

[8]Department of Sociology, Radboud University, Nijmegen, The Netherlands.

¶ Both authors contributed equally to this paper.

(*Corresponding author: Email: l.flinterman@nivel.nl)

Citation: Flinterman LE, González-González AI, Seils L, et al. Characteristics of medical deserts and approaches to mitigate their health workforce issues: a scoping review of empirical studies in Western countries. Int J Health Policy Manag. 2023;12:7454. doi:10.34172/ijhpm.2023.7454

Supplementary file 4. Key Characteristics of the Included Studies

Key characteristics of the included studies (n = 240)

First author (reference)	Year	Country	Design	Data collection method	Type of HWF	Participants, n	Medical desert	Outcome type
Abbiati[84]	2020	Switzerland	Quantitative, cross-sectional	Survey	Students (Medical students)	1,749	Underserved areas	Definition & contributing factors
Abid[140]	2020	New Zealand	Quantitative, longitudinal	Questionnaire	Students (Medical students)	1,367	Rural areas	Definition & approaches
Alexander[70]	1998	Australia	Quantitative,	Questionnaire	Physicians (GPs)	104	Rural areas	Contributing factors

First author (reference)	Year	Country	Design	Data collection method	Type of HWF	Participants, n	Medical desert	Outcome type
			cross-sectional					
Allan[81]	2007	Australia	Qualitative	Interviews	Allied HWF (Pharmacists & social workers)	11	Rural areas	Contributing factors
Ariste[94]	2019	Canada	Quantitative, cross-sectional	Existing database	Combination of HWF (Physicians & nurses)	na	Rural areas	Definition & characteristics
Australian Medical Workforce Advisory Committee (AMWAC)[182]	2005	Australia	Quantitative, cross-sectional	Survey	Physicians (Medical graduates)	7,899	Rural areas	Definition & contributing factors
Bailey[203]	2015	Australia	Quantitative, longitudinal	Existing database	Physicians (GPs)	1,154	Rural areas	Definition & contributing factors
Bamford[130]	1999	Australia	Quantitative, cross-sectional	Existing database	Physicians (GPs)	na	Rural areas	Definition & characteristics
Bath[105]	2015	Canada	Quantitative, cross-sectional	Existing database	Allied HWF (Physiotherapists)	643	Rural areas	Definition & characteristics
Beauchamp[183]	2013	Canada	Quantitative, cross-sectional	Questionnaire	Physicians (both family and specialty physicians)	264	Rural areas	Definition & contributing factors
Bennet[82]	2013	Australia	Mixed methods	Questionnaire, interviews & focus groups	Students (Nursing students)	31	Rural & remote areas	Approaches
Bent[83]	1999	Australia	Qualitative	Interviews	Allied HWF (Occupational therapists, physiotherapists, speech pathologists)	17	Remote areas	Contributing factors
Berk[85]	1983	United States	Quantitative, cross-sectional	Survey	Physicians (not defined)	na	Underserved areas	Definition & characteristics
Bigbee[151]	2013	United States	Quantitative, longitudinal	Survey	Students (Nursing students)	1,283	Rural areas	Definition & contributing factors

First author (reference)	Year	Country	Design	Data collection method	Type of HWF	Participants, n	Medical desert	Outcome type
Booza[16]	2010	United States	Quantitative, longitudinal	Existing database	Students (Medical students)	28	Rural areas	Contributing factors & approaches
Boscardin[147]	2014	United States	Quantitative, cross-sectional	Questionnaire	Students (Medical students)	7,631	Underserved areas	Definition, contributing factors & approaches
Bowman[86]	2008	United States	Quantitative, cross-sectional	Existing database	Combination of HWF (Physicians (internal medicine, pediatric and family practice) & nurses)	na	Rural areas	Definition & approaches
Bradley[87]	2018	United States	Mixed methods	Questionnaire	Combination of HWF (Physicians (all types) & nurses)	138	Underserved areas	Definition & approaches
Brockwell[215]	2009	Australia	Quantitative, cross-sectional	Questionnaire	Allied HWF (Occupational therapists)	15	Rural areas	Definition, contributing factors & approaches
Burnett[116]	1995	United States	Quantitative, cross-sectional	Questionnaire	Physicians (Family practice, GP, obstetrics an d gynecology, internal medicine, pediatrics)	5,208	Underserved areas	Definition, characteristics & approaches
Bushy[124]	2005	Canada & United States	Mixed methods	Questionnaire	Students (Nursing students)	44	Rural areas	Definition, characteristics contributing factors
Butler[125]	1999	Australia	Qualitative	Questionnaire	Allied HWF (Physiotherapists)	103	Rural areas	Definition, characteristics & approaches
Butler[152]	2010	Australia	Quantitative, cross-sectional	Existing database	Combination of HWF (Medical workforce in general)	na	Remote areas	Definition, characteristics
Cameron[172]	2010	Canada	Qualitative	Interviews	Physicians (not defined)	41	Rural & remote areas	Definition, characteristics & contributing factors
Carter[126]	1987	Canada	Quantitative, cross-sectional	Questionnaire	Physicians (not defined)	562	Rural areas	Definition, characteristics & contributing factors
Ceronsky[138]	2013	United States	Quantitative, cross-sectional	Questionnaire	Combination of HWF (Rural community teams)	na	Rural areas	Definition, characteristics & approaches

First author (reference)	Year	Country	Design	Data collection method	Type of HWF	Participants, n	Medical desert	Outcome type
Chan[127]	1987	Canada	Quantitative, cross-sectional	Questionnaire	Physicians (General Practitioners)	651	Rural areas	Definition, characteristics & contributing factors
Chen[128]	2013	United States	Quantitative, cross-sectional	Existing database	Physicians (not defined)	759	Rural areas	Definition & characteristics
Chisholm[167]	2011	Australia	Quantitative, cross-sectional	Survey	Combination of HWF (Dietitian, occupational therapists, physiotherapists, podiatrists, psychologists, social worker, speech pathologists)	901	Rural areas	Definition, characteristics & contributing factors
Clark[206]	2013	Australia	Quantitative, longitudinal	Questionnaire	Students (Medical students)	448	Rural areas	Definition & approaches
Courtney[240]	2005	United States	Quantitative, cross-sectional	Existing database	Nurses	na	Underserved areas	Definition & characteristics
Cramer[129]	2006	United States	Quantitative, cross-sectional	Existing database	Nurses	na	Rural areas	Definition & characteristics
Crump[17]	2016	United States	Quantitative, cross-sectional	Existing database	Students (Medical students)	1,120	Rural areas	Approaches
Daniels[18]	2007	United States	Quantitative, cross-sectional	Survey	Students (Health science)	1,396	Rural areas	Contributing factors
Denz-Penhey[19]	2005	Australia	Qualitative	Interviews & questionnaire (Open questions)	Students (Medical students)	na	Rural areas	Approaches
Doogan[131]	2018	United States	Quantitative, cross-sectional	Existing database	Combination of HWF (not defined)	na	Rural areas	Definition & characteristics
Drovandi[197]	2020	Australia	Quantitative, cross-sectional	Existing database	Students (Pharmacy students)	973	Rural areas	Definition & approaches
Duma[148]	2011	Romania	Qualitative	Existing database	Combination of HWF (not defined)	na	Rural areas	Definition & contributing factors
Easterbrook[95]	1999	Canada	Quantitative, cross-sectional	Questionnaire	Physicians (Family medicine)	230	Rural areas	Definition, characteristics & contributing factors

First author (reference)	Year	Country	Design	Data collection method	Type of HWF	Participants, n	Medical desert	Outcome type
Eley[20]	2006	Australia	Quantitative, cross-sectional	Survey	Students (Medical students)	25	Rural areas	Approaches
Eley[209]	2009	Australia	Quantitative, longitudinal	Survey	Students (Medical students)	180	Rural & remote areas	Approaches
Eley[21]	2009	Australia	Quantitative, cross-sectional	Survey	Students (Medical students)	476	Rural areas	Definition & approaches
Eley[175]	2012	Australia	Mixed methods, longitudinal	Questionnaire & interviews	Students (Medical students)	115	Rural areas	Definition & contributing factors
Elliott-Schmidt[22]	1995	United States	Quantitative, cross-sectional	Survey	Allied HWF (Occupational therapists)	30	Rural areas	Contributing factors
Emery[216]	2009	Australia	Quantitative, longitudinal	Existing database	Students (Medical students)	na	Rural areas	Definition & approaches
Feng[241]	2018	United States	Quantitative, cross-sectional	Existing database	Physicians (Dermatologists)	na	Rural areas	Definition & characteristics
Feng[88]	2017	United States	Quantitative, cross-sectional	Existing database	Allied HWF (Dentists)	na	Geographical areas	Definition & characteristics
Florence[96]	2007	United States	Quantitative, cross-sectional	Questionnaire	Students (Health science)	122	Rural areas	Definition, characteristics & approaches
Frisch[97]	2003	United States	Quantitative, longitudinal	Survey	Physicians (GPs)	514	Rural areas	Definition, characteristics & contributing factors
Fryer[242]	1983	United States	Quantitative, cross-sectional	Existing database	Allied HWF (Dentists)	na	Rural areas	Definition & characteristics
Fryer[89]	1999	United States	Quantitative, cross-sectional	Existing database	Physicians (GPs)	na	Rural areas	Definition & characteristics
Garcia[149]	2018	United States	Quantitative, cross-sectional	Questionnaire	Students (Medical students)	40,846	Underserved areas	Definition & contributing factors
Gemelas[243]	2020	United States	Quantitative, cross-sectional	Existing database	Physicians (GPs)	25,104	Rural areas	Definition
Gillham[23]	2013	Australia	Qualitative	Interviews	Students (Allied HWF)	8	Rural areas	Contributing factors
Gilliland[132]	2019	Canada	Quantitative, cross-sectional	Existing database	Physicians (GPs)	na	Underserved areas	Definition & characteristics
Godwin[244]	2017	Australia	Quantitative,	Survey	Allied HWF	631	Rural areas	Definition &

First author (reference)	Year	Country	Design	Data collection method	Type of HWF	Participants, n	Medical desert	Outcome type
			cross-sectional		(Dentists)			characteristics
Goetz[245]	2013	Germany	Quantitative, cross-sectional	Survey	Physicians (GPs)	1,027	Rural areas	Contributing factors
Gray[25]	2009	Australia	Quasi-experimental	Questionnaire	Physicians (Geriatricians)	na	Rural areas	Approaches
Griffin[139]	2016	Australia	Quantitative, cross-sectional	Interviews	Students (Medical students)	351	Underserved areas	Definition, characteristics & contributing factors
Gupta[205]	2019	Australia	Quantitative, longitudinal	Survey	Students (Medical students)	468	Rural areas	Definition & approaches
Hajat[224]	2003	United States	Quantitative, cross-sectional	Survey	Institute/practice (Local public health agencies)	1,100	Rural areas	Definition, characteristics & approaches
Hancock[98]	2009	United States	Qualitative	Interviews & questionnaires	Physicians (GPs)	na	Rural areas	Definition & contributing factors
Hanson[99]	1990	United States	Quantitative, cross-sectional	Questionnaire	Nurses	167	Rural areas	Definition, characteristics, contributing factors & approaches
Hansroth[246]	2021	United States	Quantitative, cross-sectional	Survey	Physicians (emergency department)	29	Rural areas	Definition & characteristics
Hays[162]	1997	Australia	Qualitative	Interviews	Physicians (GPs)	59	Rural areas	Definition & contributing factors
Hays[27]	2003	Australia	Qualitative	Interviews	Physicians (GPs)	23	Rural areas	Contributing factors
Heath[28]	2009	United States	Observational, longitudinal	Questionnaire	Physicians (Paediatricians)	na	Rural areas	Approaches
Henderson[100]	2004	United States	Mixed methods	Questionnaire	Nurses	124	Rural areas	Definition, characteristics & contributing factors
Henning-Smith[101]	2016	United States	Quantitative, cross-sectional	Existing database	Combination of HWF (Child care workers)	na	Rural areas	Definition
Herd[185]	2016	Australia	Quantitative, cross-sectional	Existing database	Students (Medical students)	na	Rural areas	Definition, characteristics & contributing factors
Hills[160]	2011	Australia	Quantitative, cross-sectional	Survey	Physicians	9,900	Rural areas	Definition, characteristics, contributing factors

First author (reference)	Year	Country	Design	Data collection method	Type of HWF	Participants, n	Medical desert	Outcome type
Hoffmann[165]	2015	Austria	Quantitative, cross-sectional	Questionnaire	Physicians (GPs)	173	Rural areas	Definition, characteristics & contributing factors
Hogenbirk[186]	2015	Canada	Quantitative, longitudinal	Questionnaire	Students (Medical students)	na	Rural areas	Definition & contributing factors
Hogue[102]	2019	United States	Quantitative, cross-sectional	Survey	Physicians (GPs)	99	Rural areas	Definition, characteristics & contributing factors
Horner[153]	1993	United States	Quantitative, cross-sectional	Existing database	Physicians (GPs)	1,947	Rural areas	Definition, characteristics & contributing factors
Huicho[238]	2010	Switzerland	Quantitative, cross-sectional	Existing database	Combination of HWF (not defined)	na	Underserved areas	Definition & characteristics
Humphreys[154]	1998	Australia	Quantitative, cross-sectional	Survey	Combination of HWF (not defined)	na	Rural areas	Definition & characteristics
Humphreys[163]	2002	Australia	Quantitative, cross-sectional	Questionnaire	Physicians (GPs)	1,344	Rural & remote areas	Definition & contributing factors
Humphreys[171]	2003	Australia	Quantitative, cross-sectional	Questionnaire	Physicians (GPs)	1,498	Rural & remote areas	Definition & contributing factors
Humphreys[228]	2012	Australia	Quantitative, cross-sectional	Existing database	Physicians (GPs)	3,636	Rural areas	Definition, characteristics & approaches
Irby[230]	2012	United States	Quantitative, longitudinal	Existing database	Physicians (Paediatricians)	na	Rural areas	Definition, characteristics & approaches
Isaac[29]	2014	Australia	Quantitative, longitudinal	Questionnaire	Students (Medical students)	165	Rural areas	Contributing factors & approaches
Iversen[247]	2002	United Kingdom	Qualitative	Interviews	Combination of HWF (GPs, practice nurses, practice managers and administrative staff)	53	Rural areas	Definition, characteristics & contributing factors
Jamar[217]	2014	Australia	Mixed methods	Survey	Students (Medical students)	74	Rural areas	Definition, characteristics & approaches
Janes[166]	2004	New Zealand	Mixed methods	Questionnaire	Physicians (GPs)	559	Rural areas	Definition & contributing factors
Jo[133]	2020	United Kingdom	Quantitative, cross-sectional	Existing database	Allied HWF (Dentists)	1,695	Rural areas	Definition & characteristics
Johnston[248]	2020	Canada	Quantitative, cross-sectional	Existing database	Institute/practice	na	Rural areas	Definition

First author (reference)	Year	Country	Design	Data collection method	Type of HWF	Participants, n	Medical desert	Outcome type
Jones[225]	2004	Australia	Quantitative, cross-sectional	Questionnaire	Physicians (GPs)	1,050	Rural areas	Definition, characteristics & approaches
Jones[30]	2009	Australia & New Zealand	Quantitative, longitudinal	Survey	Students (Medical students)	6,292	Rural areas	Contributing factors
Jones[103]	2014	Australia	Quantitative, cross-sectional	Questionnaire	Students (Medical students)	3,268	Rural areas	Definition, characteristics & contributing factors
Joyce[249]	2005	Australia	Quantitative, cross-sectional	Existing database	Physicians (GPs)	na	Remote areas	Definition & characteristics
Kamien[31]	1998	Australia	Quantitative, cross-sectional	Interviews	Physicians	101	Rural areas	Contributing factors
Kane[213]	2013	United States	Quantitative, longitudinal	Existing database	Students (Medical students)	296	Rural areas	Definition & approaches
Kaye[90]	2006	United States	Quantitative, cross-sectional	Existing database	Allied HWF	na	Underserved areas	Definition & characteristics
Keane[174]	2011	Australia	Qualitative	Survey	Allied HWF	1,879	Rural & remote areas	Definition
Kelley[32]	2004	Canada	Qualitative	Survey	Combination of HWF	296	Rural areas	Approaches
Kent[104]	2018	New Zealand	Quantitative, cross-sectional	Survey	Students (Medical students)	2,243	Rural areas	Definition, characteristics & contributing factors
King[177]	2016	Australia	Quantitative, cross-sectional	Survey	Students (Medical students)	454	Rural areas	Definition & contributing factors
Kippenbrock[250]	2013	United States	Quantitative	Survey	Nurses	na	Rural areas	Definition & characteristics
Kondalsamy-Chennakesavan[184]	2015	Australia	Quantitative, longitudinal	Questionnaire	Students (Medical students)	754	Rural areas	Definition & contributing factors
Kuhn[229]	2017	Germany	Quantitative, cross-sectional	Survey	Combination of HWF	449	Rural areas	Definition, characteristics & approaches
Kuipers[251]	2015	Australia	Quantitative, cross-sectional	Existing database & semi-structured interviews	Allied HWF	41	Rural areas	Definition & characteristics
Kulig[155]	2008	Canada	Quantitative, cross-sectional	Survey	Nurses	3,412	Rural areas	Definition & characteristics
Kwan[187]	2017	Australia	Quantitative, cross-sectional	Survey	Physicians	729	Rural areas	Definition & contributing factors

First author (reference)	Year	Country	Design	Data collection method	Type of HWF	Participants, n	Medical desert	Outcome type
Landry[33]	2011	Canada	Quantitative, cross-sectional	Questionnaire	Physicians	263	Rural areas	Definition & contributing factors
Larson[252]	2003	United States	Quantitative, cross-sectional	Existing database	Combination of HWF	15,606	Rural areas	Definition & characteristics
Lasala[34]	2000	United States	Mixed methods	Questionnaire	Nurses	131	Rural areas	Contributing factors
Laskowska[253]	2015	Poland	Quantitative, cross-sectional	Existing database	Institute/practice	na	Rural areas	Definition & characteristics
Laurence[164]	2010	Australia	Qualitative	Interviews	Physicians (GPs)	na	Rural areas	Definition & contributing factors
Lavanchy[35]	2003	United States	Quantitative, cross-sectional	Questionnaire	Physicians	198	Rural areas	Contributing factors
Lavergne[134]	2016	Canada	Quantitative	Existing database	Institute/practice	89	Rural areas	Definition & characteristics
Lawrence[36]	2015	United States	Quasi-experimental	Survey	Institute/practice	96	Underserved areas	Approaches
Lee[168]	2003	Australia	Qualitative	Interviews	Allied HWF (Occupational therapists)	5	Rural areas	Definition & contributing factors
Lee[38]	2011	Australia	Mixed methods	Survey	Students (Medical students)	88	Rural areas	Contributing factors & approaches
Lin[39]	2006	Australia	Mixed methods, longitudinal	Focus groups & audit	Allied HWF	19	Rural areas	Approaches
Lindeke[40]	2005	United States	Quantitative, cross-sectional	Survey	Nurses	834	Rural areas	Contributing factors
Longenecker[222]	2020	United States	Quantitative, cross-sectional	Existing database	Institute/practice	182	Rural areas	Definition & approaches
Lynge[91]	2008	United States	Quantitative, longitudinal	Existing database	Physicians (Surgeons)	na	Rural areas	Definition & characteristics
MacIsaac[41]	2000	Australia	Qualitative	Interviews	Physicians (GPs)	20	Rural areas	Contributing factors
Magnus[42]	1993	Norway	Quantitative, cross-sectional	Survey	Students (Medical students)	417	Rural areas	Approaches
Mathews[106]	2008	Canada	Quantitative, cross-sectional	Existing database	Students (Medical students)	na	Rural areas	Definition & contributing factors

First author (reference)	Year	Country	Design	Data collection method	Type of HWF	Participants, n	Medical desert	Outcome type
Mathews[254]	2012	Canada	Qualitative	Interviews	Physicians (both family and specialty physicians)	48	Rural areas	Definition, characteristics & contributing factors
Mathews[255]	2015	Canada	Quantitative, cross-sectional	Existing database	Students (Medical students)	1,864	Rural areas	Contributing factors
Matthews[43]	2015	New Zealand	Qualitative	Survey	Students (Medical students)	45	Rural areas	Definition & approaches
May[210]	2018	Australia	Quantitative, cross-sectional	Existing database	Students (Medical students)	426	Rural areas	Definition & approaches
McGirr[156]	2019	Australia	Quantitative, cross-sectional	Survey	Students (Medical students)	na	Rural areas	Definition, contributing factors & approaches
McGrail[256]	2009	Australia	Quantitative, cross-sectional	Existing database	Combination of HWF	na	Rural areas	Definition
McGrail[108]	2011	Australia	Quantitative, cross-sectional	Existing database	Physicians (GPs & specialists)	5,581	Rural & remote areas	Definition & contributing factors
McGrail[109]	2012	Australia	Quantitative, cross-sectional	Survey	Physicians (GPs)	3,906	Rural areas	Definition & characteristics
McGrail[180]	2015	Australia	Quantitative, cross-sectional	Existing database	Combination of HWF	na	Rural & remote areas	Definition & characteristics
McGrail[223]	2016	Australia	Quantitative, longitudinal	Existing database	Physicians (GPs)	610	Rural areas	Definition, contributing factors & approaches
McGrail[157]	2017	Australia and United States	Quantitative, cross-sectional	Existing database	Combination of HWF (community amenities)	na	Rural areas	Definition & contributing factors
McGrail[158]	2018	Australia	Quantitative, cross-sectional	Survey	Physicians (GPs)	960	Underserved areas	Definition, characteristics & approaches
Mills[44]	2002	Australia	Qualitative	Interviews	Allied HWF (Occupational therapists)	10	Rural areas	Contributing factors
Montour[257]	2009	Canada	Qualitative	Interviews	Nurses	13	Rural areas	Definition & characteristics
Morris[45]	2008	United States	Quantitative, cross-sectional	Survey	Physicians (GPs)	838	Rural & underserved areas	Approaches
Natanzon[46]	2010	Germany	Qualitative	Interviews	Physicians (GPs)	16	Rural areas	Contributing factors

First author (reference)	Year	Country	Design	Data collection method	Type of HWF	Participants, n	Medical desert	Outcome type
Newman[47]	2008	Australia	Quantitative, cross-sectional	Survey	Nurses	56	Rural areas	Approaches
Nugent[258]	2004	Australia	Quantitative, cross-sectional	Existing database & survey	Undergraduates (Nurses)	na	Rural areas	Definition & characteristics
O'Connell[150]	2018	United States	Quantitative, longitudinal	Survey	Students (Medical students)	474	Underserved areas	Definition & contributing factors
O'Connor[110]	2006	Australia	Qualitative	Interviews	Physicians (GPs)	10	Rural areas	Definition, characteristics & contributing factors
Orpin[49]	2005	Australia	Quantitative, cross-sectional	Survey	Students (Medical, nursing & pharmacy students)	235	Rural areas	Approaches
Orzanco[111]	2011	Canada	Quantitative, longitudinal	Existing database	Students (Medical students)	180	Non-metropolitan areas	Definition, characteristics & contributing factors
O'Sullivan[259]	2018	Australia	Quantitative, longitudinal	Existing database	Students (Medical students)	2,412	Rural areas	Definition & contributing factors
O'Toole[51]	2008	Australia	Quantitative, cross-sectional	Survey	Allied HWF (Clinical worker, team leader, manager, educational worker, casework coordinator)	138	Rural areas	Contributing factors
O'Toole[50]	2010	Australia	Mixed methods	Interviews	Allied HWF (Social worker, dietitian, physiotherapist, speech pathologist, occupational therapist, health promoter, radiologist, podiatrist)	32	Rural areas	Contributing factors
Pathman[141]	1992	United States	Quantitative, longitudinal	Survey	Physicians (Primary care)	412	Rural areas	Definition, characteristics, contributing factors & approaches
Pathman[142]	1999	United States	Quantitative, cross-sectional	Survey	Physicians (Primary care)	456	Rural areas	Definition, contributing factors & approaches
Pathman[112]	2004	United States	Quantitative, cross-sectional	Questionnaire	Physicians (not defined)	45	Underserved areas	Definition & contributing factors

First author (reference)	Year	Country	Design	Data collection method	Type of HWF	Participants, n	Medical desert	Outcome type
Pearce[260]	2008	New Zealand	Quantitative, cross-sectional	Existing database	Institute/practice (Community resource access)	na	Rural & remote areas	Definition & characteristics
Pearce[135]	2006	New Zealand	Quantitative, cross-sectional	Existing database	Institute/practice (Community resource access)	na	Remote areas	Definition & characteristics
Pegram[143]	2006	Australia	Quantitative, cross-sectional	Existing database	Physicians (GPs)	na	Rural areas	Definition & characteristics
Pepper[52]	2010	United States	Quantitative, cross-sectional	Survey	Physicians (Medical doctors & osteopaths)	693	Rural areas	Contributing factors
Perkins[53]	2007	Australia	Mixed methods	Interviews	Combination of HWF (Members of mental health teams: managers, team leaders & members, interns)	41	Rural areas	Contributing factors
Playford[54]	2006	Australia	Quantitative, longitudinal	Survey	Students (Health science)	429	Rural areas	Contributing factors
Playford[55]	2010	Australia	Quantitative, longitudinal	Interviews	Students (Nursing students)	49	Rural areas	Approaches
Playford[56]	2012	Australia	Quantitative, longitudinal	Existing database	Students (Medical students)	na	Rural areas	Contributing factors & approaches
Playford[211]	2014	Australia	Quantitative, longitudinal	Existing database	Students (Medical students)	1,017	Rural areas	Definition & approaches
Playford[199]	2016	Australia	Quantitative, longitudinal	Survey	Students (Medical students)	417	Rural areas	Definition, contributing factors & approaches
Playford[188]	2017	Australia	Quantitative, longitudinal	Existing database	Students (Medical students)	508	Rural areas	Definition & contributing factors
Playford[136]	2019	Australia	Quantitative, longitudinal	Existing database	Students (Medical students)	1,122	Rural areas	Definition, contributing factors & approaches
Puddey[181]	2014	Australia	Quantitative, longitudinal	Questionnaire	Students (Medical students)	538	Rural areas	Definition & contributing factors
Puddey[113]	2015	Australia	Quantitative, longitudinal	Questionnaire	Students (Medical students)	729	Rural areas	Definition & contributing factors

First author (reference)	Year	Country	Design	Data collection method	Type of HWF	Participants, n	Medical desert	Outcome type
Rabinowitz[218]	1988	United States	Quantitative, longitudinal	Existing database	Physicians (GPs)	135	Rural areas	Definition & approaches
Rabinowitz[219]	1993	United States	Quantitative, longitudinal	Existing database	Students (Medical students)	47	Rural areas	Definition & approaches
Rabinowitz[221]	1999	United States	Quantitative, longitudinal	Existing database	Physicians (GPs)	150	Rural areas	Definition & approaches
Rabinowitz[173]	1999	United States	Quantitative, cross-sectional	Existing database	Physicians (Medical graduates)	1,609	Rural areas	Definition & contributing factors
Rabinowitz[191]	2000	United States	Quantitative, cross-sectional	Questionnaire	Physicians (GPs)	1,704	Rural areas	Definition & contributing factors
Rabinowitz[190]	2001	United States	Quantitative, longitudinal	Existing database	Students (Nursing students)	3,414	Rural areas	Definition, contributing factors & approaches
Rabinowitz[57]	2005	United States	Quantitative, longitudinal	Existing database	Physicians (GPs)	na	Rural areas	Contributing factors & approaches
Rabinowitz[189]	2012	United States	Quantitative, longitudinal	Existing database	Students (Medical students)	762	Rural areas	Definition & contributing factors
Rabinowitz[220]	2013	United States	Quantitative, longitudinal	Existing database	Physicians (Family medicine)	89	Rural areas	Definition & approaches
Ramos[58]	2014	United States	Quantitative, cross-sectional	Survey	Nurses	360	Rural areas	Approaches
Ray[227]	2014	Australia	Mixed methods	Survey	Combination of HWF (Primary care)	174	Rural areas	Definition, characteristics & approaches
Reymond[60]	2005	Australia	Mixed methods	Questionnaire	Combination of HWF (Primary care)	na	Rural areas	Approaches
Ricketts[14]	1996	United states	Quantitative, cross-sectional	Existing database	Physicians (Obstetrician-gynaecologists)	na	Rural areas	Contributing factors
Rohova[144]	2017	Bulgaria	Quantitative	Existing database	Combination of HWF (all types of physicians, GPs, dentists, nursing professionals, nurses)	na	Underserved areas	Definition & characteristics
Rolfe[161]	1995	Australia	Quantitative, cross-sectional	Questionnaire	Physicians Medical graduates of all types	331	Rural areas	Definition & contributing factors

First author (reference)	Year	Country	Design	Data collection method	Type of HWF	Participants, n	Medical desert	Outcome type
Roots[114]	2014	Canada	Qualitative	Interviews	Allied HWF (Occupational therapists, physical therapists)	19	Rural areas	Definition, characteristics & approaches
Rosenblatt[200]	1992	United States	Quantitative, longitudinal	Existing database	Physicians (Medical graduates of all types)	15,375	Rural areas	Definition & contributing factors
Rourke[61]	2003	Canada	Quantitative, cross-sectional	Questionnaire	Physicians (Family medicine & residents)	507	Rural areas	Approaches
Rourke[115]	2018	Canada	Quantitative, cross-sectional	Existing database	Physicians (amily medicine graduates)	na	Rural areas	Definition & approaches
Royston[234]	2012	United states	Quantitative, cross-sectional	Existing database & survey	Students (Osteopathic students)	141	Rural areas	Definition & characteristics
Runge[192]	2016	Australia	Quantitative, cross-sectional	Existing database	Physicians (all types)	633	Rural areas	Definition, characteristics & contributing factors
Russell[226]	2013	Australia	Quantitative, cross-sectional	Existing database	Physicians (GPs)	2,783	Rural areas	Definition, contributing factors & approaches
Russell[261]	2013	Australia	Quantitative, cross-sectional	Existing database & survey	Combination of HWF (Primary care)	108	Rural areas	Definition
Sabesan[62]	2012	Australia	Quantitative, cross-sectional	Existing database	Physicians (Oncologists)	na	Rural & remote areas	Approaches
Sen Gupta[201]	2013	Australia	Quantitative, longitudinal	Existing database & survey	Students (Medical students)	292	Rural & remote areas	Definition, contributing factors & approaches
Sen Gupta[193]	2014	Australia	Quantitative, longitudinal	Existing database & survey	Students (Medical students)	536	Rural & remote areas	Definition, contributing factors & approaches
Senn[262]	2016	Switzerland	Quantitative, cross-sectional	Survey	Combination of HWF (Primary care)	60	Rural areas	Definition & characteristics
Shah[263]	2017	Canada	Quantitative, cross-sectional	Existing database	Combination of HWF (GPs, nurse practitioners)	na	Underserved areas	Definition & characteristics
Shires[204]	2015	Australia	Quantitative, longitudinal	Existing database	Students (Medical students)	974	Rural areas	Definition & approaches
Simou[92]	2015	Greece	Quantitative, cross-sectional	Existing database	Combination of HWF (Medical personnel, nursing personnel,	na	Rural areas, islands	Definition

First author (reference)	Year	Country	Design	Data collection method	Type of HWF	Participants, n	Medical desert	Outcome type
					other health care personnel (health visitors, midwives, social workers, physiotherapists, laboratory assistants, operators of medical equipment), administrative personnel, technical personnel)			
Smedts[63]	2008	Australia	Quantitative, longitudinal	Existing database	Students (Medical students)	683	Rural areas/Declining population	Approaches
Smith[145]	2013	United states	Quantitative, cross-sectional	Existing database	Combination of HWF (Hospital)	na	Rural areas	Definition & characteristics
Smith[170]	2018	Australia	Qualitative	Questionnaire	Students (Medical students)	3,204	Rural areas	Definition & contributing factors
Smith[194]	2021	Australia	Quantitative, cross-sectional	Existing databases	Students (Medical students)	1,315	Rural areas	Definition & contributing factors
Somers[202]	2012	Australia	Quasi-experimental	Survey	Students (Medical students)	58	Rural areas	Definition, characteristics, contributing factors & approaches
Spencer[64]	2008	Australia	Quantitative, longitudinal	Questionnaire	Students (Medical students)	43	Rural areas	Contributing factors
Stagg[176]	2009	Australia	Quantitative, longitudinal	Survey	Students (Medical students)	46	Rural areas	Definition & contributing factors
Steenbergen[65]	2004	Australia	Qualitative	Interviews	Allied HWF (Occupational therapists)	9	Rural areas	Approaches
Steinhaeuser[37]	2011	Germany	Quantitative, cross-sectional	Survey	Physicians (GPs)	715	Rural areas	Characteristics & contributing factors
Steinhaeuser[159]	2014	Germany	Quantitative, cross-sectional	Survey	Physicians (GPs)	724	Rural areas	Definition & characteristics
Strasser[264]	2000	Australia	Quantitative, cross-sectional	Existing database & survey	Physicians (GPs)	na	Rural areas	Definition & characteristics

First author (reference)	Year	Country	Design	Data collection method	Type of HWF	Participants, n	Medical desert	Outcome type
Strasser[195]	2010	Australia	Quantitative, longitudinal	Questionnaire	Students (Medical students)	123	Rural areas	Definition & contributing factors
Stratton[66]	1998	United States	Quantitative, cross-sectional	Existing database	Nurses	164	Rural areas	Contributing factors
Sullivan Havens[79]	2013	United states	Quantitative, cross-sectional	Survey	Nurses	747	Rural areas	Characteristics
Szafran[118]	2001	Canada	Quantitative, cross-sectional	Survey	Physicians (GPs)	702	Rural areas	Definition, characteristics & contributing factors
Thackrah[67]	2017	Australia	Qualitative	Interviews	Students (Health science)	12	Rural areas	Approaches
Theodorakis[15]	2005	Greece	Quantitative, cross-sectional	Existing database	Physicians (Primary care)	na	Rural areas	Characteristics
Thomas[119]	2006	United States	Quantitative, cross-sectional	Existing database	Physicians (Psychiatrists)	na	Rural areas	Definition & characteristics
Tolhurst[68]	2006	Australia	Qualitative	Focus groups & interviews	Students (Medical students)	130	Rural areas	Contributing factors
Toomey[93]	2013	Canada	Qualitative	Interviews	Physicians (Community leaders)	25	Underserved areas	Definition & approaches
Tsiouli[48]	2016	Greece	Quantitative, cross-sectional	Existing database	Allied HWF (Orthodontists)	na	Rural areas	Characteristics
Ulmer[59]	2002	Australia	Quantitative, cross-sectional	Questionnaire	Physicians (GPs)	406	Rural areas	Contributing factors
Van Hassel[120]	2018	Netherlands	Quantitative, cross-sectional	Survey	Physicians (GPs)	596	Rural areas/Declining population	Definition, characteristics & contributing factors
Wade[265]	2007	United States	Quantitative, cross-sectional	Existing database	Physicians (GPs)	1,283	Rural areas	Definition, contributing factors
Wainer[169]	2004	Australia	Quantitative, cross-sectional	Survey	Physicians (Female GPs)	302	Rural & remote areas	Definition, contributing factors & approaches
Walker[69]	2012	Australia	Quantitative, cross-sectional	Questionnaire	Students (Medical students)	125	Rural areas	Contributing factors
Wan[266]	2012	United states	Quantitative, cross-sectional	Existing database	Combination of HWF (Primary care)	na	Underserved areas	Definition & characteristics
Ward[71]	2004	Australia	Quantitative, longitudinal	Questionnaire	Students (Medical students)	299	Rural areas	Contributing factors

First author (reference)	Year	Country	Design	Data collection method	Type of HWF	Participants, n	Medical desert	Outcome type
Wayne[72]	2010	United States	Quantitative, cross-sectional	Survey	Students (Medical students)	244	Underserved areas	Contributing factors
Wendling[212]	2016	United States	Quantitative, cross-sectional	Existing database	Physicians (Rural Physician Programs graduates)	2,778	Rural areas	Definition & approaches
White[73]	2021	France	Qualitative	Survey	Combination of HWF (HCW of the Hospital)	213	Rural areas	Approaches
Wilkinson[214]	2003	Australia	Quantitative, cross-sectional	Questionnaire	Physicians (GPs)	2,414	Rural areas	Definition & approaches
Wilkinson[80]	2008	Australia	Quantitative, cross-sectional	Existing database	Physicians (GPs)	26,359	Rural areas	Characteristics
Williams[74]	2007	Australia	Quantitative, cross-sectional	Survey	Allied HWF (Physiotherapists)	107	Rural areas	Contributing factors
Williamson[75]	2003	New Zealand	Quasi-experimental	Questionnaire	Students (Medical students)	167	Rural areas	Contributing factors & approaches
Williamson[76]	2012	New Zealand	Quantitative, longitudinal	Survey	Students (Medical students)	177	Rural areas	Approaches
Willie-Stephens[267]	2014	Australia	Quantitative, cross-sectional	Existing database	Allied HWF (Dentists)	na	Underserved areas	Definition & characteristics
Wilson[77]	2005	United States	Quantitative, longitudinal	Survey	Nurses	35	Rural areas	Approaches
Woloschuk[121]	2002	Canada	Quasi-experimental	Questionnaire	Students (Medical students)	273	Rural areas	Definition, contributing factors & approaches
Wood[122]	1998	United States	Quantitative, longitudinal	Questionnaire	Students (Nursing students)	136	Rural areas	Definition, contributing factors & approaches
Wood[123]	2018	United States	Quantitative, longitudinal	Existing database	Institute/practice (Satellite HIV clinics)	4	Rural & underserved urban areas	Definition, characteristics & approaches
Woolley[196]	2014	Australia	Quantitative, longitudinal	Existing database & survey	Students (Medical students)	264	Outer regional & remote areas	Definition, characteristics & contributing factors
Worley[78]	2008	Australia	Quantitative, cross-sectional	Survey	Students (Medical students)	150	Rural & remote areas	Approaches
Yan[268]	2011	Australia	Quantitative, cross-sectional	Survey	Physicians (All types)	15,871	Rural & remote areas	Definition & characteristics

First author (reference)	Year	Country	Design	Data collection method	Type of HWF	Participants, n	Medical desert	Outcome type
Young[208]	2011	Australia	Quantitative, longitudinal	Survey	Students (Medical students)	688	Rural & remote areas	Definition & approaches
Zink[207]	2010	United States	Quantitative, cross-sectional	Existing database	Students (Medical students)	3,365	Rural areas	Definition, characteristics & approaches
Zhu[146]	2015	United States	Quantitative, cross-sectional	Existing database	Combination of HWF	na	Rural areas	Definition & characteristics

GPs = General Practitioners; HWF = health work force; n = number; na = not available/not applicable

14. Ricketts TC, Tropman SE, Slifkin RT, Konrad TR. Migration of obstetrician-gynecologists into and out of rural areas, 1985-1990. Med Care. 1996;34(5):428-438. doi:10.1097/00005650-199605000-00005

15. Theodorakis PN, Mantzavinis GD. Inequalities in the distribution of rural primary care physicians in two remote neighboring prefectures of Greece and Albania. Rural Remote Health. 2005;5(3):457.

16. Booza JC, Bridge PD, Neale AV, Schenk M. Incorporating Geographic Information Systems (GIS) into program evaluation: lessons from a rural medicine initiative. J Am Board Fam Med. 2010;23(1):59-66. doi:10.3122/jabfm.2010.01.090167

17. Crump WJ, Fricker RS, Ziegler CH, Wiegman DL. Increasing the rural physician workforce: a potential role for small rural medical school campuses. J Rural Health. 2016;32(3):254-259. doi:10.1111/jrh.12156

18. Daniels ZM, Vanleit BJ, Skipper BJ, Sanders ML, Rhyne RL. Factors in recruiting and retaining health professionals for rural practice. J Rural Health. 2007;23(1):62-71. doi:10.1111/j.1748-0361.2006.00069.x

19. Denz-Penhey H, Shannon S, Murdoch CJ, Newbury JW. Do benefits accrue from longer rotations for students in Rural Clinical Schools? Rural Remote Health. 2005;5(2):414.

20. Eley D, Baker P. Does recruitment lead to retention? Rural Clinical School training experiences and subsequent intern choices. Rural Remote Health. 2006;6(1):511.

21. Eley D, Young L, Przybeck TR. Exploring temperament and character traits in medical students; a new approach to increase the rural workforce. Med Teach. 2009;31(3):e79-84. doi:10.1080/01421590802335892

22. Elliott-Schmidt R, Strong J. Rural occupational therapy practice: a survey of rural practice and clinical supervision in rural Queensland and Northern New South Wales. Aust J Rural Health. 1995;3(3):122-131. doi:10.1111/j.1440-1584.1995.tb00164.x

23. Gillham S, Ristevski E. Where do I go from here: we've got enough seniors? Aust J Rural Health. 2007;15(5):313-320. doi:10.1111/j.1440-1584.2007.00900.x

25. Gray LC, Wright OR, Cutler AJ, Scuffham PA, Wootton R. Geriatric ward rounds by video conference: a solution for rural hospitals. Med J Aust. 2009;191(11-12):605-608. doi:10.5694/j.1326-5377.2009.tb03345.x

27. Hays R, Wynd S, Veitch C, Crossland L. Getting the balance right? GPs who chose to stay in rural practice. Aust J Rural Health. 2003;11(4):193-198.

28. Heath B, Salerno R, Hopkins A, Hertzig J, Caputo M. Pediatric critical care telemedicine in rural underserved emergency departments. Pediatr Crit Care Med. 2009;10(5):588-591. doi:10.1097/PCC.0b013e3181a63eac

30. Jones M, Humphreys J, Prideaux D. Predicting medical students' intentions to take up rural practice after graduation. Med Educ. 2009;43(10):1001-1009. doi:10.1111/j.1365-2923.2009.03506.x

31. Kamien M. Staying in or leaving rural practice: 1996 outcomes of rural doctors' 1986 intentions. Med J Aust. 1998;169(6):318-321. doi:10.5694/j.1326-5377.1998.tb140285.x

32. Kelley ML, Habjan S, Aegard J. Building capacity to provide palliative care in rural and remote communities: does education make a difference? J Palliat Care. 2004;20(4):308-315.

33. Landry M, Schofield A, Bordage R, Bélanger M. Improving the recruitment and retention of doctors by training medical students locally. Med Educ. 2011;45(11):1121-1129. doi:10.1111/j.1365-2923.2011.04055.x

34. Lasala K. Nursing workforce issues in rural and urban settings: looking at the difference in recruitment, retention and distribution. Online J Rural Nurs Health Care. 2000;1(1):8-24. doi:10.14574/ojrnhc.v1i1.499

35. Lavanchy M, Connelly I, Grzybowski S, Michalos AC, Berkowitz J, Thommasen HV. Determinants of rural physicians' life and job satisfaction. Soc Indic Res. 2004;69(1):93-101. doi:10.1023/B:SOCI.0000032662.79752.e8

36. Lawrence D, Bryant TK, Nobel TB, Dolansky MA, Singh MK. A comparative evaluation of patient satisfaction outcomes in an interprofessional student-run free clinic. J Interprof Care. 2015;29(5):445-450. doi:10.3109/13561820.2015.1010718

37. Steinhaeuser J, Joos S, Szecsenyi J, Miksch A. A comparison of the workload of rural and urban primary care physicians in Germany: analysis of a questionnaire survey. BMC Fam Pract. 2011;12:112. doi:10.1186/1471-2296-12-112

38. Lee YH, Barnard A, Owen C. Initial evaluation of rural programs at the Australian National University: understanding the effects of rural programs on intentions for rural and remote medical practice. Rural Remote Health. 2011;11(2):1602.

39. Lin IB, Goodale BJ. Improving the supervision of therapy assistants in Western Australia: the Therapy Assistant Project (TAP). Rural Remote Health. 2006;6(1):479.

40. Lindeke L, Jukkala A, Tanner M. Perceived barriers to nurse practitioner practice in rural settings. J Rural Health. 2005;21(2):178-181. doi:10.1111/j.1748-0361.2005.tb00079.x

41. MacIsaac P, Snowdon T, Thompson R, Crossland L, Veitch C. General practitioners leaving rural practice in Western Victoria. Aust J Rural Health. 2000;8(2):68-72. doi:10.1046/j.1440-1584.2000.00232.x

42. Magnus JH, Tollan A. Rural doctor recruitment: does medical education in rural districts recruit doctors to rural areas? Med Educ. 1993;27(3):250-253. doi:10.1111/j.1365-2923.1993.tb00264.x

43. Matthews C, Bagg W, Yielder J, Mogol V, Poole P. Does Pukawakawa (the regional-rural programme at the University of Auckland) influence workforce choice? N Z Med J. 2015;128(1409):35-43.

44. Mills A, Millsteed J. Retention: an unresolved workforce issue affecting rural occupational therapy services. Aust Occup Ther J. 2002;49(4):170-81. doi:10.1046/j.1440-1630.2002.00293.x

45. Morris CG, Johnson B, Kim S, Chen F. Training family physicians in community health centers: a health workforce solution. Fam Med. 2008;40(4):271-276.

46. Natanzon I, Szecsenyi J, Ose D, Joos S. Future potential country doctor: the perspectives of German GPs. Rural Remote Health. 2010;10(2):1347.

47. Newman C, Martin E, McGarry DE, Cashin A. Survey of a videoconference community of professional development for rural and urban nurses. Rural Remote Health. 2009;9(2):1134.

48. Tsiouli K, Karamesinis K, Antonarakis GS, Christou P. Prediction model of regional orthodontic workforce needs, using Greece as an example. Eur J Paediatr Dent. 2016;17(1):29-33.

49. Orpin P, Gabriel M. Recruiting undergraduates to rural practice: what the students can tell us. Rural Remote Health. 2005;5(4):412.

50. O'Toole K, Schoo A, Hernan A. Why did they leave and what can they tell us? Allied health professionals leaving rural settings. Aust Health Rev. 2010;34(1):66-72. doi:10.1071/ah09711

51. O'Toole K, Schoo A, Stagnitti K, Cuss K. Rethinking policies for the retention of allied health professionals in rural areas: a social relations approach. Health Policy. 2008;87(3):326-332. doi:10.1016/j.healthpol.2008.01.012

52. Pepper CM, Sandefer RH, Gray MJ. Recruiting and retaining physicians in very rural areas. J Rural Health. 2010;26(2):196-200. doi:10.1111/j.1748-0361.2010.00282.x

53. Perkins D, Larsen K, Lyle D, Burns P. Securing and retaining a mental health workforce in Far Western New South Wales. Aust J Rural Health. 2007;15(2):94-98. doi:10.1111/j.1440-1584.2007.00860.x

54. Playford D, Larson A, Wheatland B. Going country: rural student placement factors associated with future rural employment in nursing and allied health. Aust J Rural Health. 2006;14(1):14-19. doi:10.1111/j.1440-1584.2006.00745.x

55. Playford D, Wheatland B, Larson A. Does teaching an entire nursing degree rurally have more workforce impact than rural placements? Contemp Nurse. 2010;35(1):68-76. doi:10.5172/conu.2010.35.1.068

56. Playford DE, Cheong E. Rural Undergraduate Support and Coordination, Rural Clinical School, and Rural Australian Medical Undergraduate Scholarship: rural undergraduate initiatives and subsequent rural medical workforce. Aust Health Rev. 2012;36(3):301-307. doi:10.1071/ah11072

57. Rabinowitz HK, Diamond JJ, Markham FW, Rabinowitz C. Long-term retention of graduates from a program to increase the supply of rural family physicians. Acad Med. 2005;80(8):728-732. doi:10.1097/00001888-200508000-00004

58. Ramos MM, Fullerton L, Sapien R, Greenberg C, Bauer-Creegan J. Rural-urban disparities in school nursing: implications for continuing education and rural school health. J Rural Health. 2014;30(3):265-274. doi:10.1111/jrh.12058

59. Ulmer B, Harris M. Australian GPs are satisfied with their job: even more so in rural areas. Fam Pract. 2002;19(3):300-303. doi:10.1093/fampra/19.3.300

60. Reymond L, Charles M, Israel F, Read T, Treston P. A strategy to increase the palliative care capacity of rural primary health care providers. Aust J Rural Health. 2005;13(3):156-161. doi:10.1111/j.1440-1854.2005.00687.x

61. Rourke JT, Incitti F, Rourke LL, Kennard M. Keeping family physicians in rural practice. Solutions favoured by rural physicians and family medicine residents. Can Fam Physician. 2003;49:1142-1149.

62. Sabesan S, Larkins S, Evans R, et al. Telemedicine for rural cancer care in North Queensland: bringing cancer care home. Aust J Rural Health. 2012;20(5):259-264. doi:10.1111/j.1440-1584.2012.01299.x

63. McDonnel Smedts A, Lowe MP. Efficiency of clinical training at the Northern Territory Clinical School: placement length and rate of return for internship. Med J Aust. 2008;189(3):166-168. doi:10.5694/j.1326-5377.2008.tb01953.x

64. Spencer RJ, Cardin AJ, Ranmuthugala G, Somers GT, Solarsh B. Influences on medical students' decisions to study at a rural clinical school. Aust J Rural Health. 2008;16(5):262-268. doi:10.1111/j.1440-1584.2008.00978.x

65. Steenbergen K, Mackenzie L. Professional support in rural New South Wales: perceptions of new graduate occupational therapists. Aust J Rural Health. 2004;12(4):160-165. doi:10.1111/j.1440-1854.2004.00590.x

66. Stratton TD, Dunkin JW, Szigeti E, Muus KJ. Recruitment barriers in rural community hospitals: a comparison of nursing and nonnursing factors. Appl Nurs Res. 1998;11(4):183-189. doi:10.1016/s0897-1897(98)80307-8

67. Thackrah RD, Hall M, Fitzgerald K, Thompson SC. Up close and real: living and learning in a remote community builds students' cultural capabilities and understanding of health disparities. Int J Equity Health. 2017;16(1):119. doi:10.1186/s12939-017-0615-x

68. Tolhurst HM, Adams J, Stewart SM. An exploration of when urban background medical students become interested in rural practice. Rural Remote Health. 2006;6(1):452.

69. Walker JH, Dewitt DE, Pallant JF, Cunningham CE. Rural origin plus a rural clinical school placement is a significant predictor of medical students' intentions to practice rurally: a multi-university study. Rural Remote Health. 2012;12:1908.

70. Alexander C. Why doctors would stay in rural practice in the New England health area of New South Wales. Aust J Rural Health. 1998;6(3):136-139. doi:10.1111/j.1440-1584.1998.tb00299.x

71. Ward AM, Kamien M, Lopez DG. Medical career choice and practice location: early factors predicting course completion, career choice and practice location. Med Educ. 2004;38(3):239-248. doi:10.1046/j.1365-2923.2004.01762.x

72. Wayne SJ, Kalishman S, Jerabek RN, Timm C, Cosgrove E. Early predictors of physicians' practice in medically underserved communities: a 12-year follow-up study of University of New Mexico School of Medicine graduates. Acad Med. 2010;85(10 Suppl):S13-16. doi:10.1097/ACM.0b013e3181ed1bee

73. White CH, Meier N, Swint C. The implementation of a stress management program for health care workers through a rural occupational health clinic. Workplace Health Saf. 2021;69(4):161-167. doi:10.1177/2165079920982406

74. Williams E, D'Amore W, McMeeken J. Physiotherapy in rural and regional Australia. Aust J Rural Health. 2007;15(6):380-386. doi:10.1111/j.1440-1584.2007.00931.x

75. Williamson M, Gormley A, Bills J, Farry P. The new rural health curriculum at Dunedin School of Medicine: how has it influenced the attitudes of medical students to a career in rural general practice? N Z Med J. 2003;116(1179):U537.

76. Williamson MI, Wilson R, McKechnie R, Ross J. Does the positive influence of an undergraduate rural placement persist into postgraduate years? Rural Remote Health. 2012;12:2011.

77. Wilson AA. Impact of management development on nurse retention. Nurs Adm Q. 2005;29(2):137-145. doi:10.1097/00006216-200504000-00008

78. Worley P, Martin A, Prideaux D, Woodman R, Worley E, Lowe M. Vocational career paths of graduate entry medical students at Flinders University: a comparison of rural, remote and tertiary tracks. Med J Aust. 2008;188(3):177-178. doi:10.5694/j.1326-5377.2008.tb01567.x

79. Sullivan Havens D, Warshawsky NE, Vasey J. RN work engagement in generational cohorts: the view from rural US hospitals. J Nurs Manag. 2013;21(7):927-940. doi:10.1111/jonm.12171

80. Wilkinson D. Selected demographic, social and work characteristics of the Australian general medical practitioner workforce: comparing capital cities with regional areas. Aust J Rural Health. 2000;8(6):327-334. doi:10.1046/j.1440-1584.2000.00316.x

81. Allan J, Crockett J, Ball P, Alston M, Whittenbury K. 'It's all part of the package' in rural allied health work: a pilot study of rewards and barriers in rural pharmacy and social work. Internet J Allied Health Sci Pract. 2007;5(3):1-11. doi:10.46743/1540-580x/2007.1161

82. Bennett P, Jones D, Brown J, Barlow V. Supporting rural/remote primary health care placement experiences increases undergraduate nurse confidence. Nurse Educ Today. 2013;33(2):166-172. doi:10.1016/j.nedt.2012.02.015

83. Bent A. Allied health in Central Australia: challenges and rewards in remote area practice. Aust J Physiother. 1999;45(3):203-212. doi:10.1016/s0004-9514(14)60351-2

84. Abbiati M, Savoldelli GL, Baroffio A, Bajwa NM. Motivational factors influencing student intentions to practise in underserved areas. Med Educ. 2020;54(4):356-363. doi:10.1111/medu.14063

85. Berk ML, Bernstein AB, Taylor AK. The use and availability of medical care in health manpower shortage areas. Inquiry. 1983;20(4):369-380.

86. Bowman RC. Measuring primary care: the standard primary care year. Rural Remote Health. 2008;8(3):1009.

87. Bradley KJ, Wros P, Bookman N, et al. The Interprofessional Care Access Network (I-CAN): achieving client health outcomes by addressing social determinants in the community. J Interprof Care. 2018:1-8. doi:10.1080/13561820.2018.1560246

88. Feng X, Sambamoorthi U, Wiener RC. Dental workforce availability and dental services utilization in Appalachia: a geospatial analysis. Community Dent Oral Epidemiol. 2017;45(2):145-152. doi:10.1111/cdoe.12270

89. Fryer GE Jr, Drisko J, Krugman RD, et al. Multi-method assessment of access to primary medical care in rural Colorado. J Rural Health. 1999;15(1):113-121. doi:10.1111/j.1748-0361.1999.tb00605.x

90. Kaye HS, Chapman S, Newcomer RJ, Harrington C. The personal assistance workforce: trends in supply and demand. Health Aff (Millwood). 2006;25(4):1113-1120. doi:10.1377/hlthaff.25.4.1113

91. Lynge DC, Larson EH, Thompson MJ, Rosenblatt RA, Hart LG. A longitudinal analysis of the general surgery workforce in the United States, 1981-2005. Arch Surg. 2008;143(4):345-350. doi:10.1001/archsurg.143.4.345

92. Simou E, Karamagioli E, Roumeliotou A. Reinventing primary health care in the Greece of austerity: the role of health-care workers. Prim Health Care Res Dev. 2015;16(1):5-13. doi:10.1017/s1463423613000431

93. Toomey P, Lovato CY, Hanlon N, Poole G, Bates J. Impact of a regional distributed medical education program on an underserved community: perceptions of community leaders. Acad Med. 2013;88(6):811-818. doi:10.1097/ACM.0b013e318290f9c7

94. Ariste R. Availability of health workforce in urban and rural areas in relation to Canadian seniors. Int J Health Plann Manage. 2019;34(2):510-520. doi:10.1002/hpm.2712

95. Easterbrook M, Godwin M, Wilson R, et al. Rural background and clinical rural rotations during medical training: effect on practice location. CMAJ. 1999;160(8):1159-1163.

96. Florence JA, Goodrow B, Wachs J, Grover S, Olive KE. Rural health professions education at East Tennessee State University: survey of graduates from the first decade of the community partnership program. J Rural Health. 2007;23(1):77-83. doi:10.1111/j.1748-0361.2006.00071.x

97. Frisch L, Kellerman R, Ast T. A cohort study of family practice residency graduates in a predominantly rural state: initial practice site selection and trajectories of practice movement. J Rural Health. 2003;19(1):47-54. doi:10.1111/j.1748-0361.2003.tb00541.x

98. Hancock C, Steinbach A, Nesbitt TS, Adler SR, Auerswald CL. Why doctors choose small towns: a developmental model of rural physician recruitment and retention. Soc Sci Med. 2009;69(9):1368-1376. doi:10.1016/j.socscimed.2009.08.002

99. Hanson CM, Jenkins S, Ryan R. Factors related to job satisfaction and autonomy as correlates of potential job retention for rural nurses. J Rural Health. 1990;6(3):302-316. doi:10.1111/j.1748-0361.1990.tb00669.x

100. Henderson Betkus M, MacLeod ML. Retaining public health nurses in rural British Columbia: the influence of job and community satisfaction. Can J Public Health. 2004;95(1):54-58. doi:10.1007/bf03403635

101. Henning-Smith C, Kozhimannil KB. Availability of child care in rural communities: implications for workforce recruitment and retention. J Community Health. 2016;41(3):488-493. doi:10.1007/s10900-015-0120-3

102. Hogue A, Huntington MK. Family physician burnout rates in rural versus metropolitan areas: a pilot study. S D Med. 2019;72(7):306-308.

103. Jones MP, Bushnell JA, Humphreys JS. Are rural placements positively associated with rural intentions in medical graduates? Med Educ. 2014;48(4):405-416. doi:10.1111/medu.12399

104. Kent M, Verstappen AC, Wilkinson T, Poole P. Keeping them interested: a national study of factors that change medical student interest in working rurally. Rural Remote Health. 2018;18(4):4872. doi:10.22605/rrh4872

105. Bath B, Gabrush J, Fritzler R, et al. Mapping the physiotherapy profession in Saskatchewan: examining rural versus urban practice patterns. Physiother Can. 2015;67(3):221-231. doi:10.3138/ptc.2014-53

106. Mathews M, Rourke JT, Park A. The contribution of Memorial University's medical school to rural physician supply. Can J Rural Med. 2008;13(1):15-21.

108. McGrail MR, Humphreys JS, Joyce CM. Nature of association between rural background and practice location: a comparison of general practitioners and specialists. BMC Health Serv Res. 2011;11:63. doi:10.1186/1472-6963-11-63

109. McGrail MR, Humphreys JS, Joyce CM, Scott A, Kalb G. How do rural GPs' workloads and work activities differ with community size compared with metropolitan practice? Aust J Prim Health. 2012;18(3):228-233. doi:10.1071/py11063

110. O'Connor M, Lee-Steere R. General practitioners' attitudes to palliative care: a Western Australian rural perspective. J Palliat Med. 2006;9(6):1271-1281. doi:10.1089/jpm.2006.9.1271

111. Orzanco MG, Lovato C, Bates J, Slade S, Grand'Maison P, Vanasse A. Nature and nurture in the family physician's choice of practice location. Rural Remote Health. 2011;11(3):1849.

112. Pathman DE, Konrad TR, King TS, Taylor DH Jr, Koch GG. Outcomes of states' scholarship, loan repayment, and related programs for physicians. Med Care. 2004;42(6):560-568. doi:10.1097/01.mlr.0000128003.81622.ef

113. Puddey IB, Mercer A, Playford DE, Riley GJ. Medical student selection criteria and socio-demographic factors as predictors of ultimately working rurally after graduation. BMC Med Educ. 2015;15:74. doi:10.1186/s12909-015-0359-5

114. Roots RK, Brown H, Bainbridge L, Li LC. Rural rehabilitation practice: perspectives of occupational therapists and physical therapists in British Columbia, Canada. Rural Remote Health. 2014;14:2506.

115. Rourke J, O'Keefe D, Ravalia M, et al. Pathways to rural family practice at Memorial University of Newfoundland. Can Fam Physician. 2018;64(3):e115-e125.

116. Burnett WH, Mark DH, Midtling JE, Zellner BB. Primary care physicians in underserved areas. Family physicians dominate. West J Med. 1995;163(6):532-536.

118. Szafran O, Crutcher RA, Chaytors RG. Location of family medicine graduates' practices. What factors influence Albertans' choices? Can Fam Physician. 2001;47:2279-2285.

119. Thomas CR, Holzer CE 3rd. The continuing shortage of child and adolescent psychiatrists. J Am Acad Child Adolesc Psychiatry. 2006;45(9):1023-1031. doi:10.1097/01.chi.0000225353.16831.5d

120. van Hassel D, Verheij R, Batenburg R. Assessing the variation in workload among general practitioners in urban and rural areas: an analysis based on SMS time sampling data. Int J Health Plann Manage. 2019;34(1):e474-e486. doi:10.1002/hpm.2663

121. Woloschuk W, Tarrant M. Does a rural educational experience influence students' likelihood of rural practice? Impact of student background and gender. Med Educ. 2002;36(3):241-247. doi:10.1046/j.1365-2923.2002.01143.x

122. Wood D. Effects of educational focus on a graduate nurse's initial choice of practice area. J Prof Nurs. 1998;14(4):214-219. doi:10.1016/s8755-7223(98)80061-6

123. Wood BR, Bell C, Carr J, et al. Washington state satellite HIV clinic program: a model for delivering highly effective decentralized care in under-resourced communities. AIDS Care. 2018;30(9):1120-1127. doi:10.1080/09540121.2018.1481194

124. Bushy A, Leipert BD. Factors that influence students in choosing rural nursing practice: a pilot study. Rural Remote Health. 2005;5(2):387.

125. Butler C, Sheppard L. The impact of undergraduate rural education on recently graduated physiotherapists. Aust J Physiother. 1999;45(1):23-31. doi:10.1016/s0004-9514(14)60339-1

126. Carter RG. The relation between personal characteristics of physicians and practice location in Manitoba. CMAJ. 1987;136(4):366-368.

127. Chan BT, Degani N, Crichton T, et al. Factors influencing family physicians to enter rural practice: does rural or urban background make a difference? Can Fam Physician. 2005;51(9):1246-1247.

128. Chen C, Petterson S, Phillips RL, Mullan F, Bazemore A, O'Donnell SD. Toward graduate medical education (GME) accountability: measuring the outcomes of GME institutions. Acad Med. 2013;88(9):1267-1280. doi:10.1097/ACM.0b013e31829a3ce9

129. Cramer M, Nienaber J, Helget P, Agrawal S. Comparative analysis of urban and rural nursing workforce shortages in Nebraska hospitals. Policy Polit Nurs Pract. 2006;7(4):248-260. doi:10.1177/1527154406296481

130. Bamford EJ, Dunne L, Taylor DS, Symon BG, Hugo GJ, Wilkinson D. Accessibility to general practitioners in rural South Australia. A case study using geographic information system technology. Med J Aust. 1999;171(11-12):614-616.

131. Doogan NJ, Roberts ME, Wewers ME, Tanenbaum ER, Mumford EA, Stillman FA. Validation of a new continuous geographic isolation scale: a tool for rural health disparities research. Soc Sci Med. 2018;215:123-132. doi:10.1016/j.socscimed.2018.09.005

132. Gilliland JA, Shah TI, Clark A, Sibbald S, Seabrook JA. A geospatial approach to understanding inequalities in accessibility to primary care among vulnerable populations. PLoS One. 2019;14(1):e0210113. doi:10.1371/journal.pone.0210113

133. Jo O, Kruger E, Tennant M. Geospatial analysis of the urban and rural/remote distribution of dental services in Scotland, Wales and Northern Ireland. Int Dent J. 2020;70(6):444-454. doi:10.1111/idj.12590

134. Lavergne MR. Identifying distinct geographic health service environments in British Columbia, Canada: cluster analysis of population-based administrative data. Healthc Policy. 2016;12(1):43-51.

135. Pearce J, Witten K, Bartie P. Neighbourhoods and health: a GIS approach to measuring community resource accessibility. J Epidemiol Community Health. 2006;60(5):389-395. doi:10.1136/jech.2005.043281

136. Playford DE, Mercer A, Carr SE, Puddey IB. Likelihood of rural practice in medical school entrants with prior tertiary experience. Med Teach. 2019;41(7):765-772. doi:10.1080/0142159x.2019.1570099

138. Ceronsky L, Shearer J, Weng K, Hopkins M, McKinley D. Minnesota Rural Palliative Care Initiative: building palliative care capacity in rural Minnesota. J Palliat Med. 2013;16(3):310-313. doi:10.1089/jpm.2012.0324

139. Griffin B, Porfeli E, Hu W. Who do you think you are? Medical student socioeconomic status and intention to work in underserved areas. Adv Health Sci Educ Theory Pract. 2017;22(2):491-504. doi:10.1007/s10459-016-9726-1

140. Abid Y, Connell CJW, Sijnja B, Verstappen AC, Poole P. National study of the impact of rural immersion programs on intended location of medical practice in New Zealand. Rural Remote Health. 2020;20(4):5785. doi:10.22605/rrh5785

141. Pathman DE, Konrad TR, Ricketts TC 3rd. The comparative retention of National Health Service Corps and other rural physicians. Results of a 9-year follow-up study. JAMA. 1992;268(12):1552-1558.

142. Pathman DE, Steiner BD, Jones BD, Konrad TR. Preparing and retaining rural physicians through medical education. Acad Med. 1999;74(7):810-820. doi:10.1097/00001888-199907000-00016

143. Pegram RW, Humphreys JS, Calcino G. Primary medical care workforce enumeration in rural and remote areas of Australia: time for a new approach? Aust J Rural Health. 2006;14(1):24-28. doi:10.1111/j.1440-1584.2006.00751.x

145. Smith JL. Examination of the relative importance of hospital employment in non-metropolitan counties using location quotients. Rural Remote Health. 2013;13(3):2497.

146. Zhu X, Mueller KJ, Vaughn T, Ullrich F. A rural taxonomy of population and health-resource characteristics. Rural Policy Brief. 2015;(2015 4):1-6.

147. Boscardin CK, Grbic D, Grumbach K, O'Sullivan P. Educational and individual factors associated with positive change in and reaffirmation of medical students' intention to practice in underserved areas. Acad Med. 2014;89(11):1490-1496. doi:10.1097/acm.0000000000000474

148. Duma O, Anton D, Tarţău L, Mocanu V. [Medical-social aspects of medical staff migration]. Rev Med Chir Soc Med Nat Iasi. 2011;115(2):507-511. [Romanian].

149. Garcia AN, Kuo T, Arangua L, Pérez-Stable EJ. Factors associated with medical school graduates' intention to work with underserved populations: policy implications for advancing workforce diversity. Acad Med. 2018;93(1):82-89. doi:10.1097/acm.0000000000001917

150. O'Connell TF, Ham SA, Hart TG, Curlin FA, Yoon JD. A national longitudinal survey of medical students' intentions to practice among the underserved. Acad Med. 2018;93(1):90-97. doi:10.1097/acm.0000000000001816

151. Bigbee J, Mixon D. Recruitment and retention of rural nursing students: a retrospective study. Rural Remote Health. 2013;13(4):2486.

152. Butler DC, Petterson S, Bazemore A, Douglas KA. Use of measures of socioeconomic deprivation in planning primary health care workforce and defining health care need in Australia. Aust J Rural Health. 2010;18(5):199-204. doi:10.1111/j.1440-1584.2010.01154.x

153. Horner RD, Samsa GP, Ricketts TC 3rd. Preliminary evidence on retention rates of primary care physicians in rural and urban areas. Med Care. 1993;31(7):640-648. doi:10.1097/00005650-199307000-00006

154. Humphreys JS. Delimiting 'rural': implications of an agreed 'rurality' index for healthcare planning and resource allocation. Aust J Rural Health. 1998;6(4):212-216. doi:10.1111/j.1440-1584.1998.tb00315.x

155. Kulig JC, Andrews ME, Stewart NJ, et al. How do registered nurses define rurality? Aust J Rural Health. 2008;16(1):28-32. doi:10.1111/j.1440-1584.2007.00947.x

156. McGirr J, Seal A, Barnard A, et al. The Australian Rural Clinical School (RCS) program supports rural medical workforce: evidence from a cross-sectional study of 12 RCSs. Rural Remote Health. 2019;19(1):4971. doi:10.22605/rrh4971

157. McGrail MR, Wingrove PM, Petterson SM, Humphreys JS, Russell DJ, Bazemore AW. Measuring the attractiveness of rural communities in accounting for differences of rural primary care workforce supply. Rural Remote Health. 2017;17(2):3925. doi:10.22605/rrh3925

158. McGrail MR, Russell DJ, O'Sullivan BG, Reeve C, Gasser L, Campbell D. Demonstrating a new approach to planning and monitoring rural medical training distribution to meet population need in North West Queensland. BMC Health Serv Res. 2018;18(1):993. doi:10.1186/s12913-018-3788-0

159. Steinhaeuser J, Otto P, Goetz K, Szecsenyi J, Joos S. Rural area in a European country from a health care point of view: an adoption of the Rural Ranking Scale. BMC Health Serv Res. 2014;14:147. doi:10.1186/1472-6963-14-147

160. Hills D, Joyce C, Humphreys J. Validation of a job satisfaction scale in the Australian clinical medical workforce. Eval Health Prof. 2012;35(1):47-76. doi:10.1177/0163278710397339

161. Rolfe IE, Pearson SA, O'Connell DL, Dickinson JA. Finding solutions to the rural doctor shortage: the roles of selection versus undergraduate medical education at Newcastle. Aust N Z J Med. 1995;25(5):512-517. doi:10.1111/j.1445-5994.1995.tb01497.x

162. Hays RB, Veitch PC, Cheers B, Crossland L. Why doctors leave rural practice. Aust J Rural Health. 1997;5(4):198-203. doi:10.1111/j.1440-1584.1997.tb00267.x

163. Humphreys JS, Jones MP, Jones JA, Mara PR. Workforce retention in rural and remote Australia: determining the factors that influence length of practice. Med J Aust. 2002;176(10):472-476. doi:10.5694/j.1326-5377.2002.tb04518.x

164. Laurence CO, Williamson V, Sumner KE, Fleming J. "Latte rural": the tangible and intangible factors important in the choice of a rural practice by recent GP graduates. Rural Remote Health. 2010;10(2):1316.

165. Hoffmann K, Wojczewski S, George A, Schäfer WL, Maier M. Stressed and overworked? A cross-sectional study of the working situation of urban and rural general practitioners in Austria in the framework of the QUALICOPC project. Croat Med J. 2015;56(4):366-374. doi:10.3325/cmj.2015.56.366

166. Janes R, Elley R, Dowell A. New Zealand Rural General Practitioners 1999 Survey--Part 2: gender issues. N Z Med J. 2004;117(1191):U814.

167. Chisholm M, Russell D, Humphreys J. Measuring rural allied health workforce turnover and retention: what are the patterns, determinants and costs? Aust J Rural Health. 2011;19(2):81-88. doi:10.1111/j.1440-1584.2011.01188.x

168. Lee S, Mackenzie L. Starting out in rural New South Wales: the experiences of new graduate occupational therapists. Aust J Rural Health. 2003;11(1):36-43. doi:10.1046/j.1440-1584.2003.00476.x

169. Wainer J. Work of female rural doctors. Aust J Rural Health. 2004;12(2):49-53. doi:10.1111/j.1038-5282.2004.00557.x

170. Smith T, Cross M, Waller S, et al. Ruralization of students' horizons: insights into Australian health professional students' rural and remote placements. J Multidiscip Healthc. 2018;11:85-97. doi:10.2147/jmdh.s150623

171. Humphreys JS, Jones JA, Jones MP, et al. The influence of geographical location on the complexity of rural general practice activities. Med J Aust. 2003;179(8):416-420. doi:10.5694/j.1326-5377.2003.tb05619.x

172. Cameron PJ, Este DC, Worthington CA. Physician retention in rural Alberta: key community factors. Can J Public Health. 2010;101(1):79-82. doi:10.1007/bf03405568

173. Rabinowitz HK, Diamond JJ, Hojat M, Hazelwood CE. Demographic, educational and economic factors related to recruitment and retention of physicians in rural Pennsylvania. J Rural Health. 1999;15(2):212-218. doi:10.1111/j.1748-0361.1999.tb00742.x

174. Keane S, Smith T, Lincoln M, Fisher K. Survey of the rural allied health workforce in New South Wales to inform recruitment and retention. Aust J Rural Health. 2011;19(1):38-44. doi:10.1111/j.1440-1584.2010.01175.x

175. Eley DS, Synnott R, Baker PG, Chater AB. A decade of Australian Rural Clinical School graduates--where are they and why? Rural Remote Health. 2012;12:1937.

176. Stagg P, Greenhill J, Worley PS. A new model to understand the career choice and practice location decisions of medical graduates. Rural Remote Health. 2009;9(4):1245.

177. King KR, Purcell RA, Quinn SJ, Schoo AM, Walters LK. Supports for medical students during rural clinical placements: factors associated with intention to practise in rural locations. Rural Remote Health. 2016;16(2):3791.

180. McGrail MR, Humphreys JS. Spatial access disparities to primary health care in rural and remote Australia. Geospat Health. 2015;10(2):358. doi:10.4081/gh.2015.358

181. Puddey IB, Mercer A, Playford DE, Pougnault S, Riley GJ. Medical student selection criteria as predictors of intended rural practice following graduation. BMC Med Educ. 2014;14:218. doi:10.1186/1472-6920-14-218

182. Australian Medical Workforce Advisory Committee (AMWAC). Doctors in vocational training: rural background and rural practice intentions. Aust J Rural Health. 2005;13(1):14-20. doi:10.1111/j.1440-1854.2004.00640.x

183. Beauchamp J, Bélanger M, Schofield A, Bordage R, Donovan D, Landry M. Recruiting doctors from and for underserved groups: does New Brunswick's initiative to recruit doctors for its linguistic minority help rural communities? Can J Public Health. 2013;104(6 Suppl 1):S44-48. doi:10.17269/cjph.104.3478

184. Kondalsamy-Chennakesavan S, Eley DS, Ranmuthugala G, et al. Determinants of rural practice: positive interaction between rural background and rural undergraduate training. Med J Aust. 2015;202(1):41-45. doi:10.5694/mja14.00236

185. Herd MS, Bulsara MK, Jones MP, Mak DB. Preferred practice location at medical school commencement strongly determines graduates' rural preferences and work locations. Aust J Rural Health. 2017;25(1):15-21. doi:10.1111/ajr.12301

187. Kwan MMS, Kondalsamy-Chennakesavan S, Ranmuthugala G, Toombs MR, Nicholson GC. The rural pipeline to longer-term rural practice: general practitioners and specialists. PLoS One. 2017;12(7):e0180394. doi:10.1371/journal.pone.0180394

188. Playford D, Ngo H, Gupta S, Puddey IB. Opting for rural practice: the influence of medical student origin, intention and immersion experience. Med J Aust. 2017;207(4):154-158. doi:10.5694/mja16.01322

189. Rabinowitz HK, Diamond JJ, Markham FW, Santana AJ. The relationship between entering medical students' backgrounds and career plans and their rural practice outcomes three decades later. Acad Med. 2012;87(4):493-497. doi:10.1097/ACM.0b013e3182488c06

190. Rabinowitz HK, Diamond JJ, Markham FW, Paynter NP. Critical factors for designing programs to increase the supply and retention of rural primary care physicians. JAMA. 2001;286(9):1041-1048. doi:10.1001/jama.286.9.1041

191. Rabinowitz HK, Diamond JJ, Veloski JJ, Gayle JA. The impact of multiple predictors on generalist physicians' care of underserved populations. Am J Public Health. 2000;90(8):1225-1228. doi:10.2105/ajph.90.8.1225

192. Runge CE, MacKenzie A, Loos C, et al. Characteristics of Queensland physicians and the influence of rural exposure on practice location. Intern Med J. 2016;46(8):981-985. doi:10.1111/imj.13156

193. Sen Gupta T, Woolley T, Murray R, Hays R, McCloskey T. Positive impacts on rural and regional workforce from the first seven cohorts of James Cook University medical graduates. Rural Remote Health. 2014;14:2657.

194. Smith T, Sutton K, Beauchamp A, et al. Profile and rural exposure for nursing and allied health students at two Australian universities: a retrospective cohort study. Aust J Rural Health. 2021;29(1):21-33. doi:10.1111/ajr.12689

195. Strasser R, Hogenbirk JC, Lewenberg M, Story M, Kevat A. Starting rural, staying rural: how can we strengthen the pathway from rural upbringing to rural practice? Aust J Rural Health. 2010;18(6):242-248. doi:10.1111/j.1440-1584.2010.01167.x

196. Woolley T, Sen Gupta T, Murray R, Hays R. Predictors of rural practice location for James Cook University MBBS graduates at postgraduate year 5. Aust J Rural Health. 2014;22(4):165-171. doi:10.1111/ajr.12106

197. Drovandi A, Woolley T. Workforce supply of pharmacists in Queensland communities from James Cook University Pharmacy Graduates. Aust J Rural Health. 2020;28(5):462-468. doi:10.1111/ajr.12662

199. Playford DE, Ng WQ, Burkitt T. Creation of a mobile rural workforce following undergraduate longitudinal rural immersion. Med Teach. 2016;38(5):498-503. doi:10.3109/0142159x.2015.1060304

200. Rosenblatt RA, Whitcomb ME, Cullen TJ, Lishner DM, Hart LG. Which medical schools produce rural physicians? JAMA. 1992;268(12):1559-1565.

201. Sen Gupta T, Murray R, Hays R, Woolley T. James Cook University MBBS graduate intentions and intern destinations: a comparative study with other Queensland and Australian medical schools. Rural Remote Health. 2013;13(2):2313.

202. Somers GT, Spencer RJ. Nature or nurture: the effect of undergraduate rural clinical rotations on pre-existent rural career choice likelihood as measured by the SOMERS Index. Aust J Rural Health. 2012;20(2):80-87. doi:10.1111/j.1440-1584.2012.01258.x

203. Bailey BE, Wharton RG, Holman CD. Glass half full: survival analysis of new rural doctor retention in Western Australia. Aust J Rural Health. 2016;24(4):258-264. doi:10.1111/ajr.12260

204. Shires L, Allen P, Cheek C, Deb W. Regional universities and rural clinical schools contribute to rural medical workforce, a cohort study of 2002 to 2013 graduates. Rural Remote Health. 2015;15(3):3219.

205. Gupta S, Ngo H, Burkitt T, Puddey I, Playford D. Survival analysis of Rural Clinical School of Western Australia graduates: the long-term work of building a long-term rural medical workforce. BMC Health Serv Res. 2019;19(1):998. doi:10.1186/s12913-019-4816-4

206. Clark TR, Freedman SB, Croft AJ, et al. Medical graduates becoming rural doctors: rural background versus extended rural placement. Med J Aust. 2013;199(11):779-782. doi:10.5694/mja13.10036

207. Zink T, Center B, Finstad D, et al. Efforts to graduate more primary care physicians and physicians who will practice in rural areas: examining outcomes from the University of Minnesota-Duluth and the rural physician associate program. Acad Med. 2010;85(4):599-604. doi:10.1097/ACM.0b013e3181d2b537

208. Young L, Kent L, Walters L. The John Flynn Placement Program: evidence for repeated rural exposure for medical students. Aust J Rural Health. 2011;19(3):147-153. doi:10.1111/j.1440-1584.2011.01201.x

209. Eley D, Baker P, Chater B. The Rural Clinical School Tracking Project: more IS better--confirming factors that influence early career entry into the rural medical workforce. Med Teach. 2009;31(10):e454-459. doi:10.3109/01421590902850857

210. May J, Brown L, Burrows J. In-place training: optimizing rural health workforce outcomes through rural-based education in Australia. Educ Sci. 2018;8(1):20. doi:10.3390/educsci8010020

211. Playford DE, Evans SF, Atkinson DN, Auret KA, Riley GJ. Impact of the Rural Clinical School of Western Australia on work location of medical graduates. Med J Aust. 2014;200(2):104-107. doi:10.5694/mja13.11082

212. Wendling AL, Phillips J, Short W, Fahey C, Mavis B. Thirty years training rural physicians: outcomes from the Michigan State University College of Human Medicine rural physician program. Acad Med. 2016;91(1):113-119. doi:10.1097/acm.0000000000000885

213. Kane KY, Quinn KJ, Stevermer JJ, et al. Summer in the country: changes in medical students' perceptions following an innovative rural community experience. Acad Med. 2013;88(8):1157-1163. doi:10.1097/ACM.0b013e318299fb5d

214. Wilkinson D, Laven G, Pratt N, Beilby J. Impact of undergraduate and postgraduate rural training, and medical school entry criteria on rural practice among Australian general practitioners: national study of 2414 doctors. Med Educ. 2003;37(9):809-814. doi:10.1046/j.1365-2923.2003.01596.x

215. Brockwell D, Wielandt T, Clark M. Four years after graduation: occupational therapists' work destinations and perceptions of preparedness for practice. Aust J Rural Health. 2009;17(2):71-76. doi:10.1111/j.1440-1584.2008.01020.x

216. Emery A, Hurley S, Williams J, Pougnault S, Mercer A, Tennant M. A seven-year retrospective analysis of students entering medicine via a rural student recruitment program in Western Australia. Aust J Rural Health. 2009;17(6):316-320. doi:10.1111/j.1440-1584.2009.01105.x

217. Jamar E, Newbury J, Mills D. Early career location of University of Adelaide rural cohort medical students. Rural Remote Health. 2014;14:2592.

218. Rabinowitz HK. Evaluation of a selective medical school admissions policy to increase the number of family physicians in rural and underserved areas. N Engl J Med. 1988;319(8):480-486. doi:10.1056/nejm198808253190805

219. Rabinowitz HK. Recruitment, retention, and follow-up of graduates of a program to increase the number of family physicians in rural and underserved areas. N Engl J Med. 1993;328(13):934-939. doi:10.1056/nejm199304013281307

220. Rabinowitz HK, Diamond JJ, Markham FW, Santana AJ. Retention of rural family physicians after 20-25 years: outcomes of a comprehensive medical school rural program. J Am Board Fam Med. 2013;26(1):24-27. doi:10.3122/jabfm.2013.01.120122

222. Longenecker RL, Andrilla CHA, Jopson AD, et al. Pipelines to pathways: medical school commitment to producing a rural workforce. J Rural Health. 2021;37(4):723-733. doi:10.1111/jrh.12542

223. McGrail MR, Russell DJ, Campbell DG. Vocational training of general practitioners in rural locations is critical for the Australian rural medical workforce. Med J Aust. 2016;205(5):216-221. doi:10.5694/mja16.00063

224. Hajat A, Stewart K, Hayes KL. The local public health workforce in rural communities. J Public Health Manag Pract. 2003;9(6):481-488. doi:10.1097/00124784-200311000-00007

225. Jones JA, Humphreys JS, Adena MA. Rural GPs' ratings of initiatives designed to improve rural medical workforce recruitment and retention. Rural Remote Health. 2004;4(3):314.

226. Russell DJ, Humphreys JS, McGrail MR, Cameron WI, Williams PJ. The value of survival analyses for evidence-based rural medical workforce planning. Hum Resour Health. 2013;11:65. doi:10.1186/1478-4491-11-65

227. Ray RA, Fried O, Lindsay D. Palliative care professional education via video conference builds confidence to deliver palliative care in rural and remote locations. BMC Health Serv Res. 2014;14:272. doi:10.1186/1472-6963-14-272

228. Humphreys JS, McGrail MR, Joyce CM, Scott A, Kalb G. Who should receive recruitment and retention incentives? Improved targeting of rural doctors using medical workforce data. Aust J Rural Health. 2012;20(1):3-10. doi:10.1111/j.1440-1584.2011.01252.x

229. Kuhn B, Kleij KS, Liersch S, Steinhäuser J, Amelung V. Which strategies might improve local primary healthcare in Germany? An explorative study from a local government point of view. BMC Fam Pract. 2017;18(1):105. doi:10.1186/s12875-017-0696-z

230. Irby MB, Boles KA, Jordan C, Skelton JA. TeleFIT: adapting a multidisciplinary, tertiary-care pediatric obesity clinic to rural populations. Telemed J E Health. 2012;18(3):247-249. doi:10.1089/tmj.2011.0117

234. Royston PJ, Mathieson K, Leafman J, Ojan-Sheehan O. Medical student characteristics predictive of intent for rural practice. Rural Remote Health. 2012;12:2107.

238. Huicho L, Dieleman M, Campbell J, et al. Increasing access to health workers in underserved areas: a conceptual framework for measuring results. Bull World Health Organ. 2010;88(5):357-363. doi:10.2471/blt.09.070920

240. Courtney KL. Visualizing nursing workforce distribution: policy evaluation using geographic information systems. Int J Med Inform. 2005;74(11-12):980-988. doi:10.1016/j.ijmedinf.2005.07.012

241. Feng H, Berk-Krauss J, Feng PW, Stein JA. Comparison of dermatologist density between urban and rural counties in the United States. JAMA Dermatol. 2018;154(11):1265-1271. doi:10.1001/jamadermatol.2018.3022

242. Fryer GE Jr, Call RL, Heine C, Casamassimo P. The validity of indices for rural health manpower needs assessment. Eval Program Plann. 1983;6(2):139-142. doi:10.1016/0149-7189(83)90027-7

243. Gemelas JC. Post-ACA trends in the US primary care physician shortage with index of relative rurality. J Rural Health. 2021;37(4):700-704. doi:10.1111/jrh.12506

244. Godwin D, Blizzard L, Hoang H, Crocombe L. Evidence of the effect of rural background on rural practise in Australian dental practitioners: does gender play a role? Aust Dent J. 2017;62(1):30-38. doi:10.1111/adj.12442

245. Goetz K, Musselmann B, Szecsenyi J, Joos S. The influence of workload and health behavior on job satisfaction of general practitioners. Fam Med. 2013;45(2):95-101.

246. Hansroth J, Findley SW, Quedado KD, Marshall T, Vucelik A, Goode CS. Evaluating West Virginia's emergency medicine workforce: a longitudinal observational study. Cureus. 2021;13(3):e13639. doi:10.7759/cureus.13639

247. Iversen L, Farmer JC, Hannaford PC. Workload pressures in rural general practice: a qualitative investigation. Scand J Prim Health Care. 2002;20(3):139-144. doi:10.1080/028134302760234573

248. 248 Johnston A, Haber J, Malhi R, Nichols D, Williamson R. Defining rural teaching hospitals in Canada: Developing and testing a new definition. Can J Rural Med. 2020;25(4):145-149. doi:10.4103/cjrm.cjrm_21_20

249. Joyce C, Wolfe R. Geographic distribution of the Australian primary health workforce in 1996 and 2001. Aust N Z J Public Health. 2005;29(2):129-135. doi:10.1111/j.1467-842x.2005.tb00062.x

250. Kippenbrock T, Buron B, Odell E, Narcisse MR. Minimal changes and missed opportunities: a decade look at nurse practitioners in the lower Mississippi River Delta states. J Prof Nurs. 2014;30(3):266-272. doi:10.1016/j.profnurs.2013.09.014

251. Kuipers P, Hurwood A, McBride LJ. Audit of allied health assistant roles: suggestions for improving quality in rural settings. Aust J Rural Health. 2015;23(3):185-188. doi:10.1111/ajr.12161

252. Larson EH, Palazzo L, Berkowitz B, Pirani MJ, Hart LG. The contribution of nurse practitioners and physician assistants to generalist care in Washington State. Health Serv Res. 2003;38(4):1033-1050. doi:10.1111/1475-6773.00161

253. Laskowska I. Availability of health services vs. health condition of residents of rural areas in Poland - analysis performed on the basis of EHIS 2009. Ann Agric Environ Med. 2015;22(4):700-703. doi:10.5604/12321966.1185779

254. Mathews M, Seguin M, Chowdhury N, Card RT. Generational differences in factors influencing physicians to choose a work location. Rural Remote Health. 2012;12:1864.

255. Mathews M, Ryan D, Samarasena A. Work locations in 2014 of medical graduates of Memorial University of Newfoundland: a cross-sectional study. CMAJ Open. 2015;3(2):E217-222. doi:10.9778/cmajo.20140109

256. McGrail MR, Humphreys JS. A new index of access to primary care services in rural areas. Aust N Z J Public Health. 2009;33(5):418-423. doi:10.1111/j.1753-6405.2009.00422.x

257. Montour A, Baumann A, Blythe J, Hunsberger M. The changing nature of nursing work in rural and small community hospitals. Rural Remote Health. 2009;9(1):1089.

258. Nugent P, Ogle KR, Bethune E, Walker A, Wellman DA. Undergraduate pre-registration nursing education in Australia: a longitudinal examination of enrollment and completion numbers with a focus on students from rural and remote campus locations. Rural Remote Health. 2004;4(4):313.

259. O'Sullivan B, McGrail M, Russell D, et al. Duration and setting of rural immersion during the medical degree relates to rural work outcomes. Med Educ. 2018;52(8):803-815. doi:10.1111/medu.13578

260. Pearce J, Witten K, Hiscock R, Blakely T. Regional and urban-rural variations in the association of neighbourhood deprivation with community resource access: a national study. Environ Plan A. 2008;40(10):2469-2489. doi:10.1068/a409

261. Russell DJ, Wakerman J, Humphreys JS. What is a reasonable length of employment for health workers in Australian rural and remote primary healthcare services? Aust Health Rev. 2013;37(2):256-261. doi:10.1071/ah12184

262. Senn N, Cohidon C, Zuchuat JC. Defining a typology of primary care practices: a novel approach. Int J Qual Health Care. 2016;28(6):734-741. doi:10.1093/intqhc/mzw102

263. Shah TI, Milosavljevic S, Bath B. Determining geographic accessibility of family physician and nurse practitioner services in relation to the distribution of seniors within two Canadian Prairie Provinces. Soc Sci Med. 2017;194:96-104. doi:10.1016/j.socscimed.2017.10.019

264. Strasser RP, Hays RB, Kamien M, Carson D. Is Australian rural practice changing? Findings from the National Rural General Practice Study. Aust J Rural Health. 2000;8(4):222-226. doi:10.1046/j.1440-1584.2000.00305.x

265. Wade ME, Brokaw JJ, Zollinger TW, et al. Influence of hometown on family physicians' choice to practice in rural settings. Fam Med. 2007;39(4):248-254.

266. Wan N, Zou B, Sternberg T. A three-step floating catchment area method for analyzing spatial access to health services. Int J Geogr Inf Sci. 2012;26(6):1073-1089. doi:10.1080/13658816.2011.624987

267. Willie-Stephens J, Kruger E, Tennant M. Public and private dental services in NSW: a geographic information system analysis of access to care for 7 million Australians. N S W Public Health Bull. 2014;24(4):164-170. doi:10.1071/nb13004

268. Yan W, Cheng TC, Scott A, et al. Medicine in Australia: Balancing Employment and Life (MABEL). Aust Econ Rev. 2011;44(1):102-112. doi:10.1111/j.1467-8462.2010.00627.x
