# Supplementary material for: Characteristics of Medical Deserts and Approaches to Mitigate Their Health Workforce Issues: A Scoping Review of Empirical Studies in Western Countries
Source: Int J Health Policy Manag. 2023 Aug 15;12:7454. doi: 10.34172/ijhpm.2023.7454 (PMC10590222; doi:10.34172/ijhpm.2023.7454)
Supplement: Supplementary file 5 — Definitions of Medical Deserts. [file ijhpm-12-7454-s005.pdf]

Article title: Characteristics of Medical Deserts and Approaches to Mitigate Their Health Workforce Issues: A Scoping Review of Empirical Studies in Western Countries

Journal name: International Journal of Health Policy and Management (IJHPM)

Authors' information: Linda E. Flinterman[1]*¶, Ana I. González-González[2]¶, Laura Seils[2], Julia Bes[1], Marta Ballester[2], Joaquim Bañeres[2], Sorin Dan[3], Alicja Domagala[4], Katarzyna Dubas-Jakóbczyk[5], Robert Likic[6], Marieke Kroezen[7], Ronald Batenburg[1,8]

[1]Health Workforce and Organization Studies, Netherlands Institute for Health Services Research (NIVEL), Utrecht, The Netherlands.

[2]Avedis Donabedian Research Institute – UAB, Madrid, Spain.

[3]Innovation and Entrepreneurship InnoLab, University of Vaasa, Vaasa, Finland.

[4]Department of Health Policy and Management, Institute of Public Health, Jagiellonian University, Krakow, Poland.

[5]Department of Health Economics and Social Security, Institute of Public Health, Jagiellonian University, Krakow, Poland.

[6]School of Medicine, University of Zagreb, Zagreb, Croatia.

[7]Trimbos Institute, Netherlands Institute of Mental Health and Addiction, Utrecht, The Netherlands.

[8]Department of Sociology, Radboud University, Nijmegen, The Netherlands.

¶ Both authors contributed equally to this paper.

(*Corresponding author: Email: l.flinterman@nivel.nl)

Citation: Flinterman LE, González-González AI, Seils L, et al. Characteristics of medical deserts and approaches to mitigate their health workforce issues: a scoping review of empirical studies in Western countries. Int J Health Policy Manag. 2023;12:7454. doi:10.34172/ijhpm.2023.7454

Supplementary file 5. Definitions of Medical Deserts

Definition	Main characteristics	Used in reference
Rural Remote and Metropolitan Area classification (RRMA) (332)	• Population per statistical local area<br>• Distance to facilities	(166,167,172,174,177,184,202,214,216,217,222,232,246,250,252,308,318,333,334)
General Practice Rurality Index (GPRI) (335)	• Remoteness from an advanced referral centre<br>• Remoteness from a basic referral centre<br>• Population size<br>• Number of general practitioners<br>• Number of specialists<br>• Presence of an acute care hospital	(203)
Accessibility Remoteness Index of Australia (ARIA) (336)	• Road distance to service centres	(223,277)
Australian Standard Geographical Classification- Remoteness area (ASGC-RA) (337)	• Road distance to service centres	(183,185,191–195,207,209,219,230,263,271,272,296,300,306,307,309,312,315,329)
Health Professional Shortage Areas (HPSA) (338)	• Population to provider ratio<br>• Percent of population below 100% of the federal poverty level<br>• Travel time to the nearest source of care outside the designation area	(253,283,339)
Modified Monash Model (MMM) (340)	• Based on ASGC/S-RA	(175,218,341)
Rural Urban Community Codes/Areas (RUCC/A) (342)	• Population size<br>• Adjacent to a metropolitan area yes/no	(208,220,256,264,286)
Index of Relative Rurality (IRR) (343)	• Population size<br>• Population density<br>• Extent of urbanized area<br>• Distance to nearest metropolitan area	(259)
Socio Economic Indexes For Areas (SEIFA) (344)	• Level of advantage/disadvantage<br>• Economic resources<br>• Education and occupation	(327)
Medically Underserved Area/Population MUA/P (338)	• Provider per 1000 population ratio<br>• Percent of population at 100% of the federal poverty level<br>• Percent of population age 65 and over<br>• Infant mortality rate	(314)

Definition	Main characteristics	Used in reference
2 Step Floating Catchment Area (2SFCA) (345)	• Number of services at location<br>• Distance to services<br>• Number of residents at location<br>• Health needs of the population<br>• Population provider ratio<br>• Mobility of the population	(187,346)
3 Step Floating Catchment Area (3SFCA)	• 2SFCA<br>• Availability of other nearby medical sites	(311,324)
Rural Ranking Scale (RRS)	• Travelling time from surgery to major hospital<br>• On call duty<br>• On-call for major traumas<br>• Travelling time to nearest GP colleague<br>• Travelling time to most distant practice boundary<br>• Travelling time to regular peripheral clinic	(160,170)
Typology of seven factors	• Geographic proximity to an urban centre<br>• Access to health care resources<br>• Size of community<br>• Geographic location<br>• Practice type<br>• Number of years for which at least one physician was retained<br>• Resources available to the community	(178)
Standard Metropolitan Statistical Area (SMSA)	• Population density of a county<br>• Part of the county that has a shortage of manpower<br>• % of the county population that is rural<br>• Population of the county<br>• Number of active physicians	(225)
Rural-Urban Density Typology (RUDT) (347)	• Population size<br>• Adjacent to a large or small metropolitan area<br>• Town in region	(196)
Rural and Remote Area (RARA) (348)	• Population size<br>• Distance to metropolitan area	(163)
Urban influence codes (349)	• Population size<br>• Size of largest city<br>• Proximity to metro- and micropolitan areas	(323)

Definition	Main characteristics	Used in reference
Urban centric location (350)	• Population size<br>• Inside or outside a (principal) city<br>• Distance to urbanized area	(221)

160 Hills D, Joyce C, Humphreys J. Validation of a job satisfaction scale in the Australian clinical medical workforce. *Eval Health Prof*. 2012;35(1):47-76. doi:10.1177/0163278710397339

163 Humphreys JS, Jones MP, Jones JA, Mara PR. Workforce retention in rural and remote Australia: determining the factors that influence length of practice. *Med J Aust*. 2002;176(10):472-476. doi:10.5694/j.1326-5377.2002.tb04518.x

166 Janes R, Elley R, Dowell A. New Zealand Rural General Practitioners 1999 Survey--Part 2: gender issues. *N Z Med J*. 2004;117(1191):U814.

167 Chisholm M, Russell D, Humphreys J. Measuring rural allied health workforce turnover and retention: what are the patterns, determinants and costs? *Aust J Rural Health*. 2011;19(2):81-88. doi:10.1111/j.1440-1584.2011.01188.x

170 Smith T, Cross M, Waller S, et al. Ruralization of students' horizons: insights into Australian health professional students' rural and remote placements. *J Multidiscip Healthc*. 2018;11:85-97. doi:10.2147/jmdh.s150623

172 Cameron PJ, Este DC, Worthington CA. Physician retention in rural Alberta: key community factors. *Can J Public Health*. 2010;101(1):79-82. doi:10.1007/bf03405568

174 Keane S, Smith T, Lincoln M, Fisher K. Survey of the rural allied health workforce in New South Wales to inform recruitment and retention. *Aust J Rural Health*. 2011;19(1):38-44. doi:10.1111/j.1440-1584.2010.01175.x
175 Eley DS, Synnott R, Baker PG, Chater AB. A decade of Australian Rural Clinical School graduates--where are they and why? *Rural Remote Health*. 2012;12:1937.

177 King KR, Purcell RA, Quinn SJ, Schoo AM, Walters LK. Supports for medical students during rural clinical placements: factors associated with intention to practise in rural locations. *Rural Remote Health*. 2016;16(2):3791.

178 Brockwell D, Wielandt T, Clark M. Four years after graduation: occupational therapists' work destinations and perceptions of preparedness for practice. *Aust J Rural Health*. 2009;17(2):71-76. doi:10.1111/j.1440-1584.2008.01020.x

183 Beauchamp J, Bélanger M, Schofield A, Bordage R, Donovan D, Landry M. Recruiting doctors from and for underserved groups: does New Brunswick's initiative to recruit doctors for its linguistic minority help rural communities? *Can J Public Health*. 2013;104(6 Suppl 1):S44-48. doi:10.17269/cjph.104.3478

184 Kondalsamy-Chennakesavan S, Eley DS, Ranmuthugala G, et al. Determinants of rural practice: positive interaction between rural background and rural undergraduate training. *Med J Aust*. 2015;202(1):41-45. doi:10.5694/mja14.00236

185 Herd MS, Bulsara MK, Jones MP, Mak DB. Preferred practice location at medical school commencement strongly determines graduates' rural preferences and work locations. *Aust J Rural Health*. 2017;25(1):15-21. doi:10.1111/ajr.12301

187 Kwan MMS, Kondalsamy-Chennakesavan S, Ranmuthugala G, Toombs MR, Nicholson GC. The rural pipeline to longer-term rural practice: general practitioners and specialists. *PLoS One*. 2017;12(7):e0180394. doi:10.1371/journal.pone.0180394

191 Rabinowitz HK, Diamond JJ, Veloski JJ, Gayle JA. The impact of multiple predictors on generalist physicians' care of underserved populations. *Am J Public Health*. 2000;90(8):1225-1228. doi:10.2105/ajph.90.8.1225

192 Runge CE, MacKenzie A, Loos C, et al. Characteristics of Queensland physicians and the influence of rural exposure on practice location. *Intern Med J*. 2016;46(8):981-985. doi:10.1111/imj.13156

193 Sen Gupta T, Woolley T, Murray R, Hays R, McCloskey T. Positive impacts on rural and regional workforce from the first seven cohorts of James Cook University medical graduates. *Rural Remote Health*. 2014;14:2657.

194 Smith T, Sutton K, Beauchamp A, et al. Profile and rural exposure for nursing and allied health students at two Australian universities: a retrospective cohort study. *Aust J Rural Health*. 2021;29(1):21-33. doi:10.1111/ajr.12689

195 Strasser R, Hogenbirk JC, Lewenberg M, Story M, Kevat A. Starting rural, staying rural: how can we strengthen the pathway from rural upbringing to rural practice? *Aust J Rural Health*. 2010;18(6):242-248. doi:10.1111/j.1440-1584.2010.01167.x

196 Woolley T, Sen Gupta T, Murray R, Hays R. Predictors of rural practice location for James Cook University MBBS graduates at postgraduate year 5. *Aust J Rural Health*. 2014;22(4):165-171. doi:10.1111/ajr.12106

202 Somers GT, Spencer RJ. Nature or nurture: the effect of undergraduate rural clinical rotations on pre-existent rural career choice likelihood as measured by the SOMERS Index. *Aust J Rural Health*. 2012;20(2):80-87. doi:10.1111/j.1440-1584.2012.01258.x

203 Bailey BE, Wharton RG, Holman CD. Glass half full: survival analysis of new rural doctor retention in Western Australia. *Aust J Rural Health*. 2016;24(4):258-264. doi:10.1111/ajr.12260

207 Zink T, Center B, Finstad D, et al. Efforts to graduate more primary care physicians and physicians who will practice in rural areas: examining outcomes from the University of Minnesota-Duluth and the rural physician associate program. *Acad Med*. 2010;85(4):599-604. doi:10.1097/ACM.0b013e3181d2b537

208 Young L, Kent L, Walters L. The John Flynn Placement Program: evidence for repeated rural exposure for medical students. *Aust J Rural Health*. 2011;19(3):147-153. doi:10.1111/j.1440-1584.2011.01201.x

209 Eley D, Baker P, Chater B. The Rural Clinical School Tracking Project: more IS better--confirming factors that influence early career entry into the rural medical workforce. *Med Teach*. 2009;31(10):e454-459. doi:10.3109/01421590902850857

214 Wilkinson D, Laven G, Pratt N, Beilby J. Impact of undergraduate and postgraduate rural training, and medical school entry criteria on rural practice among Australian general practitioners: national study of 2414 doctors. *Med Educ*. 2003;37(9):809-814. doi:10.1046/j.1365-2923.2003.01596.x

216 Emery A, Hurley S, Williams J, Pougnault S, Mercer A, Tennant M. A seven-year retrospective analysis of students entering medicine via a rural student recruitment program in Western Australia. *Aust J Rural Health*. 2009;17(6):316-320. doi:10.1111/j.1440-1584.2009.01105.x

217 Jamar E, Newbury J, Mills D. Early career location of University of Adelaide rural cohort medical students. *Rural Remote Health*. 2014;14:2592.

218 Rabinowitz HK. Evaluation of a selective medical school admissions policy to increase the number of family physicians in rural and underserved areas. *N Engl J Med*. 1988;319(8):480-486. doi:10.1056/nejm198808253190805

219 Rabinowitz HK. Recruitment, retention, and follow-up of graduates of a program to increase the number of family physicians in rural and underserved areas. *N Engl J Med*. 1993;328(13):934-939. doi:10.1056/nejm199304013281307

220 Rabinowitz HK, Diamond JJ, Markham FW, Santana AJ. Retention of rural family physicians after 20-25 years: outcomes of a comprehensive medical school rural program. *J Am Board Fam Med*. 2013;26(1):24-27. doi:10.3122/jabfm.2013.01.120122

221 Rabinowitz HK, Diamond JJ, Markham FW, Hazelwood CE. A program to increase the number of family physicians in rural and underserved areas: impact after 22 years. *JAMA*. 1999;281(3):255-260. doi:10.1001/jama.281.3.255

222 Longenecker RL, Andrilla CHA, Jopson AD, et al. Pipelines to pathways: medical school commitment to producing a rural workforce. *J Rural Health*. 2021;37(4):723-733. doi:10.1111/jrh.12542

223 McGrail MR, Russell DJ, Campbell DG. Vocational training of general practitioners in rural locations is critical for the Australian rural medical workforce. *Med J Aust*. 2016;205(5):216-221. doi:10.5694/mja16.00063

225 Jones JA, Humphreys JS, Adena MA. Rural GPs' ratings of initiatives designed to improve rural medical workforce recruitment and retention. *Rural Remote Health*. 2004;4(3):314.

230 Irby MB, Boles KA, Jordan C, Skelton JA. TeleFIT: adapting a multidisciplinary, tertiary-care pediatric obesity clinic to rural populations. *Telemed J E Health*. 2012;18(3):247-249. doi:10.1089/tmj.2011.0117

232 Dolea C, Stormont L, Braichet JM. Evaluated strategies to increase attraction and retention of health workers in remote and rural areas. *Bull World Health Organ*. 2010;88(5):379-385. doi:10.2471/blt.09.070607
246 Hansroth J, Findley SW, Quedado KD, Marshall T, Vucelik A, Goode CS. Evaluating West Virginia's emergency medicine workforce: a longitudinal observational study. *Cureus*. 2021;13(3):e13639. doi:10.7759/cureus.13639

250 Kippenbrock T, Buron B, Odell E, Narcisse MR. Minimal changes and missed opportunities: a decade look at nurse practitioners in the lower Mississippi River Delta states. *J Prof Nurs*. 2014;30(3):266-272. doi:10.1016/j.profnurs.2013.09.014

252 Larson EH, Palazzo L, Berkowitz B, Pirani MJ, Hart LG. The contribution of nurse practitioners and physician assistants to generalist care in Washington State. *Health Serv Res*. 2003;38(4):1033-1050. doi:10.1111/1475-6773.00161

253 Laskowska I. Availability of health services vs. health condition of residents of rural areas in Poland - analysis performed on the basis of EHIS 2009. *Ann Agric Environ Med*. 2015;22(4):700-703. doi:10.5604/12321966.1185779

256 McGrail MR, Humphreys JS. A new index of access to primary care services in rural areas. *Aust N Z J Public Health*. 2009;33(5):418-423. doi:10.1111/j.1753-6405.2009.00422.x

259 O'Sullivan B, McGrail M, Russell D, et al. Duration and setting of rural immersion during the medical degree relates to rural work outcomes. *Med Educ*. 2018;52(8):803-815. doi:10.1111/medu.13578

263 Shah TI, Milosavljevic S, Bath B. Determining geographic accessibility of family physician and nurse practitioner services in relation to the distribution of seniors within two Canadian Prairie Provinces. *Soc Sci Med*. 2017;194:96-104. doi:10.1016/j.socscimed.2017.10.019

264 Strasser RP, Hays RB, Kamien M, Carson D. Is Australian rural practice changing? Findings from the National Rural General Practice Study. *Aust J Rural Health*. 2000;8(4):222-226. doi:10.1046/j.1440-1584.2000.00305.x

271 Gupta S, Ngo H, Burkitt T, Puddey I, Playford D. Survival analysis of Rural Clinical School of Western Australia graduates: the long-term work of building a long-term rural medical workforce. BMC Heal Serv Res 2019;19(1):998.

272 Jamar E, Newbury J, Mills D. Early career location of University of Adelaide rural cohort medical students. Rural Remote Health. 2014;14:2592.

277 Joyce C, Wolfe R. Geographic distribution of the Australian primary health workforce in 1996 and 2001. Aust New Zeal J Public Heal. 2005;29(2):129–35.

286 Longenecker RL, Andrilla CHA, Jopson AD, Evans D V, Schmitz D, Larson EH, et al. Pipelines to Pathways: Medical School Commitment to Producing a Rural Workforce. J Rural Health. 2020

296 Shires L, Allen P, Cheek C, Deb W. Regional universities and rural clinical schools contribute to rural medical workforce, a cohort study of 2002 to 2013 graduates. Rural Remote Health. 2015;15(3):3219.

300 Sen Gupta T, Woolley T, Murray R, Hays R, McCloskey T. Positive impacts on rural and regional workforce from the first seven cohorts of James Cook University medical graduates. Rural Remote Health. 2014;14:2657

306 Smith T, Sutton K, Beauchamp A, Depczynski J, Brown L, Fisher K, et al. Profile and rural exposure for nursing and allied health students at two Australian Universities: A retrospective cohort study. Aust J Rural Health. 2021;29(1):21–33.

307 Woolley T, Sen Gupta T, Murray R, Hays R. Predictors of rural practice location for James Cook University MBBS graduates at postgraduate year 5. Aust J Rural Health. 2014;22(4):165–71.

308 Brockwell D, Wiel, t T, Clark M. Four years after graduation: occupational therapists' work destinations and perceptions of preparedness for practice. J Rural Health 2009

309 Godwin D, Blizzard L, Hoang H, Crocombe L. Evidence of the effect of rural background on rural practise in Australian dental practitioners: Does gender play a role? Aust Dent J. 2017;62(1):30–8.

311 Shah TI, Milosavljevic S, Bath B. Determining geographic accessibility of family physician and nurse practitioner services in relation to the distribution of seniors within two Canadian Prairie Provinces. Soc Sci Med. 2017;194:96–104.

312 Humphreys JS, McGrail MR, Joyce CM, Scott A, Kalb G. Who should receive recruitment and retention incentives? Improved targeting of rural doctors using medical workforce data. Aust J Rural Health. 2012;20(1):3–10.

314 Smith JL. Examination of the relative importance of hospital employment in non-metropolitan counties using location quotients. Rural Remote Health. 2013;13(3):2497.

315 Runge CE, MacKenzie A, Loos C, Waller M, Gabbett M, Mills R, et al. Characteristics of Queensland physicians and the influence of rural exposure on practice location. Intern Med J. 2016;46(8):981–5.

318 Chisholm M, Russell D, Humphreys J. Measuring rural allied health workforce turnover and retention: what are the patterns, determinants and costs? Aust J Rural Health. 2011;19(2):81–8.

324 Wan N, Zou B, Sternberg T. A three-step floating catchment area method for analyzing spatial access to health services. Int J Geogr Inf Sci. 2012;26(6):1073–89.

327 Willie-Stephens J, Kruger E, Tennant M. Public and private dental services in NSW: a geographic information system analysis of access to care for 7 million Australians. N S W Public Heal Bull. 2014;24(4):164–70.

329 Russell DJ, Humphreys JS, McGrail MR, Cameron WI, Williams PJ. The value of survival analyses for evidence-based rural medical workforce planning. Hum Resour Heal. 2013;11:65.

332 Department of Primary Industries and Energy, Department of Human Services and Health. Rural, remote and metropolitan areas classification 1991 census edition. 1994;(November).

333 Nugent P, Ogle KR, Bethune E, Walker A, Wellman DA. Undergraduate pre-registration nursing education in Australia: a longitudinal examination of enrollment and completion numbers with a focus on students from rural and remote campus locations. Rural Remote Health. Dec;4(4):313.

334 Strasser RP, Hays RB, Kamien M. Is Australian rural practice changing? Findings from the national rural general practice study. J Rural Heal. 2000

335 Olatunde S, Leduc ER, Berkowitz J. Different practice patterns of rural and urban general practitioners are predicted by the General Practice Rurality Index. Can J Rural Med Off J Soc Rural Physicians Canada. 2007;12(2):73–80.

336 Department of Health. Measuring Remoteness: Accessibility/Remoteness Index of Australia (ARIA) Revised Edition. Occas Pap. 2001;(14):1–75.

337 Australian Statistical Geographical Classification-Remoteness Area [Internet]. [cited 2021 Jul 22]. Available from: https://www.health.gov.au/health-topics/health-workforce/health-workforce-classifications/australian-statistical-geographical-classification-remoteness-area

338 Administration HR& S. Scoring Shortage Designations [Internet]. [cited 2021 Jul 22]. Available from: https://bhw.hrsa.gov/workforce-shortage-areas/shortage-designation/scoring

339 Kippenbrock T, Buron B, Odell E, Narcisse MR. Minimal Changes and Missed Opportunities: A Decade Look at Nurse Practitioners in the Lower Mississippi River Delta States. J Prof Nurs. Jun;30(3):266–72.

340 Health AG department of. Modified Monash Model [Internet]. [cited 2021 Jul 22]. Available from: https://www.health.gov.au/health-topics/health-workforce/health-workforce-classifications/modified-monash-model

341 Drov, i A, Woolley T. Workforce supply of pharmacists in Queensland communities from James Cook University Pharmacy Graduates. Aust J Rural Heal. 28(5):462–8.

342 Service UD of AER. Rural Urban Community Codes [Internet]. [cited 2021 Jul 22]. Available from: https://www.ers.usda.gov/data-products/rural-urban-continuum-codes/

343 Waldorf B. Measuring rurality. InContext. 2007;8(1):5–8.

344 ABS. Socio-Economic Indexes for Areas [Internet]. [cited 2021 Jul 22]. Available from: https://www.abs.gov.au/websitedbs/censushome.nsf/home/seifa

345 Wang F, Luo W. Assessing spatial and nonspatial factors for healthcare access: Towards an integrated approach to defining health professional shortage areas. Heal Place. 2005;11(2):131–46.

346 McGrail MR, Humphreys JS. Spatial access disparities to primary health care in rural and remote Australia. Geospat Heal. 2015;10(2):358.

347 Isserman AM. In the National Interest: Defining rural and urban correctly in research and public policy. International regional science review. 2005;28(4):465-499.

348 Rolfe IE, Pearson SA, O'Connell DL, Dickinson JA. Finding solutions to the rural doctor shortage: the roles fo selection versus undergraduate medical education in Newcastle. Aust NZJ Med.. 1995;25(5):512-517.

349 Bear LD, Johnson-Webb KD, Gesler WM. What is rural? A focus on urban influence codes. J Rural Health. 1997;13(4):329-333.

350 Kane KY, Quinn KJ, Stevermer JJ, Porter JL, Webb WD, Williamson Jr HA, Burdin J. Summer in the country: changes in medical students' perceptions following an innovative rural community experience. Acad Med. 2013;88(8)1157-1163.
